# Engineering Carbon Cloth‐Catalyst Interfaces for Pt‐Free Hydrogen Evolution Across the Full pH Spectrum

**DOI:** 10.1002/advs.77763

**Published:** 2026-10-04

**Authors:** Syed Mohammed Hubaish, Belal Salah, Khouloud Jlassi, Ali A. Abdelhafiz, Kamel Eid

**Affiliations:** ^1^ Gas Processing Center (GPC) College of Engineering Qatar University Doha Qatar; ^2^ Center for Advanced Materials Qatar University Doha Qatar; ^3^ Department of Nuclear Science Engineering Massachusetts Institute of Technology Cambridge Massachusetts USA

**Keywords:** Hydrogen evolution reaction, Carbon cloth electrodes, Pt‐free electrocatalysts, Interface Engineering, Transition metal catalysts, Green hydrogen

## Abstract

The development of Pt‐free electrocatalysts is critical for reducing the cost of hydrogen production through the hydrogen evolution reaction (HER). Although extensive efforts have focused on improving the intrinsic activity of earth‐abundant catalysts, the role of the catalyst–support interface remains comparatively underexplored. Carbon cloth (CC) has emerged as an attractive electrode platform owing to its electrical conductivity, hierarchical porosity, mechanical flexibility, chemical stability, and ability to facilitate efficient charge and mass transport. Although CC is generally not the primary HER active phase, it can strongly influence electrode‐level performance by regulating interfacial electron transfer, catalyst anchoring, active‐site accessibility, wettability, hydrogen bubble release, and long‐term durability. This review critically analyzes the interfacial synergy between CC and Pt‐free transition‐metal‐based catalysts, including Co‐, Ni‐, Fe‐, Cu‐, Mo‐, and W‐based systems, with and without heteroatom or anion modification involving N, P, and S species. The HER activity and stability of these systems are evaluated under acidic, alkaline, neutral, and pH‐universal conditions. A particular emphasis is placed on how catalyst–support interactions, synthesis strategies, surface chemistry, structural features, and mass‐transport behavior collectively determine electrochemical performance. Finally, current challenges and future opportunities are discussed, including mechanistic understanding, operando characterization, high‐current‐density operation, long‐term durability, scalable fabrication, and practical electrolyzer integration.

## Graphical abstract

Interfacial synergy between carbon cloth and Pt‐free metal/nonmetal catalysts enhances charge transfer, wettability, active site accessibility, and hydrogen bubble release, enabling efficient and durable hydrogen evolution across acidic, neutral, and alkaline media for scalable green hydrogen production.

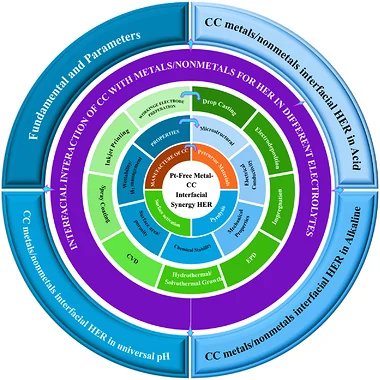

## 1 Introduction

The transition from fossil fuel–based energy systems to sustainable energy technologies is essential for mitigating carbon emissions and addressing global climate challenges [1]. Among the available clean energy vectors, green hydrogen has emerged as a promising candidate owing to its high gravimetric energy density, carbon‐neutral utilization, and compatibility with long‐term energy storage and transportation [2, 3, 4, 5, 6, 7, 8]. In this context, water electrolysis represents one of the most attractive routes for sustainable hydrogen production, with the hydrogen evolution reaction (HER) serving as the key cathodic process [8, 9, 10]. Despite significant technological progress, the widespread deployment of HER‐based systems remains constrained by catalyst cost, efficiency, and durability [11, 12, 13].

Platinum remains the benchmark HER catalyst because of its near‐optimal hydrogen adsorption energy, rapid reaction kinetics, and excellent stability across a wide pH range. However, the scarcity and high cost of Pt substantially increase electrolyzer capital costs and limit large‐scale implementation [14, 15, 16, 17, 18, 19, 20, 21, 22, 23, 24]. Consequently, extensive efforts have been devoted to developing Pt‐free HER catalysts based on earth‐abundant transition metals, including Ni, Co, Fe, Cu, Mo, and W, as well as their oxides, hydroxides, phosphides, sulfides, selenides, nitrides, carbides, and multicomponent derivatives [24, 25, 26, 27, 28]. Although considerable progress has been achieved through compositional tuning, heteroatom doping, defect engineering, and heterostructure design, many Pt‐free catalysts still suffer from limited electrical conductivity, sluggish charge‐transfer kinetics, insufficient active‐site utilization, and inadequate durability under practical operating conditions [27, 28].

Beyond catalyst composition, increasing attention has been directed toward the role of electrode substrates in determining overall HER performance. Carbon‐based materials, including graphene, carbon nanotubes, carbon fibers, carbon paper, and carbon cloth, have been widely explored as conductive supports because of their favorable electrical and physicochemical properties [29, 30, 31]. Among these platforms, carbon cloth has emerged as a promising HER electrode substrate owing to its electrical conductivity, hierarchical porosity, mechanical flexibility, chemical stability, and three‐dimensional interconnected architecture [32, 33, 34, 35, 36]. These characteristics facilitate electron transport, electrolyte diffusion, catalyst integration, and hydrogen bubble release, all of which are important for achieving high catalytic activity and long‐term stability [35, 36].

Importantly, carbon cloth should not be viewed merely as a passive current collector. Although bare CC generally exhibits limited intrinsic HER activity, it can strongly influence electrode‐level catalytic performance through interfacial interactions with the supported catalyst. These interactions affect electron transfer, catalyst dispersion, active‐site accessibility, wettability, gas‐evolution dynamics, and structural stability. Strong catalyst–CC coupling can enable binder‐free electrode architectures, reduce interfacial resistance, improve catalyst utilization, and suppress catalyst detachment during prolonged operation. Therefore, rational engineering of the catalyst–carbon cloth interface has become a critical design strategy for developing high‐performance Pt‐free HER electrodes [37, 38, 39, 40].

Despite the rapidly growing number of reports on Pt‐free catalysts supported on carbon cloth, the mechanistic role of catalyst–support interactions remains insufficiently understood. Many studies primarily emphasize catalyst composition and intrinsic activity, while the contributions of interfacial chemistry, charge redistribution, catalyst anchoring, wettability, and mass transport are often discussed only qualitatively. Furthermore, a comprehensive assessment of these interfacial effects across acidic, alkaline, neutral, and pH‐universal HER systems remains limited [40, 41, 42, 43, 44, 45]. Therefore, this review focuses on the interfacial synergy between carbon cloth and Pt‐free transition‐metal‐based HER catalysts (Figure 1). Particular emphasis is placed on how interfacial engineering, synthesis strategies, structural design, and electrolyte environment govern HER activity and durability. The review also discusses mechanistic aspects of catalyst–CC interfaces, degradation pathways, stability‐enhancement strategies, industrially relevant current densities, catalyst loading, scalability, and electrolyzer integration. Finally, current challenges and future opportunities are discussed to provide design guidelines for scalable, cost‐effective, and durable HER electrodes for practical green hydrogen production.

**FIGURE 1:**
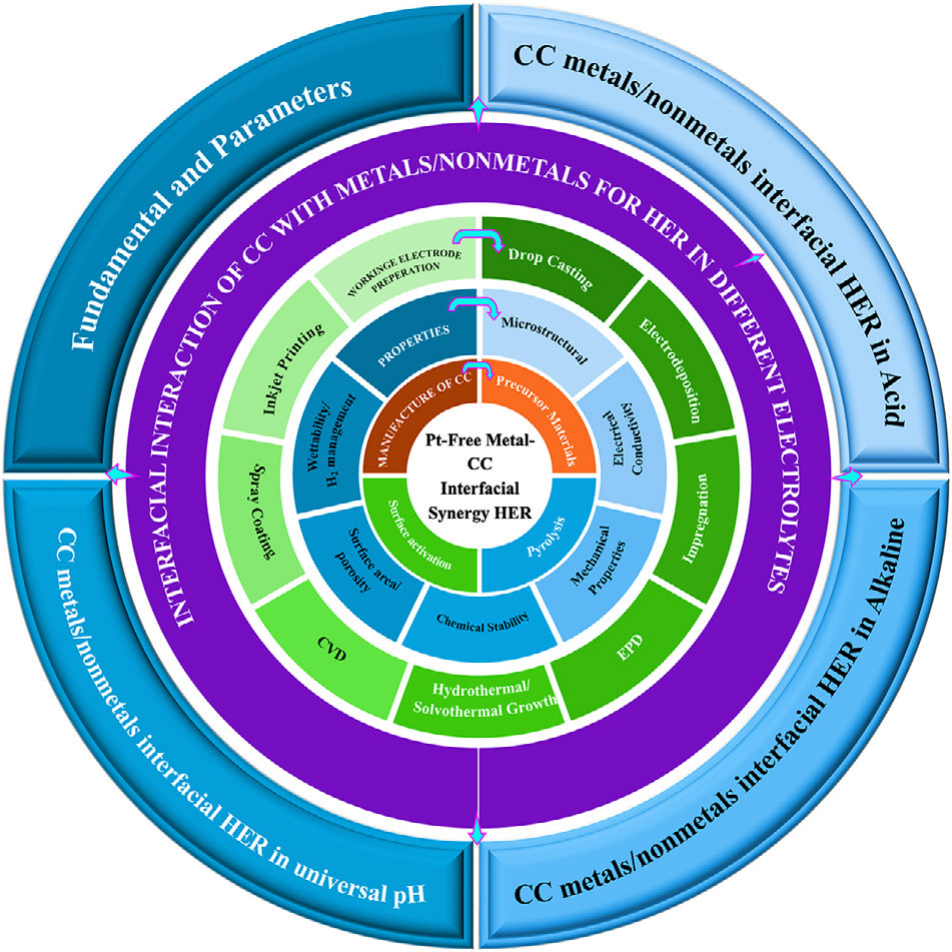
Schematic showing the focal points of the presented review study.

## 2 Manufacturing of Carbon Cloth

The fabrication process and type of precursors determine the properties of CC, so understanding this relationship is essential to tailor the electrochemical HER performance. The following sections discuss the main precursors and fabrication process, as well as activation approaches.

### 2.1 Precursor Materials

The precursor fibers that are woven into fabric before carbonization include green materials such as rayon, pitch, cellulose, and polyacrylonitrile (PAN), but PAN‐based carbon fibers account for over 90% of the global CC fiber market. PAN is a synthetic polymer (C_3_H_3_N)_n_ with rich nitrile groups (─C≡N) pendant to the polymer chain, and during carbonization under heating, nitrile groups undergo cyclization reactions that form thermally stable, nitrogen‐containing aromatic carbon structures [46, 47, 48]. This form of CC with N‐atoms in the lattice modifies both electronic structure and surface chemistry, and subsequently, CC possesses higher tensile strength, greater electrical conductivity, and better crystallite orientation compared to other types. However, the relatively high cost of PAN is a crucial issue. Cellulose‐based materials are sustainable, earth‐abundant, and can be carbonized directly to form graphitic carbon via dehydration and depolymerization processes, but they form amorphous carbon without heteroatoms and with less electrical conductivity than PAN‐based materials [46, 47, 48]. Recently, we found that impregnating cellulose fiber with metal precursors, followed by annealing under nitrogen, formed active carbon fiber in situ decorated with Pt‐free metal nanocrystals, and then coupling the thus‐formed fiber with CC led to outstanding HER activity closer to Pt/C. This is due to interfacial interaction, better electrical conductivity, and wettability during HER.

### 2.2 Pyrolysis

Pyrolysis is the main preparation method for the conversion of carbon precursors into CC fiber, but it must maximize carbon yield, graphitization, preserve mechanical strength, and achieve the needed properties (i.e., electrical properties and surface area) [49, 50, 51]. The process starts with an initial stabilization step at 200–300°C in air to provide oxidative stabilization of precursors via removal of water vapor (H_2_O), followed by depolymerization in the case of cellulose or cycling of PAN polymer chains to form ladder‐like structures. The rich oxygen groups in the precursors promote crosslinking reactions that maintain fiber morphology and prevent melting or fusion during heating, which can continue from a few minutes (i.e., 30 min) to several hours, depending on fabric thickness and the required stabilization depth. This is followed by the carbonization step at 1000–1500°C in an inert atmosphere (i.e., N_2_ or Ar) to eliminate non‐carbon elements (i.e., H_2_, O_2_, and N_2_) as volatile gases, followed by reorganization of carbon atoms into turbostratic structures of small, imperfect graphene‐like sheets stacked with limited long‐range order [52, 53]. The electrical conductivity increases during this stage as conjugated π‐systems develop across the carbon network, and it could be improved by achieving a higher degree of graphitization at 2000–3000 °C, which allows turbostratic carbon domains to grow and align more closely with a crystalline graphite structure. However, this step is energy‐demanding, increases the cost, and reduces mechanical flexibility, so in HER, graphitized CC materials are not preferred compared with carbonized CC. Note that holding woven fabric under restrained conditions at heating rates of 50–200°C/h during the stabilization step prior to carbonization at 1000–1600 °C can improve electrical conductivity and durability by preventing shrinkage and distortion.

### 2.3 Surface Activation

After fabrication, CC materials are relatively inert, hydrophobic (i.e., water contact angle ∼120°), and have a low surface area, which limits their wettability and use in HER, so they should be activated. This includes thermal activation in air or CO_2_ at 400–600 °C to introduce oxygen‐containing functional groups (i.e., OH, CO, and CO_2_) while creating additional surface roughness, which improves hydrophilicity (i.e., water contact angle < 30°) [54, 55, 56]. Additionally, oxygenated functional groups can serve as anchoring sites for catalyst precursors, promoting uniform deposition while enhancing wettability. Chemical activation with strong acids (e.g., HNO_3_) or oxidizing solutions can introduce nitrogen‐ and oxygen‐bearing groups, while etching amorphous carbon to increase surface area; however, such chemicals are hazardous and can affect fiber integrity [57]. Electrochemical activation includes the utilization of a CC electrode as a working electrode in two‐/three‐electrode chemical cells and then applying potential cycling in acidic (i.e., H_2_SO_4_ and HClO_4_) or alkaline (i.e., KOH) electrolytes [58, 59]. This method is simple, green, fast, and offers precise control over surface chemistry and can be integrated directly into electrode preparation workflows without additional steps. Activated CC materials demonstrate that activated CC materials have higher catalyst loadings, lower charge transfer resistance, and enhanced durability during operation compared to pristine CC. Also, coupling hydrophilicity with micro‐roughness accelerates H_2_ bubble detachment, thereby reducing mass transport overpotentials at practical current densities.

## 3 Properties of Carbon Cloth

Carbon cloth materials have outstanding physicochemical merits that make them efficient electrode substrates for water splitting (HER and OER), with performance derived from the synergistic interplay of several key properties. Understanding such properties (i.e., electrical conductivity, surface area/porosity, stability, wettability, and gas management) can enable rational electrode design and HER performance optimization. To better understand the advantages and limitations of carbon cloth as an electrocatalytic substrate, a comparison is made with other widely used electrode platforms, including nickel foam, carbon paper, graphite felt, titanium mesh, stainless steel, and copper foam (Table 1). This comparison highlights the differences in electrical conductivity, structural characteristics, chemical stability, catalyst anchoring capability, and suitability for practical electrochemical applications.

**TABLE 1:** Comparison of carbon cloth with other commonly used electrode substrates for electrocatalysis.

Electrode substrate	Advantages	Limitations
Carbon cloth (CC)	Lightweight, flexible, highly conductive, corrosion‐resistant in acidic/neutral media, 3D porous structure, binder‐free catalyst growth, tunable surface chemistry	Lower intrinsic catalytic activity than some metal substrates, oxidation under harsh anodic conditions, relatively lower mechanical strength
Nickel foam (NF)	Excellent conductivity, high surface area, robust 3D structure, strong mechanical strength, favorable for alkaline HER/OER	Ni can participate in catalytic reactions, difficult to distinguish substrate and catalyst contributions, corrosion/oxidation in some electrolytes.
Carbon paper (CP)	High electrical conductivity, smooth and uniform surface, good gas diffusion properties, widely used in fuel cells and electrolyzers	Less flexible than CC, lower surface roughness and anchoring ability, weaker catalyst loading compared with porous substrates
Graphite felt (GF)	Highly porous, excellent chemical stability, large electrolyte‐accessible surface area, suitable for flow systems	Lower conductivity compared with some carbon substrates; a thicker structure can increase mass‐transfer resistance
Titanium mesh (Ti mesh)	Outstanding corrosion resistance, high mechanical stability, suitable for harsh oxidative environments (OER)	Higher cost, limited surface functional groups for catalyst anchoring, lower flexibility
Stainless steel (SS)	Low cost, high mechanical strength, good conductivity, scalable fabrication	Surface corrosion, possible metal dissolution, substrate contribution to catalytic activity can complicate analysis.
Copper foam (Cu foam)	High conductivity, 3D porous architecture, suitable for Co_2_ reduction and some HER systems	Poor stability in oxidative environments, Cu dissolution, possible catalytic interference

### 3.1 Microstructural

CC materials have a hierarchical structure across multiple length scales: carbon fibers (∼5–10 µm in diameter), which are woven into textile architectures (i.e., plain weave, twill, or satin), and each fiber consists of thousands of turbostratic carbon crystallites oriented preferentially along the fiber axis [60, 61, 62]. The microstructure determines properties, macroscopic porosity, and mechanical behavior, maximizing axial conductivity while maintaining radial strength. This textile structure possesses intrinsic micropores (<2 nm) and mesopores (2–50 nm) within individual fibers, and macropores between fibers (∼10–100 µm) that arise from the woven architecture, which serve as electrolyte reservoirs and H_2_ gas release channels.

### 3.2 Electrical Conductivity

Carbon cloth exhibits electronic conductivities ranging from nearly 10 to 100 S/cm for standard carbon grades, with graphitized variants at about 200–300 S/cm [60, 61, 62, 63]. Although these values are orders of magnitude lower than Cu/Ag (∼5.9 × 10^5^ S/cm), carbon cloth provides suitable conductivity for the HER because the planar resistance of a CC electrode (∼0.5–2 Ω/square) is small relative to charge transfer resistances at the catalyst‐electrolyte interface (∼10–100 Ω) [53, 64]. Also, the 3D network structure of cloth provides multiple parallel conduction pathways, reducing effective resistance, while the thinness of CC electrodes (typically 0.3–0.5 mm) shortens the electron path length. When the CC surface is functionalized with metals, electron transfer and interactions with the metal surface can promote HER. However, CC conductivity influences the distribution of electrochemical potential across large‐area electrodes, which becomes critical when scaling from laboratory‐scale (1 cm^2^) to industrial‐scale (over 100 cm^2^) electrodes. So, optimization of HER performance is needed at both the lab and practical scales.

### 3.3 Mechanical Properties

CC textile materials have impressive mechanical flexibility (i.e., bending, rolling, and folding) without fracturing, which makes them suitable for commercial flexible and portable devices. For instance, CC derived from PAN has tensile strength (∼3–5 GPa) that exceeds that of steel on a weight‐for‐weight basis; meanwhile, the cloth architecture distributes mechanical loads across all fibers homogeneously [53, 60, 61, 62]. This flexibility is more than just a property; it is an enabling feature, as CC can be incorporated into roll‐to‐roll manufacturing processes, conform to curved reactor geometries, and withstand the mechanical stresses of flowing electrolytes in practical electrolyzers. Moreover, the ease of CC cutting into arbitrary shapes without edge chipping or delamination simplifies working electrode fabrication for the HER.

### 3.4 Chemical Stability

Carbon cloth demonstrates exceptional chemical resistance over all pH ranges during electrolysis. Under acidic conditions (i.e., 0.5 M H_2_SO_4_, pH ∼0), CC remains stable over thousands of hours of operation, and in alkaline solutions (i.e., 1 M KOH, pH ∼14), CC resists corrosion, which is crucial for practical water electrolyzers [60, 61, 62, 65]. This stability arises from the graphitic‐like carbon fiber structure, comprising an sp^2^‐hybridized carbon network that is thermodynamically stable under reducing potentials and resists oxidation except at ultra‐high positive potentials. The pH‐universal stability is impressive in contrast to other metal‐based substrates (i.e., Ni‐foam that corrodes in acid and stainless steel that passivates and leaches ions). Moreover, during the HER, CC is inert and cannot split water or undergo any side reactions, ensuring that the obtained HER activity originates from the catalyst.

### 3.5 Surface Area and Porosity

The specific surface area of CC is tunable via activation state using different methods. Pristine CC usually possesses a surface area of 5–20 m^2^/g that can be increased to 200–600 m^2^/g after activation via insertion of multimodal porosity (i.e., microporosity, macroporosity, and mesoporosity). For perspective, a CC (1 cm^2^, 0.3 mm thick, density ∼0.5 g/cm^−3^) presents an internal surface area comparable to a postage stamp (5‐10 cm^2^) in its geometric estimate but contains 30–90 m^2^ of actual surface area equal to a room‐sized area [60, 61, 62, 66]. The high surface area not only provides abundant sites for catalyst anchoring but also makes the catalysts' active sites accessible during HER. CC can be easily doped with other heteroatoms, leading to defect sites that can participate in the HER. CC materials have intrinsic micropores (<2 nm) and mesopores (2–50 nm) that arise from individual fibers during carbonization [60]. The porosity maximizes utilization of catalyst active sites, eases electrolyte transport, and improves electrode‐electrolyte interaction during HER. However, rich microporosity can trap H_2_ gas bubbles, impede electrolyte transport, and host undercoordinated carbon sites that are vulnerable to oxidation; thus, optimized surfaces and porosity are crucial for enhanced HER performance.

### 3.6 Wettability and H2 Gas Management

The relationship between working electrode wettability and H_2_ gas evolution is a decisive factor for promoting HER performance. Under negative voltage, the H_2_ bubbles nucleate at active sites, grow through coalescence, and eventually detach when buoyancy exceeds adhesive forces [67, 68, 69]. Thus, H_2_ accumulates on the electrode surface, blocking active sites, increasing ohmic resistance, and reducing mass transport, so CC, with its spatial porous structure, provides rich channels for easing the release of H_2_ gas bubbles, reducing residence times compared to planar electrodes, and easing electrolyte diffusion within the electrode, resulting in maintained wettability and subsequent H_2_ bubble detachment. Surface functionalization can enhance wettability to optimize H_2_ bubble dynamics, moderate hydrophilicity, and ease electrolyte transport, besides enabling H_2_ bubble detachment as confirmed via in situ microscopy and electrochemical EIS.

## 4 Working Electrode Preparation

The preparation of the working electrode is a critical step in the electrocatalytic hydrogen evolution reaction (HER), as it directly influences catalytic activity, stability, and reproducibility. Carbon cloth is widely used as a substrate due to its high conductivity, flexibility, large surface area, and excellent chemical stability. Various methods exist for fabricating carbon cloth–based working electrodes, each offering distinct advantages and limitations (Table 2).

**TABLE 2:** Comparison of the fabrication methods of catalyst ink over a CC working electrode.

Method	Uniformity	Loading Control	Adhesion	Scalability	Binder Needed
Drop Casting	Poor	Poor	Weak	High	Usually
Electrochemical Deposition	Excellent	Excellent	Strong	Moderate	No
Impregnation	Good	Poor	Weak	High	Usually
Electrophoretic Deposition (EPD)	Excellent	Good	Moderate (needs annealing)	Moderate	No
Hydrothermal Growth	Excellent	Good	Very strong	Low	No
CVD	Excellent	Excellent	Very strong	Low	No
Spray Coating	Good	Good	Moderate	High	Often
Inkjet Printing	Excellent	Excellent	Moderate	Moderate	Often

### 4.1 Drop Casting

The simplest method involves pipetting a catalyst ink (catalyst + binder + solvent) onto carbon cloth [70]. However, it often leads to inhomogeneous catalyst distribution, poor control over loading, and the risk of catalyst detachment or dissolution during HER, especially under gas evolution.

### 4.2 Electrodeposition

A more sophisticated technique involves depositing the catalyst from an electrolyte solution onto the carbon cloth by applying a controlled potential or current. It offers uniform distribution, precise loading control (via deposition time, voltage, current density, and electrolyte composition), strong catalyst–substrate adhesion, enhanced charge transfer, and excellent stability [71, 72]. The main drawback is the need for specialized equipment and optimization of deposition parameters.

### 4.3 Impregnation

The carbon cloth is immersed in a catalyst ink or precursor solution for a set time, then dried. This allows relatively homogeneous distribution within the porous structure but suffers from poor loading control, often requiring multiple cycles. Without a binder, the catalyst can easily leach out, raising stability concerns [73, 74].

### 4.4 Electrophoretic Deposition (EPD)

In EPD, charged catalyst particles in suspension migrate under an electric field and deposit onto the conductive carbon cloth [75, 76]. Also, EPD provides uniform, thickness‐controlled coatings; is suitable for a wide range of materials (e.g., graphene, MoS_2_, and transition metal dichalcogenides); and avoids the need for binders; however, it requires stable colloidal suspensions and post‐deposition annealing to improve adhesion.

### 4.5 Hydrothermal/Solvothermal Growth

The carbon cloth is placed in an autoclave with a precursor solution and heated (typically 120–220°C) to grow catalyst nanostructures directly onto the fibers. This method yields strongly adhered, binder‐free, and highly crystalline catalysts with controlled morphology (nanowires, nanosheets, etc.) [77, 78, 79]. Drawbacks include high pressure/temperature, long reaction times (several hours to days), and limited scalability.

### 4.6 Chemical Vapor Deposition (CVD)

Gaseous precursors decompose on the heated carbon cloth surface to deposit thin films or nanostructures (e.g., MoS_2_, WS_2_, graphene) [80, 81, 82, 83]. CVD produces high‐purity, uniform, and conformal coatings with excellent adhesion. However, it requires expensive equipment, high temperatures, and vacuum systems, and is less suitable for temperature‐sensitive catalysts.

### 4.7 Spray Coating

A catalyst ink is atomized through a nozzle and sprayed onto the carbon cloth. This method allows large‐area, uniform deposition with controllable loading (by adjusting spray time, flow rate, and nozzle distance) [82, 84, 85, 86]. It is scalable and compatible with roll‐to‐roll manufacturing. Disadvantages include possible catalyst waste, the need for a binder to improve adhesion, and potential nozzle clogging.

### 4.8 Inkjet Printing

A digital printing technique involves precisely depositing picoliter droplets of catalyst ink are precisely deposited onto the carbon cloth. It enables high‐resolution patterning, minimal material waste, excellent reproducibility, and automated fabrication [87, 88]. However, it requires low‐viscosity inks and specialized printers and may suffer from nozzle clogging; it is also less suitable for very high loadings.

## 5 Benefits of Transition Metals Pt‐Free Metals

Replacing platinum with earth‐abundant transition metals (e.g., Ni, Co, Fe, Mo, W) offers several key advantages for the HER, including low cost, natural abundance, sustainability, and limited environmental impact [89, 90]. Pt costs ∼$55K/kg, which is intolerable compared with other transition metals (i.e., Ni and Fe) [91]. Thus, using Pt‐free metals not only reduces cost by more than 90% but also reduces environmental impact due to high energy demand and CO_2_ emissions for mining and refining of Pt‐based (i.e., ∼40–50 tons CO_2_/kg Pt) [92]. In contrast, the mining and refining of transition metals have lower energy demands and significantly lower extraction‐related emissions per kilogram, especially for Fe and Ni. For the HER, some transition metals (e.g., Ni, Co, Mo) have near‐optimal binding strength with hydrogen, minimizing overpotential, especially when coupled together or with heteroatoms [93]. In addition to the stability of these metals in alkaline/acidic solutions, proper alloying or mixing with other non‐metals (e.g., Ni‐Mo, CoP, FeS_2_) can also facilitate efficient electron transfer during HER [94]. Although Pt‐based cathodes have near‐zero η_10_ (∼0–20 mV), compared with 50–300 mV for transition‐metal cathodes, transition‐metal cathodes can show excellent long‐term stability (e.g., >100 h) [93]. Thus, using Pt‐free metals with CC can enhance the performance to meet the requirements of industrial water electrolysis and green hydrogen production.

## 6 Parameters Affecting Activity

Various factors determine the performance of the HER, including overpotential (η), Tafel slope, charge transfer resistance, current density, turnover frequency (TOF), iR‐corrected overpotential, and faradaic efficiency. Numerous reviews highlight the impact of these factors on HER. Typically, a high current density and TOF at low η are preferred for the HER [41, 95, 96]; meanwhile, IR correction is necessary before reporting the activity. Additionally, understanding the interplay between these factors can lead to the development of more efficient catalysts, which can significantly improve the efficiency and cost‐effectiveness of hydrogen production processes. Optimizing these parameters is crucial for enhancing the overall HER performance.

## 7 Interfacial Interaction of CC with Metals/Nonmetals in H_2_SO_4_

The interfacial interaction between carbon cloth and various metal/nonmetal catalysts plays a pivotal role in enhancing HER performance across all pH. Carbon cloth serves as an excellent conductive and mechanical support that facilitates electron transport and provides active sites. The HER activity, kinetics, and stability are determined by various strategies for engineering these interfaces, including different synthesis approaches, the synergy between the catalytic materials and the carbon cloth substrate, and the intrinsic properties of the catalysts. In Table 3, a summary of the activity of various catalysts in H_2_SO_4_ using carbon cloth electrode substrates is provided.

**TABLE 3:** Comparison of the HER performance of CC‐supported metals/nonmetals in H_2_SO_4_.

Catalyst	Preparation method	η_10_ (mV)	Tafel slope (mV/dec)	Current density	Stability (h/%)	References
CoP NPs/CC	Impregnation, calcination at 600 °C, and phosphidation	48	70	∼−100 mA/cm^2^ @ −0.2 V	30 h	[97]
MoSe_2_/CC‐60	Electrophoretic deposition (EPD)	220	76	∼−65 mA/cm^2^ @−0.32 V	100 h	[98]
Mo–W–P/CC	Hydrothermal growth and phosphidation at 700 °C.	138 @ 100 mA/cm^2^	52	∼−250 mA/cm^2^ @ −0.17 V	—	[99]
MoO_2_/MoSe_2_	Chemical Vapor Deposition (CVD) process	181	49.1	∼−30 mA cm^2^ @ −0.2V	8 h	[100]
CoNOC//CFC‐900	Pyrolysis route	69	46	∼−80 mA/cm^2^ @−0.15 V	8 h	[101]
CoSe_2_/CF	Electrodeposition, Hydrothermal, and selenization.	118	53.2	∼−250 mA/cm^2^ @ −0.2 V	12 h	[102]
Fe‐WO_x_P/rGO	Hydrothermal growth, phosphine reduction	54.6	41.99	∼−100 mA/cm^2^ @ −0.17 V	24 h	[103]
MoS_x_/CF/CC‐2	Carbonization and hydrothermal synthesis.	127	51	∼−500 mA/cm^2^ @ −0.26 V	20 h	[104]
Ni/NiO/TiO_2_/rGO@CC	Plasma‐enhanced chemical vapor deposition (PECVD) technique	130	40	∼−10 mA/cm^2^ @ −0.15 V	60 h	[105]
MWCNTs/MoS_2_	Hydrothermal method	211	136	∼−100 mA/cm^2^ @ −0.55 V	22 h	[106]
MoS_2_/FeS_2_/CC	Chemical vapor deposition (CVD) method	134	76.8	∼−100 mA/cm^2^ @ −0.2 V	—	[107]
meso‐FeP film/CC	Evaporation‐induced self‐assembly (EISA), calcination, and phosphidation	65	44	∼−100 mA/cm^2^ @ −0.12 V	20 h	[108]
W_x_V_1__−__x_Se_2_/CC	Hydrothermal method	173	80	∼−30 mA cm^2^ @ −0.2 V	>5 h	[109]
P‐MoSe_0_._5_S_1_._5_/N‐CNTs/CC‐2	Hydrothermal growth and controlled phosphorization	108.3	58.6	∼−60 mA/cm^2^ @ −0.16 V	12 h	[110]
MoOx–Pd/CC	Two‐step dip‐coating and electrochemical reduction	25	45.75	∼−200 mA/cm^2^ @ −0.1 V	7 days	[111]
Pd(II)BTT/GrCC	Synthesis of metal complexes and immobilization onto carbon support	419	144	∼−10 mA/cm^2^ @ −0.2 V	6 h	[112]
Ni_3_(VO_4_)_2_/MoS_2_@CFC	Hydrothermal method	77	65	∼−150 mA/cm^2^ @ −0.3 V	12 h	[113]
Ni/Fe‐MoS_2_/CC	Hydrothermal method.	116	43	∼−20 mA/cm^2^ @ −0.15 V	15 h	[114]
7% Ru‐WN/CC	Hydrothermal method and high‐temperature nitridation	94	51.4	∼−100 mA/cm^2^ @ −0.15 V	24 h	[115]
PANI‐pf‐GCC@MoS_2_	Hydrothermal method	178	40	∼−70 mA cm^2^ @ −0.5 V	60 h	[116]
NiMoSe/Ti_3_C_2_T_x_@CC	Hydrothermal method	403	33	∼−50 mA/cm^2^ @ −0.57 V	—	[117]

Cobalt phosphide nanoparticle (NPs) films directly grown on carbon cloth (CoP NPs/CC) were synthesized via impregnation followed by calcination at 600 °C and phosphidation, creating a strongly coupled CoP NPs/carbon cloth interface that provides efficient electron transport and firm catalyst adhesion without binders. The cobalt NP precursor exhibited excellent HER performance in 0.5 M H_2_SO_4_, with a hydrogen evolution current density (*J*_HER_) of −100 mA/cm^2^ at −0.20 V, an overpotential (η_10_) at 10 mA/cm^2^ of 48 mV, and a Tafel slope of 70 mV/dec, indicating a Volmer–Heyrovsky reaction pathway with efficient hydrogen intermediate conversion [97]. In addition to maintaining stability for 30 h, durability is attributed to the strong CoP–carbon cloth interaction, which prevents catalyst detachment, maintains electron transport across the continuous nanoparticle film, and supports efficient mass transport. However, the performance remains inferior to Pt‐based catalysts without efficient benchmarking [97].

Three‐dimensional, binder‐free molybdenum diselenide nanosheets deposited on carbon cloth (MoSe_2_/CC) were synthesized via electrophoretic deposition (EPD), forming a uniform nanosheet film tightly anchored to the conductive substrate, where the uniform nanosheet deposition and strong MoSe_2_–carbon cloth coupling facilitate direct electron transport through the electrode interface [98]. The optimized MoSe_2_/CC‐60 HER in 0.5 M H_2_SO_4_, with a *J*_HER_ of −65 mA/cm^2^ at −0.32 V, η_10_ of 220 mV, and a Tafel slope of 76 mV/dec, indicates a Volmer–Heyrovsky pathway with electrochemical desorption contribution. The density of exposed MoSe_2_ edge sites and the porous 3D carbon cloth structure provide active centers for hydrogen adsorption, improve electrolyte accessibility, and the binder‐free design leads to better HER performance, as shown by EIS and electrochemical surface area measurements. The MoSe_2_/CC‐60 showed outstanding endurance, with steady catalytic activity after 1000 continuous potential cycles and low current degradation during electrolysis, due to strong catalyst–substrate interaction that prevents catalyst detachment during operation. Although stable and binder‐free, MoSe_2_/CC exhibits moderate reaction kinetics and high overpotentials due to the limited intrinsic activity of the basal planes, thereby limiting HER efficiency at low potentials and high current densities compared to advanced non‐noble metal catalysts [98].

Molybdenum tungsten oxide (Mo–W–O) precursor nanowires were hydrothermally grown on carbon cloth and phosphidated at 700°C to yield porous Mo–W–P hybrid nanosheets (Figure 2a) [99]. The SEM image in Figure 2b shows that densely packed nanosheet assemblies cover the carbon cloth fibers, establishing a porous network with a conductive bimetallic phosphide/carbon cloth interface with strong electrical integration. High‐magnification TEM and elemental mapping show a 100‐nm nanosheet thickness and consistent Mo, W, and P distribution (Figure 2c), indicating the production of a bimetallic phosphide without phase segregation. The MoWP/CC showed HER activity in 0.5 M H_2_SO_4_, with a *J*_HER_ of −250 mA/cm^2^ at −0.17 V, η_100_ of 138 mV, and a Tafel slope of 52 mV/dec, suggesting Volmer–Heyrovsky kinetics with accelerated hydrogen intermediate conversion. Electrochemical tests (Figure 2d) show that MoWP/CC has much lower overpotentials at a given *J*_HER_ than MoP/CC and WP_2_/CC, despite Pt/C/CC being the most active benchmark. The reaction kinetics of Mo–W–P/CC are faster than those of MoP/CC and WP_2_/CC, as shown by the lower Tafel slopes of 55 and 78 mV/dec, respectively, originating from synergistic electronic modulation between Mo and W sites, optimized hydrogen adsorption, and enhanced charge transfer within the bimetallic phosphide framework (Figure 2e). Stability (Figure 2f) shows no performance degradation after 4000 cycles, proving that the catalyst is strongly attached to carbon cloth, contributing to long‐term stability by preventing structural collapse and catalyst loss. The MoWP/CC exhibits stability in acidic environments, but requires an η_100_ of 138 mV and high‐temperature phosphidation at roughly 700°C [99]. Compared to Pt‐based catalysts, this makes synthesis more complicated and energy‐intensive, making practical use challenging.

**FIGURE 2:**
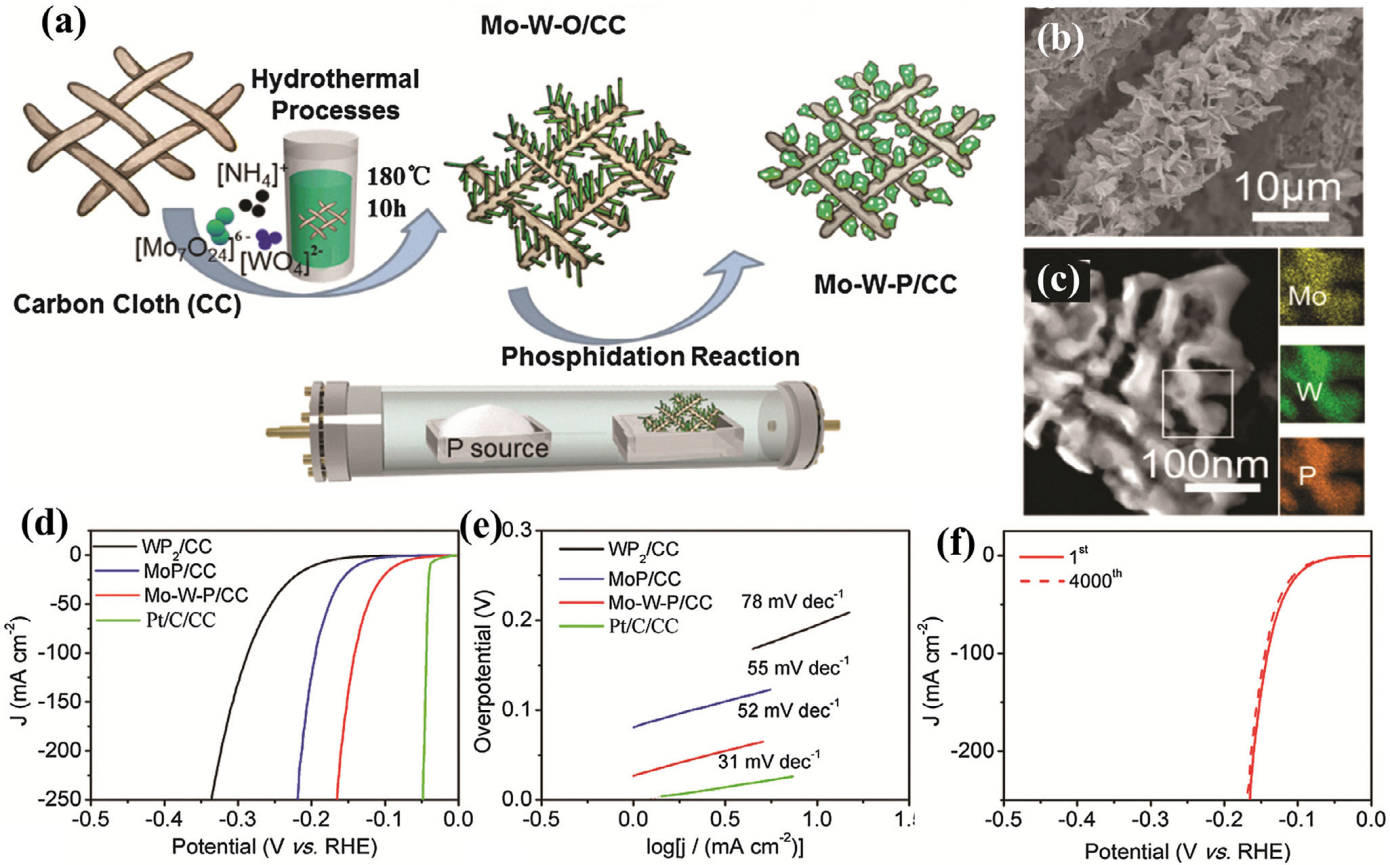
(a) Schematic illustration of the fabrication of Mo–W–P/CC, (b) SEM image, (c) HAADF‐STEM image with corresponding elemental‐mapping images of Mo–W–P/CC, (d) LSV, (e) Tafel slope of Mo–W–P/CC compared with others, and (f) LSV before and after stability testing of Mo–W–P/CC in 0.5 M H_2_SO_4_. Copyright 2016 RSC, Ref. [99].

2D MoO_2_/MoSe_2_ core–shell nanosheet arrays grown directly on carbon cloth (MoO_2_/MoSe_2_‐CC) were synthesized via a chemical vapor deposition approach, where the MoO_2_/MoSe_2_ interface promotes charge redistribution and improves interfacial electron transport [100]. MoO_2_/MoSe_2_‐CC showed a decent efficiency in HER in 0.5 M H_2_SO_4_, with a *J*_HER_ of 30 mA/cm^2^ at −0.20 V, η_10_ of 181 mV, and a Tafel slope of 49.1 mV/dec, indicating favorable Volmer–Heyrovsky kinetics with efficient hydrogen intermediate conversion. It also demonstrated stability over 8 h, related to the strong adhesion between the catalyst and carbon cloth, which maintains structural integrity during electrolysis. In contrast, despite the improved reaction rates, the *J*_HER_ at low overpotentials remains moderate compared with the best available catalysts. This suggests that further improvement in the inherent activity of the active sites is necessary for high‐current industrial applications [100].

Cobalt nanocrystals encapsulated in N, O‐dual‐doped porous carbon frameworks (CoNOCs) supported on carbon fiber cloth were synthesized via chemical deposition of a conjugated microporous polymer precursor, followed by cobalt impregnation and high‐temperature pyrolysis that generates Co–carbon interfaces with strong coupling between Co and dope carbon, thereby forming a binder‐free, membrane‐type structure [101]. The optimized CoNOC//CFC‐900 exhibited excellent HER activity in 0.5 M H_2_SO_4_, delivering a *J*_HER_ of −80 mA/cm at −0.15 V, η_10_ of 69 mV, and a Tafel slope of 46 mV/dec, indicating favorable Volmer–Heyrovsky kinetics. It showed negligible decay in polarization curves after 6000 cycles. It maintained a stable *J*_HER_ over 8 h due to the encapsulated Co NPs and carbon framework preventing aggregation and catalyst degradation during long‐term operation. The HER performance was ascribed to the synergistic interaction between cobalt nanocrystals and heteroatom‐rich carbon matrices, where N, O‐dual doping and Co encapsulation reduced the hydrogen adsorption free energy on adjacent carbon sites, enhanced electronic conductivity, and increased electrochemically active surface area (ECSA). However, the multistep reaction is a major barrier [101].

Two‐dimensional cobalt selenide nanosheets vertically grown on 3D carbon fibers (CoSe_2_/CF) were fabricated via electrodeposition of a Co(OH)F precursor on carbon fibers, followed by hydrothermal anion‐exchange selenization, thereby forming a binder‐free material with a preserved hierarchical structure, with direct CoSe_2_/carbon fiber electrical contact and a short electron path [102]. The CoSe_2_/CF showed good HER activity in 0.5 M H_2_SO_4_, displaying a *J*_HER_ of −250 mA/cm^2^ at −0.20 V, an η_10_ of 118 mV, and a Tafel slope of 53.2 mV/dec, indicating that the reaction followed the Volmer–Heyrovsky pathway. CoSe_2_/CF exhibited stability of over 12 h in chronoamperometric testing, exhibiting unchanged polarization characteristics after 1000 continuous cycles, where the strong nanosheet–carbon fiber interaction prevents structural collapse and maintains active site exposure. The HER performance was attributed to the intrinsic metallic conductivity of CoSe_2_, the vertically aligned nanosheet structure providing abundant exposed active sites, and the highly conductive carbon fiber substrate facilitating rapid electron transport [102]. However, due to high *J*_HER_, CoSe_2_/CF activity remains sensitive to overpotential, meaning that further changes to the electronic structure are needed to reduce energy losses under conditions important for industry.

Phosphine‐reduced iron‐doped tungsten oxide nanoplates supported on reduced graphene oxide (Fe‐WO_x_P/rGO) were synthesized via the hydrothermal growth of Fe‐WO_3_ on rGO followed by in situ phosphine reduction, generating defect‐rich Fe‐WO_x_P/rGO interfaces that enhance electrical coupling and active site exposure [103]. The Fe‐WO_x_P/rGO exhibited excellent HER activity in 0.5 M H_2_SO_4_, delivering a *J*_HER_ of −100 mA/cm^2^ at −0.17 V, η_10_ of 54.6 mV, and a Tafel slope of 41.99 mV/dec, indicating rapid Volmer–Heyrovsky kinetics with a fast hydrogen intermediate. It maintained stability for 24 h due to the strong Fe‐WO_x_P/rGO interaction, which suppresses structural degradation and supports continuous charge transport. The HER performance resulted from the synergistic integration of Fe doping and phosphorus incorporation, which created numerous oxygen vacancies in the WO_x_ lattice, enhanced electronic conductivity, and facilitated rapid charge transfer through the rGO support, as validated by XPS and EIS analyses. However, the uniformity of rGO dispersion may limit long‐term stability and scalability under practical operating conditions [103].

Defect‐rich amorphous MoS_x_ nanosheets, horizontally grown on carbon nanofiber arrays supported on carbon cloth (MoS_x_/CF/CC‐2), were synthesized by electrochemical polymerization of polypyrrole on carbon cloth, followed by carbonization to form carbon nanofiber arrays and subsequent hydrothermal growth of amorphous MoS_x_ using ammonium molybdate and thioacetamide at 200°C, producing a hierarchical MoS_x_/carbon interface with abundant defect sites and efficient electron pathways [104]. SEM imaging of the prepared MoS_x_/CF/CC‐2 (Figure 3a) shows that densely packed MoS_x_ nanosheets uniformly coat the carbon nanofiber scaffold, forming a highly porous and interconnected architecture. HRTEM analysis (Figure 3b) reveals structural defects and an increased interlayer spacing of 0.66 nm corresponding to the (002) plane. This is expected to increase the density of catalytically active sulfur sites and facilitate electrolyte penetration. Electrochemical testing in 0.5 M H_2_SO_4_ shows that MoS_x_/CF/CC‐2 has better HER activity than MoS_x_/CC and other control samples. It has a much lower overpotential and a higher *J*_HER_ across the potential range that was examined (Figure 3c). The MoS_x_/CF/CC‐2 exhibits a high *J*_HER_ of −500 mA/cm^2^ at −0.26 V, η_10_ of 127 mV, and a Tafel slope of 51 mV/dec, significantly lower than those of MoS_x_/CF/CC‐1 and MoS_x_/CF/CC‐3, indicating faster HER kinetics (Figure 3d). Polarization curves taken before and after 1000 CV cycles show almost the same durability (Figure 3e). SEM analysis after stability indicates that the hierarchical morphology continues to exist (Figure 3f), demonstrating good electrochemical robustness [104]. However, a multistep reaction is a great barrier.

**FIGURE 3:**
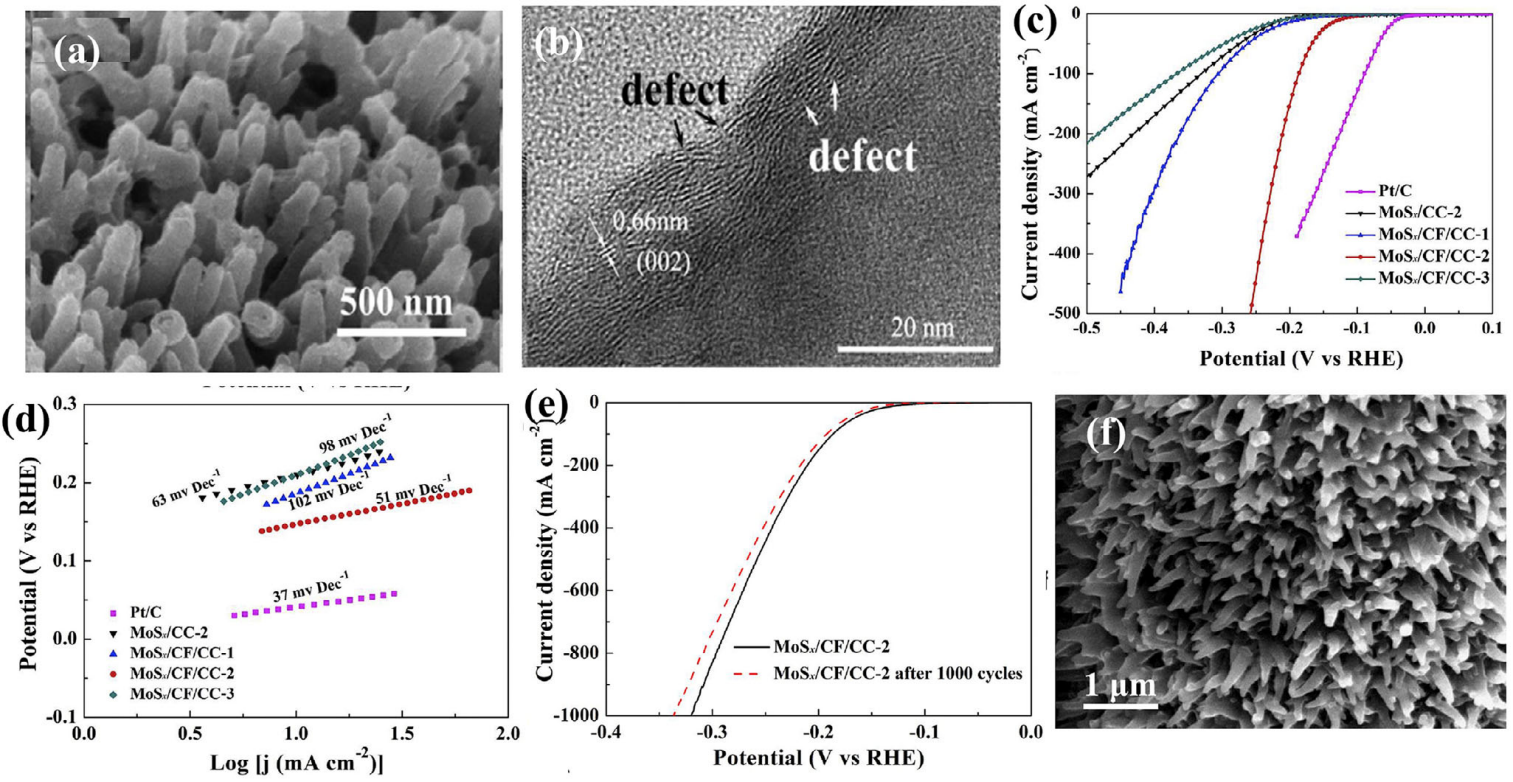
(a) SEM image, (b) TEM image of MoS_x_/CF/CC‐2, (c) LSV, (d) Tafel slope of MoS_x_/CF/CC‐2 compared with others, (e) LSV before and after 1000 CV cycles of MoS_x_/CF/CC‐2, (f) SEM image of MoS_x_/CF/CC‐2 after the stability test in 0.5 M H_2_SO_4_. Copyright 2018 Elsevier, Ref. [104].

Ni/NiO/TiO_2_/rGO nanocomposites directly grown on carbon cloth (Ni/NiO/TiO_2_/rGO@CC) were fabricated via a one‐pot plasma‐enhanced chemical vapor deposition (PECVD) process, directly integrating multicomponent interfaces and creating strong hierarchical Ni/NiO/TiO_2_/rGO coupling for efficient electron transfer in a binder‐free structure [105]. The optimized NGT exhibited excellent HER activity in 0.5 M H_2_SO_4_, delivering a *J*_HER_ of −10 mA/cm^2^ at −0.15 V, a η_10_ of 130 mV, and a Tafel slope of 40 mV/dec, indicating favorable Volmer–Heyrovsky kinetics. It maintained its stability for 60 h. The stable heterointerface and robust carbon cloth anchoring suppress catalyst degradation during prolonged operation, with improved HER activity due to the combined roles of metallic Ni, p–n NiO/TiO_2_ heterojunctions, and conductive rGO layers, and featured charge separation, faster electron transport, and a larger ECSA, as evidenced by EIS results and double‐layer capacitance measurements. However, making multicomponent heterostructures in a complicated way may make it harder to scale up and limit their use [105].

MoS_2_ nanosheets attached to multi‐walled carbon nanotubes (MWCNTs/MoS_2_) were synthesized via a hydrothermal method and then coated onto carbon cloth to form a composite electrocatalyst [106]. The hybrid MWCNTs/MoS_2_ creates conductive pathways and improves interfacial electrical contact with carbon cloth, which is reflected in HER activity. In 0.5 M H_2_SO_4_, displaying a *J*_100_ at −0.55 V, η_10_ of 211 mV, and a Tafel slope of 136 mV/dec. MWCNTs/MoS_2_ maintained stability for 22 h, as the flexible carbon network preserves catalyst dispersion and prevents structural aggregation during electrolysis. The better HER was due to the combination of MoS_2_ edge‐rich nanosheets and the highly conductive MWCNT network, which increased the ECSA, improved charge transport, and lowered charge‐transfer resistance to 3.0 Ω, as shown by *C*_dl_, ECSA; where the porous carbon cloth framework promotes uniform catalyst dispersion and exposes a greater number of accessible active sites through efficient interfacial integration. However, the relatively high Tafel slope and the large overpotential required to reach high current densities indicate limited intrinsic HER kinetics on MoS_2_ basal planes, necessitating further electronic or defect engineering for high‐efficiency applications [106].

A self‐supported MoS_2_/FeS_2_ nanocomposite, directly synthesized on carbon cloth (MoS_2_/FeS_2_/CC), was created using a single‐step chemical vapor deposition (CVD) method. This process resulted in vertically aligned MoS_2_ nanosheet arrays that uniformly covered the FeS_2_ NPs heterointerface, enabling rapid electron transport and strong catalyst adhesion [107]. The optimized MoS_2_/FeS_2_/CC exhibited HER activity in 0.5 M H_2_SO_4_, delivering a *J*_HER_ of −100 mA/cm^2^ at −0.20 V, η_10_ of 134 mV, and a Tafel slope of ∼78.6 mV/dec, indicative of Volmer–Heyrovsky kinetics with electrochemical desorption as a contributing step. It maintained stability for 24 h, keeping stable HER performance over 2000 CV cycles; the self‐supported architecture and strong interface prevented catalyst detachment. The enhanced HER stemmed from the synergistic interaction between MoS_2_ nanosheets and FeS_2_ NPs. This interaction resulted in greater accelerated charge transfer, exposure of active sites, and improved electrical conductivity, facilitated by the self‐supported three‐dimensional structure, as demonstrated by ECSA and EIS [107]. However, the HER activity remains inferior to that of noble‐metal catalysts. This is due to the inherent kinetic constraints of MoS_2_ basal planes, despite the inclusion of FeS_2_.

Mesoporous iron phosphide films grown directly on carbon cloth (meso‐FeP film/CC) were synthesized via a two‐step process involving evaporation‐induced self‐assembly (EISA) of an iron hydroxide/F127 precursor followed by calcination and phosphidation, providing interconnected FeP networks with short electron paths for electrochemical HER [108], as illustrated in Figure 4a. TEM and elemental mapping (Figure 4b) confirm the formation of a highly porous FeP film with uniform Fe and P distribution. Electrochemical HER in 0.5 M H_2_SO_4_ shows that the meso‐FeP film/CC is more active than the FeP NWs/CC, FeP/CC, and unsupported meso‐FeP. It has an η_10_ of 65 mV at 10 mA/cm^2^ (Figure 4c), a *J*_HER_ of 100 mA/cm^2^ at −0.12 V, and a Tafel slope of 44 mV/dec, indicating a Volmer–Heyrovsky mechanism. Nyquist plots (Figure 4d) show that the meso‐FeP film/CC electrode has a smaller semicircle diameter than those of FeP NWs/CC, FeP/CC, and meso‐FeP, indicating lower charge‐transfer resistance. The analysis of double‐layer capacitance, derived from linear fitting of capacitive currents (Figure 4e), reveals a larger ECSA for the meso‐FeP film/CC, consistent with its ordered mesoporous structure and interconnected pores that improve electrolyte penetration and increase accessible FeP active sites. The meso‐FeP film/CC electrode exhibits remarkable durability, maintaining 90.8% of its initial *J*_HER_ after 20 h of continuous operation and displaying minimal degradation after 1000 CV cycles, owing to its robust mesoporous FeP framework and strong substrate interaction [108]. However, the multistep EISA–phosphidation process is a significant concern.

**FIGURE 4:**
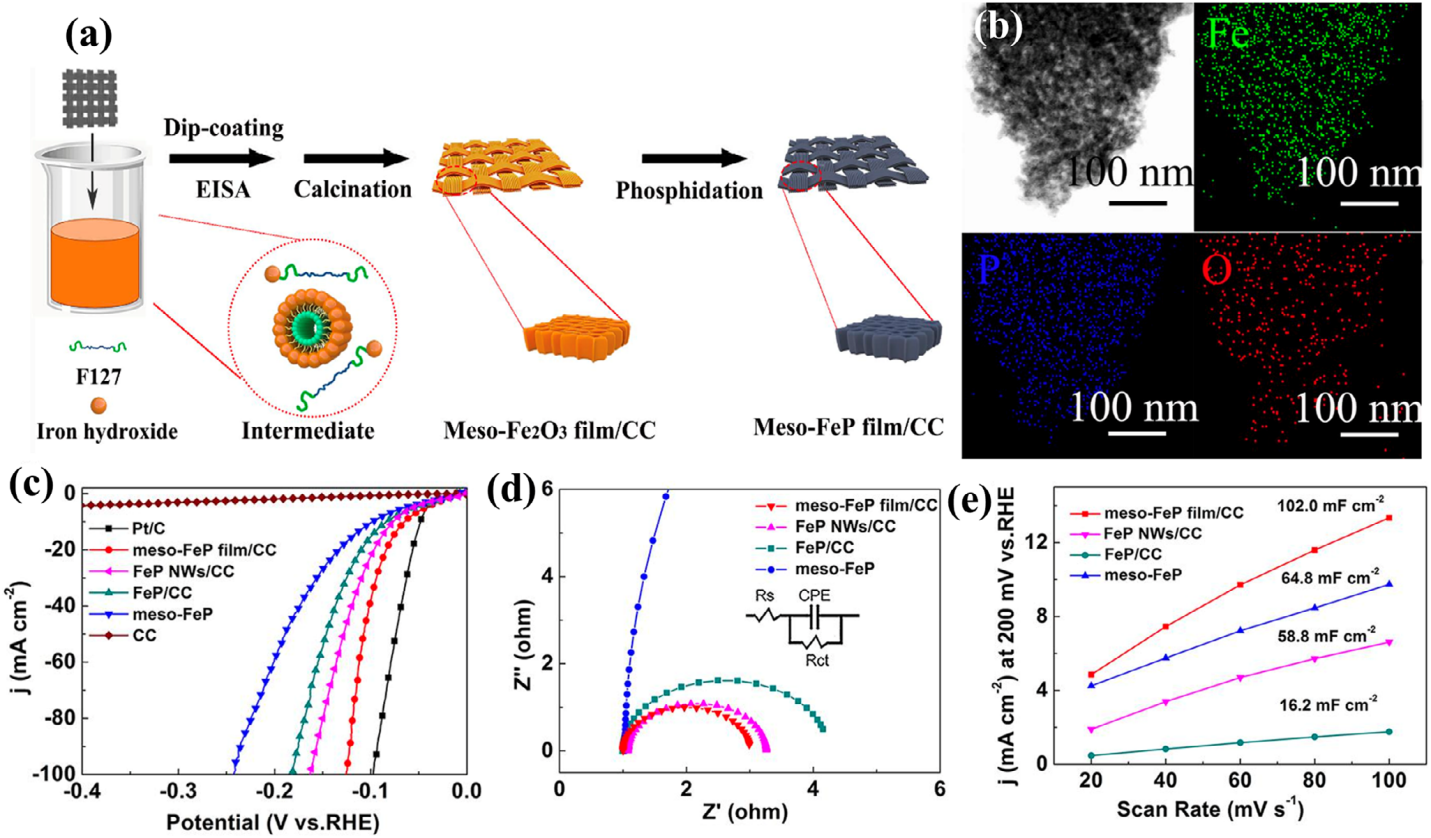
(a) Schematic design for the fabrication of meso‐FeP film/CC, (b) TEM‐EDS mapping of Fe, P, and O elements, (c) LSV, (d) Nyquist plots, (e) Linear fitting by plotting the capacitive currents at 0.2 V Vs scan rates, for meso‐FeP film/CC compared with others in 0.5 M H_2_SO_4_. Copyright 2020 WILEY‐VCH, Ref. [108].

A vertically aligned tungsten (W)‐doped vanadium diselenide (VSe_2_) nanoplate array was synthesized on carbon cloth (CC) (W_x_V_1__−__x_Se_2_/CC) using hydrothermal methods and thermal annealing, resulting in a three‐dimensional, direct‐growth‐enabled, binder‐free structure with efficient electron transport and stable catalyst anchoring [109]. In 0.5 M H_2_SO_4_, the optimized W_x_V_1__−__x_Se_2_/CC showed increased HER activity, with a *J*_HER_ of −30 mA/cm^2^ at −0.20 V, η_10_ of 173 mV, and a Tafel slope of ∼80 mV/dec, indicating a Volmer‐Heyrovsky reaction pathway. The W_x_V_1__−__x_Se_2_/CC maintained effective HER activity after 1000 CV cycles, as the stable nanoplates architecture prevents active material loss during cycling. Electrical conductivity, charge‐transfer resistance, and the number of catalytically active sites increased, thereby enhancing HER performance upon W doping, as evidenced by higher double‐layer capacitance and EIS. Doping induces electronic modulation and defect formation, improving active site exposure and charge transfer. Although VSe_2_‐based active sites have higher activity than pristine VSe_2_, their inherent kinetic restrictions require further electronic‐structure optimization [109].

The hierarchical, binder‐free structure of phosphorus‐doped MoSe_2_/MoS_2_ heterojunction nanosheets was synthesized via hydrothermal growth and controlled phosphorization, generating MoSe_2_/MoS_2_ interfaces with P‐induced defects that enhance electronic coupling and active site density [110]. The optimized P‐MoSe_0.5_S_1.5_/N‐CNTs/CC‐2 demonstrated HER activity in 0.5 M H_2_SO_4_, with a *J*_HER_ of −60 mA/cm^2^ at −0.16 V, η_10_ of 108.3 mV, and a Tafel slope of 58.6 mV/dec, indicating favorable Volmer‐Heyrovsky reactions. The P‐Mo Se_0.5_S_1.5_/N‐CNTs/CC‐2 exhibited remarkable stability over a 12‐h continuous operation period at 40 mA/cm^2^; the *J*_HER_ remained largely unchanged, with only a minor reduction in performance, which means it maintained structural integrity during electrolysis. The observed HER performance was attributable to the MoSe_2_/MoS_2_ heterojunction interfaces, phosphorus defects, and the conductive N‐CNT scaffold that optimizes hydrogen adsorption energy and accelerates electron transfer. The ECSA, charge transfer, and inherent catalytic activity exhibited an increase in EIS and TOF values. But multistep reactions pose major problems [110].

A simple two‐step dip‐coating and electrochemical reduction procedure produced self‐supported, binder‐free molybdenum oxide–stabilized ultrafine palladium NPs directly supported on carbon cloth (MoO_x_–Pd/CC) that promotes electronic interaction between oxide and metal phases [111]. The optimized MoO_x_–Pd/CC demonstrated remarkable HER performance in 0.5 M H_2_SO_4_, with a *J*_HER_ of −200 mA/cm^2^ at −0.10 V and a η_10_ of 25 mV. A Tafel slope of 36.9 mV/dec reflects fast hydrogen desorption kinetics after proton adsorption following a Heyrovsky step. The MoO_x_–Pd/CC maintained HER activity for at least 7 days and only showed a slight increase in overpotential, where the strong interface prevents NPs aggregation and maintains active surface exposure during prolonged electrolysis. The activity was due to the stability of ultrafine Pd NPs (1–4 nm) induced by MoO_x_, the density of oxygen vacancies, and the strong interactions between the metal and oxide. According to EIS, ECSA, and DFT studies [117], these changes to Pd's electronic structure increased the active site density and decreased the charge‐transfer resistance to 0.56 Ω. Despite the remarkable activity, palladium's cost relative to fully earth‐abundant HER catalysts could limit large‐scale development.

A hybrid electrocatalyst, combining molecular and carbon components, was created by drop‐casting molecular 2‐thioureidobenzothiazole palladium complexes onto graphitized carbon cloth (Pd(II)BTT/GrCC) [112]. The Pd(II)BTT/GrCC demonstrated HER activity in 0.5 M H_2_SO_4_, with a *J*_HER_ of −10 mA/cm^2^ at −0.20 V, η_10_ of 419 mV, and a Tafel slope of ∼144 mV/dec. This suggests a Volmer–Heyrovsky pathway controlling HER kinetics. The Pd(II)BTT/GrCC maintained its stability for 6 h. The graphitized carbon support improves mechanical stability and maintains catalyst distribution during operation. The improved performance of graphitized carbon cloth compared to unmodified Pd(II)BTT is due to its porous structure and its inherent ability to conduct electricity. These characteristics facilitate an increase in the ECSA, thereby promoting more efficient charge‐transfer processes. Double‐layer capacitance and impedance measurements proved this [112]. However, the high overpotential and large Tafel slope suggest that the immobilized molecular sites have limited inherent catalytic activity.

Three‐dimensional net‐like Ni_3_(VO_4_)_2_ ultrathin nanosheets coupled with MoS_2_ grown on carbon fiber cloth (Ni_3_(VO_4_)_2_/MoS_2_@CFC) were synthesized via a two‐step hydrothermal strategy, in which MoS_2_ nanosheets were first grown on CFC, followed by secondary growth of Ni_3_(VO_4_)_2_ to form an interpenetrated heterostructure [113]. The sequential growth creates an intimate Ni_3_(VO_4_)_2_/MoS_2_ heterointerface with strong coupling to the carbon fiber substrate. A highly open and interconnected architecture is formed by vertically intersecting ultrathin Ni_3_(VO_4_)_2_ nanosheets with exposed MoS_2_ regions, as shown in SEM images (Figure 5a,b). The uniform distribution of Ni, V, Mo, S, and O is confirmed by elemental mapping, suggesting close interfacial coupling between Ni_3_(VO_4_)_2_ and MoS_2_ (Figure 5c). In 0.5 M H_2_SO_4_, electrochemical analysis reveals that the optimized Ni_3_(VO_4_)_2_/MoS_2_@CFC displayed a η_10_ of 77 mV at 10 mA/cm^2^, a Tafel slope of 65 mV/dec indicates Volmer–Heyrovsky kinetics, and a higher *J*_HER_ of −150 mA/cm^2^ at −0.30 V. It is also very durable, with a stable HER performance for 12 h at a *J*_HER_ of 10 mA/cm^2^. The interconnected heterostructure maintains active site accessibility and prevents structural degradation. Across the analyzed potential range, the CFC electrode exhibits better HER activity than the Ni/V/MoS_2_@CFC variants and the individual components (Figure 5d). At higher temperatures, catalytic activity improves even more, and the overpotential decreases between 25 and 75°C (Figure 5e). When contemplating the capacitive current, we observe that Ni_3_(VO_4_)_2_/MoS_2_@CFC has a larger ECSA than the control samples originates from the open nanosheet network(Figure 5f). Furthermore, the feasibility of solar‐driven hydrogen production using the Ni_3_(VO_4_)_2_/MoS_2_@CFC electrode is illustrated schematically in Figure 5g. This indicates that the 3D heterointerface architecture is robust. Although Ni_3_(VO_4_)_2_/MoS_2_@CFC is more active and stable, its HER performance is still not as good as that of Pt‐based catalysts at low overpotentials. The multicomponent heterostructure may also make it harder to control the composition [113].

**FIGURE 5:**
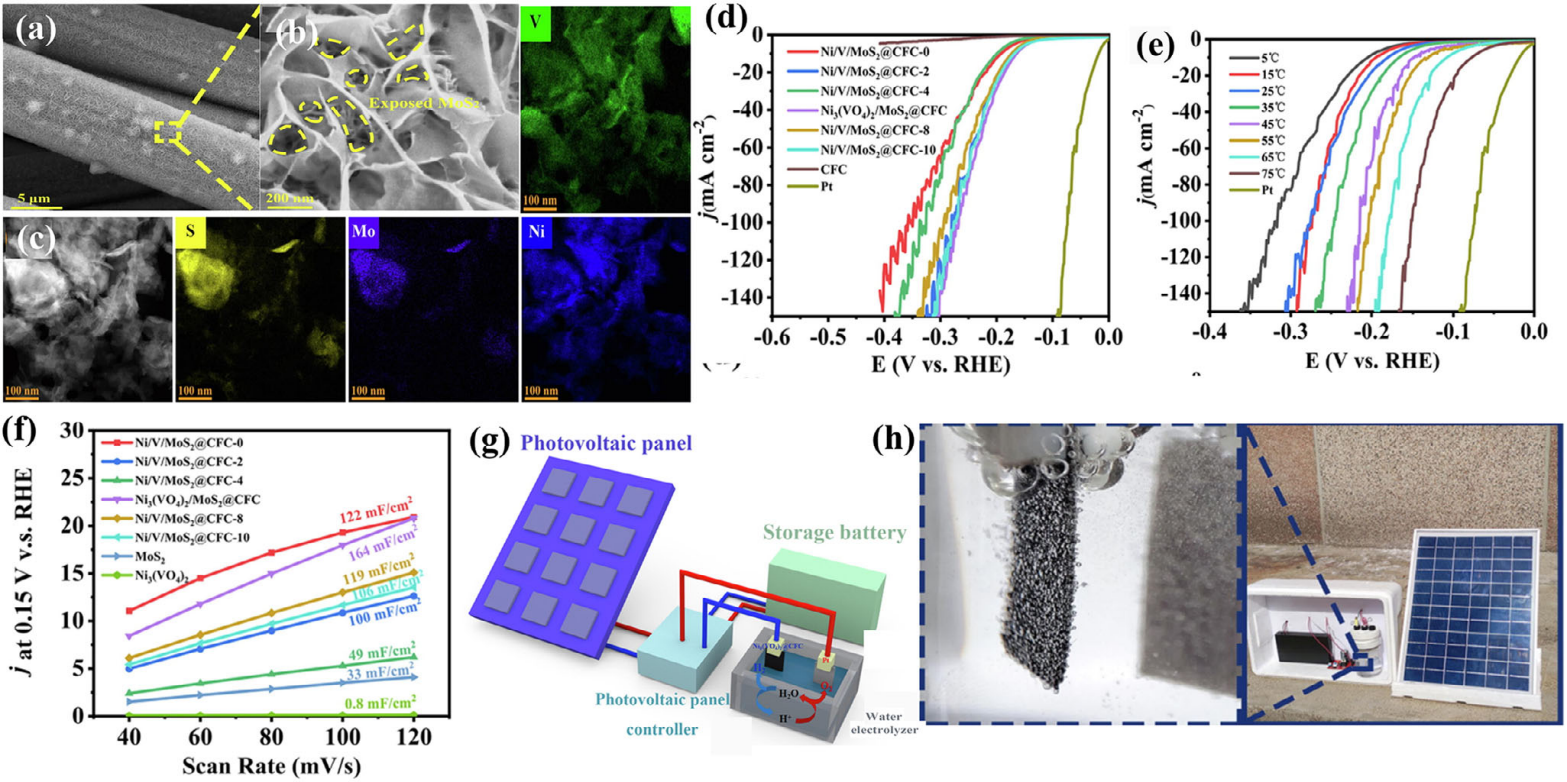
SEM images of (a) Ni_3_(VO_4_)_2_/MoS_2_@CFC and (b) MoS_2_ nanosheets. (c) Elemental mapping images of S, Mo, N, and V. (d) LSV with different Ni and V ratios, (e) LSV of Ni_3_(VO_4_)_2_/MoS_2_@CFC at different temperatures, (f) Plots of the capacitive currents as a function of scan rate, (g) Schematic diagram, (h) and Digital photograph in 0.5 M H_2_SO_4._ Copyright 2022 Elsevier, Ref. [113].

The flower‐like Ni/Fe‐MoS_2_/CC, created by directly growing nickel‐ and iron‐codoped molybdenum disulfide on carbon cloth, was synthesized via the hydrothermal method. This process produces a three‐dimensional structure with multiple interfaces and a mixture of 1T and 2H MoS_2_ phases [114]. Ni/Fe‐MoS_2_/CC demonstrated excellent HER performance in 0.5 M H_2_SO_4_, with a *J*_HER_ of −20 mA/cm^2^ at −0.15 V, η_10_ of 116 mV, and a Tafel slope of 43 mV/dec, indicating favorable Heyrovsky kinetics with fast electrochemical desorption. The Ni/Fe‐MoS_2_/CC demonstrated durability for over 15 h and no deterioration after 5000 CV cycles, as strong MoS_2_–CC interaction prevents catalyst detachment while maintaining structural integrity. Co‐doping with Ni and Fe led to a partial phase change from semiconducting 2H‐MoS_2_ to metallic 1T‐MoS_2_, greater sulfur vacancy concentration, and reduced charge‐transfer resistance (Rct ≈ 2.9 Ω), resulting in enhanced HER activity, as demonstrated by Raman, XPS, and EIS tests. The dopants modulate electronic structure and H adsorption energy. Despite its improved HER performance, the Ni/Fe‐MoS_2_/CC still exhibits lower activity than Pt‐based catalysts, thereby limiting its immediate industrial applicability [114].

Ru‐doped tungsten nitride nanosheet arrays, directly synthesized on carbon cloth (Ru‐WN/CC), were prepared via hydrothermal growth of Ru‐WO_3_ on carbon cloth, followed by high‐temperature in situ nitridation in an NH_3_ environment [115]. The optimized 7% Ru‐WN/CC exhibited remarkable HER performance in 0.5 M H_2_SO_4_, achieving a *J*_HER_ of ‐100 mA/cm^2^ at ‐0.15 V, a η_10_ of 94 mV, and a Tafel slope of 51.4 mV/dec, thereby suggesting a Volmer–Heyrovsky mechanism. Ru‐WN/CC is stable, maintaining the *J*_HER_ almost constant during 24 h of continuous electrolysis at a fixed overpotential. Ru doping altered the electronic structure of WN, reduced the charge transfer resistance (Rct ≈ 6.08 Ω), and indicated enhanced electronic coupling at the catalyst–carbon cloth interface, facilitating faster electron injection from the substrate to the catalytically active sites and thereby accelerating HER kinetics. ECSA was also increased by creating highly porous nanosheet arrays. The following was verified by XPS, EIS, and ECSA analyses. However, excessive Ru doping was found to deteriorate HER performance, indicating a narrow optimal dopant window and potential scalability challenges associated with noble‐metal utilization [115].

Flake‐like structured PANI‐pf‐GCC@MoS_2_ was synthesized via a hydrothermal process by uniformly decorating a porous graphene–carbon cloth framework with MoS_2_ flowers and a polyaniline (PANI) network [116]. SEM images (Figure 6a,b) show a homogeneous distribution of MoS_2_ with a flower‐like morphology composed of wrinkled nanosheets, providing abundant exposed edge sites. XPS analysis (Figure 6c) shows that Mo^4+^ and Mo^6+^ species exist at the same time as S 2s signals. This means that MoS_2_ is partially oxidized, which can change its electronic structure. HER testing in 0.5 M H_2_SO_4_ shows that PANI‐pf‐GCC@MoS_2_ exhibits better HER activity than CC, CC@MoS_2_, and PANI‐pf‐GCC. It has a η_10_ of 178 mV and a higher *J*_HER_ of −70 mA/cm^2^ at ‐0.5 V (Figure 6d). The corresponding Tafel plots (Figure 6e) show a markedly smaller slope (≈40 mV/dec) for PANI‐pf‐GCC@MoS_2_ than for CC@MoS_2_ (≈80 mV/dec) and PANI‐pf‐GCC (≈325 mV/dec). The lower slope confirms faster HER kinetics due to improved interfacial charge transfer. A further comparison (Figure 6f) shows that adding Ni (PANI‐pf‐GCC@Ni‐MoS_2_) lowers the overpotential by 159 mV compared to PANI‐pf‐GCC@MoS_2_. This demonstrates the usefulness of heteroatom modulation. Chronoamperometry stability tests show that PANI‐pf‐GCC@MoS_2_ maintains its HER performance with moderate stability over a 60‐h electrolysis duration at a constant potential. The conductive PANI/graphene framework enhances mechanical adhesion and electron transport. However, overall HER activity and durability remain inferior to noble‐metal benchmarks. Also, partial oxidation of Mo species may make it more difficult to achieve long‐term stability under acidic conditions [116].

**FIGURE 6:**
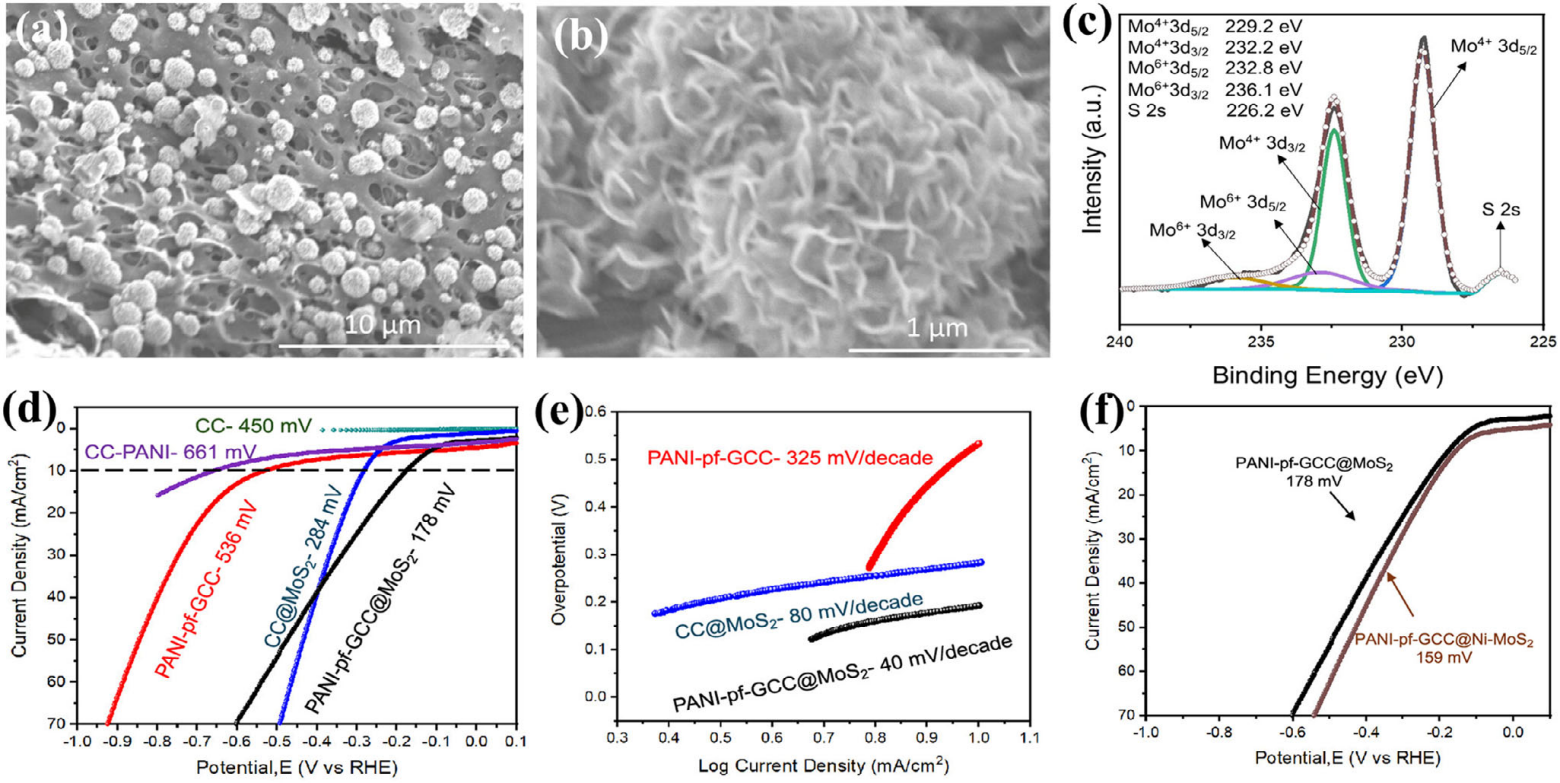
(a) SEM images of PANI‐pf‐GCC@MoS_2_ showing the homogeneous distribution of MoS_2_, (b) Zoom‐in SEM image of the MoS_2_ flower, (c) High‐resolution Mo 3d XPS spectra, (d) LSV, (e) Tafel plots, (f) of PANI‐pf‐GCC@MoS_2,_ HER performance comparison between PANI‐pf‐GCC@ MoS_2_ and PANI‐pf‐GCC@Ni‐MoS_2_ samples in 0.5 M H_2_SO_4._ Copyright 2024 ACS, Ref. [116].

The porous three‐layered heterostructure of a bimetallic nickel–molybdenum selenide composite supported on TI_3_C_2_TX MXene‐coated carbon cloth (NiMoSe/Ti_3_C_2_T_x_@CC) fabricated via a hydrothermal method enhanced the HER activity more than NiSe@CC and MoSe@CC with a *J*_HER_ of −50 mA/cm^2^ at −0.57 V, a η_10_ of 403 mV, and a Tafel slope of 33 mV/dec in 0.5 M H_2_SO_4_, besides stability for 36 h and 1000 cycles [117]. This is due to the synergistic Ni–Mo electronic interactions, the high conductivity and hydrophilicity of Ti_3_C_2_T_x_, and the porous carbon cloth substrate. The activity is close to that of the Pt/C catalyst, but the effect of the Mxene and metal active sites is not clear.

### 7.1 Critical Analysis of Interfacial Design Principles in Acidic HER

To summarize, the studies discussed above demonstrate that carbon cloth serves a role beyond that of a conductive current collector. Direct catalyst growth establishes intimate interfacial contact, minimizes electrical resistance, and promotes rapid electron transport to the active sites. Meanwhile, the three‐dimensional porous architecture enhances catalyst dispersion, electrolyte penetration, hydrogen bubble release, and electrode stability. Furthermore, heteroatom doping, heterostructure construction, and defect engineering strengthen catalyst–carbon cloth electronic coupling, reducing charge‐transfer resistance and improving HER kinetics. Overall, the reported studies indicate that interface engineering, rather than catalyst composition alone, plays a decisive role in HER performance. However, quantitative structure–activity relationships linking interfacial chemistry to catalytic activity remain limited and require further investigation through operando characterization and theoretical studies.

## 8 Interfacial Interaction of CC with Metals/Nonmetals in KOH

As discussed in the section on acidic media, the interfacial interaction between carbon cloth and various metal/nonmetal catalysts plays a pivotal role in enhancing HER performance. The same fundamental principles apply in alkaline media. In Table 4, a summary of the activity of various catalysts in KOH for carbon cloth electrode substrates is provided.

**TABLE 4:** Comparison of the HER performance of CC‐supported metals/nonmetals in KOH.

Catalyst	Preparation method	η_10_ (mV)	TAFEL (mV/dec)	Current density	Stability (h/%)	References
Co‐P/NC/CC	Impregnation, annealing at 900°C, and phosphidation	−171	52	∼−100 mA/cm^2^ @ −0.25 V	20 h	[118]
Co_2_B/CoSe_2_	In situ chemical reduction–deposition	300	76	∼−50 mA/cm^2^ @ −0.38 V	30 h	[121]
P–CoMoS/CC	Hydrothermal and phosphorization	66 mV	60.1	∼−150 mA/cm^2^ @ −0.32 V	100 h	[124]
Ni_0_._33_Co_0_._67_MoS_4_/CFC	Hydrothermal reaction and vulcanization	48	73.5	∼−100 mA/cm^2^ @ −0.23 V	10 h	[125]
Co_0_._93_Ni_0_._07_P_3_	Hydrothermal growth and phosphidation	87	60.7	∼−100 mA/cm^2^ @ −0.15 V	10 h	[126]
Co–Mo–S(1:2)/CC	Hydrothermal method	92	82	∼−300 mA/cm^2^ @ −0.25 V	12 h	[127]
Fe–MoS_2_/Ni_3_S_2_/NF‐2	Solvothermal method	130.6	112.7	∼−200 mA/cm^2^ @ −0.3 V	20 h	[128]
EACC‐Co‐500	Electrodeposition followed by electrochemical activation	195	142	∼−25 mA/cm^2^ @ −0.25 V	4.17 h	[129]
MoS_2_@CoO/CC	Hydrothermal method, sonication, and annealing	173	129.9	∼−160 mA/cm^2^ @ −0.4 V	10 h	[130]
Mo‐Co‐S‐Se/CC	Solvothermal growth	58	84	∼−70 mA/cm^2^ @ −0.17 V	11 h	[131]
NiS–MoS_2_/CC	Hydrothermal process	106	56.7	∼−100 mA/cm^2^ @ −0.18 V	24 h	[132]
N‐doped CoP_2_@PCC	Thermal treatment and acid etching, followed by hydrothermal growth	64	47.4	∼−200 mA cm^−2^ @ −0.14 V	15 h	[133]
Mn‐O@Co‐P/CC	Electrodeposition–phosphorization technique	106	56	∼−150 mA/cm^2^ @ −0.2 V	12 h	[134]
Co(OH)_2_/MoS_2_/CC	Hydrothermal method and electrodeposition process	101	61.65	∼−200 mA/cm^2^ @ −0.25 V	12 h	[135]
NAC@NiCo_2_S_4_ NWs/CC	Hydrothermal method and annealing	254 @ 100 mA/cm^2^	57	∼−300 mA/cm^2^ @ −0.3 V	20 h	[136]
Ni/Ni(OH)_2_/NiOOH/CC	Electrodeposition technique	45	99.6	∼−200 mA/cm^2^ @−0.37 V	200 h	[137]
NiMo/Ni(OH)_2_/CC	Hydrothermal method and electrodeposition technique	132	134.1	∼−80 mA/cm^2^ @ −0.3 V	24 h	[138]
CoSe_2_/MoSe_2_/CC	Impregnation, autoclave kept at 150°C for 6 h followed by annealing and selenization	90	84.8	∼−80 mA/cm^2^ @ −0.2 V	12 h	[139]
NW‐MnCo_2_O_4_/GDY	Hydrothermal growth	180	65	∼ −500 mA/cm^2^ @ 0.7 V	40 h	[140]
Se‐NiCoP/CC‐2	Hydrothermal reaction and phosphorization/selenization	79	81.6	∼−350 mA/cm^2^ @ −0.2	20 h	[141]
Sn‐CoS_2_/CC	Hydrothermal and sulfuration	161	94	∼−100 mA/cm^2^ @ −0.3 V	32 h	[142]
1T‐Fe/P‐WS_2_@CC	Hydrothermal reaction	116	65	∼−150 mA/cm^2^ @ −0.25 V	24 h	[143]
Co_9_S_8_‐V_3_S_4_/CC	Hydrothermal treatment and sulfurization	85	51	∼−200 mA/cm^2^ @ −0.3 V	40 h	[144]
Zn_x_Ni_1‐x_S@Mo_2_C/CC	Hydrothermal method	230	189	∼−100 mA/cm^2^ @ −0.4 V	—	[145]
Fe–Co–S/CC‐75	Hydrothermal method and annealing	320	82.6	∼−150 mA/cm^2^ @ −0.52 V	48 h	[146]
Co_3_O_4_/VO_2_/CC	Hydrothermal growth, electrodeposition, and annealing	108	98	∼−100 mA/cm^2^ @ −0.2 V	10 h	[147]
CoP_3_‐Nb_2_P/PCC	Hydrothermal reaction and phosphating	111	72.8	∼−500 mA/cm^2^ @ −0.3 V	50 h	[148]
Ru–CoP‐2.5 NAs	MOF‐derived method and phosphorization	52	39.7	∼−160 mA/cm^2^ @ −0.15 V	50 h	[149]
MoS_2_/Ru@CC	Hydrothermal method	71.3	104.8	∼−100 mA/cm^2^ @ −0.2 V	35 h	[150]
MoS_2_/CNTs/CC	Hydrothermal method	134	45.7	∼−105 mA/cm^2^ @ −0.22 V	10 h	[151]
CO_4_Ni_1_S/CC	Hydrothermal growth	192	119	∼−75 mA/cm^2^ @ −0.3V	36 h	[152]
AgCoPO_4_/CFP	Hydrothermal and phosphorization	32	34.4	∼−150 mA/cm^2^ @ −0.3V	25 h	[153]
CoNiSe‐CC	Electrodeposition technique and temperature selenization	180	63.1	∼−15 mA/cm^2^ @ −0.2 V	20 h	[154]
Fe–NiCo_2_Se_4_/CC	Hydrothermal route and selenization	148	85.7	∼−700 mA/cm^2^ @ −0.75 V	48 h	[155]
Fe_x_Mo_1__−__x_S_2_/CNT@CC	Hydrothermal method	116	44.7	∼−120 mA/cm^2^ @ −0.23 V	—	[156]
N‐Fe_7_S_8_/CC	Solvothermal synthesis, sulfurization, and nitridation	89	105	∼−40 mA/cm^2^ @ −0.15V	100 h	[157]
Co–Mo–S@MoS_2_/CC	Hydrothermal approach	43 at 20 mA/cm^2^	36.6	∼−130 mA/cm^2^ @ −0.3 V	51 h	[158]
UVO‐50 MoS_2_/CC	Hydrothermal synthesis UV‐Ozone (UVO) treatment	116	64	∼−100 mA/cm^2^ @ −0.23 V	—	[159]
FeP/FeO/CC	Hydrothermal method and phosphorization in argon	117	96.2	∼−300 mA/cm^2^ @ −0.3 V	20 h	[160]
CAO/NiS400/CC	Hydrothermal process and electrodeposition	25.5	20.4	∼−600 mA/cm^2^ @ −0.7 V	40 h	[161]
MoS_2_@CC‐H230	Hydrothermal synthesis	98 ± 3	108 ± 5	∼−100 mA/cm^2^ @ −0.22 V	15 h	[165]
NiO–MoS_2_/CC	Solvothermal method, drying, and calcination	159	116	∼−100 mA/cm^2^ @ −0.3 V	40 h	[166]
0.78MA0.32G@CC	Ultrasonic dispersion and drop‐casting	175.6	45.5	∼−400 mA/cm^2^ @ −0.72 V	30 h	[167]

Cobalt phosphide confined in N‐doped carbon nanopolyhedra was derived from ZIF‐67 grown on carbon cloth (Co‐P/NC/CC) by impregnation followed by annealing at 900°C for 3 h, then phosphidation at 300°C for 2 h [118]. Co‐P/NC/CC displayed HER *J*_HER_ of 90 mA/cm^2^ at −0.25 V with a Tafel slope of 52 mV/dec in KOH electrolyte, suggesting that the electrochemical desorption (Heyrovsky) step governs the overall HER kinetics. This activity was superior to those of Co/NC/CC, Zif‐67/CC, and CC, and outperformed CP/CTs/Co‐S [119] and CoO_x_@CN [120]. This is due to the porosity, high surface area, and electronic interactions of Co‐P, which exhibit high synergism relative to CC, thereby accelerating HER kinetics and enhancing charge mobility, as confirmed by EIS. However, the multiple reaction steps and high energy demand of ZIF‐67‐derived carbon are great barriers, along with inferior activity compared with self‐standing CoP materials [127], which makes the role of CC ambiguous.

Cobalt borate (Co_2_B) grew over CoSe_2_ nanosheets (Co_2_B/CoSe_2_) by the 30 mL autoclave method at 200°C for 12 h, then was frozen and lyophilized for 24 h, where amorphous Co_2_B was deposited in situ on the CoSe_2_ nanosheet surface, generating a well‐integrated heterointerface with strong interfacial contact. This interface promotes electronic coupling between Co_2_B and CoSe_2_ while preventing aggregation, resulting in uniform dispersion of active catalytic sites. The CoSe support provides rich nucleation sites for anchoring Co_2_B, leading to abundant active sites [121]. Co_2_B/CoSe_2_ had a hierarchical porous structure made of ultrathin nanosheets (Figure 7a,b). The porosity is crucial for promoting reactant adsorption and easing electrolyte diffusion. The elemental mapping analysis confirmed the co‐existence of Co, B, and Se with a ratio of 1.92:1 for Co‐B (Figure 7c). The LSV displayed a higher HER activity of Co_2_B/CoSe_2_ than those of Co_2_B and CoSe_2_/DETA, with an activity closer to Pt/C. The *J*_HER_ reaches −50 mA/cm^2^ at −0.23 V (Figure 7d), as well as a lower η_10_ of 300 mV and Tafel slope of 76 mV/dec in 1 M KOH electrolyte, suggesting a Volmer–Heyrovsky mechanism, where the electrochemical desorption (Heyrovsky) step is the rate‐determining process (Figure 7d). In addition, Co_2_B/CoSe_2_ demonstrated stability for a duration of 30 h, with a mere 1.9% activity loss (Figure 7e). The activity of Co_2_B/CoSe_2_ was superior to those of Co_2_B (328 mV) [122] and CoSe_2_ (520 mV) [123], due to the outstanding electrical conductivity of the CoSe support and the synergistic effect of Co_2_B along with porosity, which enhances diffusion of reactants and interaction with the electrolyte. The excellent durability is attributed to the robust interfacial bonding between Co_2_B and CoSe_2_, which suppresses catalyst detachment and structural degradation during prolonged electrolysis. Despite its unique porous morphology and support interactions, the HER current remains inferior, with a high η_10_ value that is far from that of Pt/C. This indicates that while the Co_2_B and CoSe_2_ demonstrate promising characteristics, there is still significant room for improvement in their performance. Future research may focus on optimizing the catalyst composition and structure to enhance the overall efficiency of the hydrogen evolution reaction.

**FIGURE 7:**
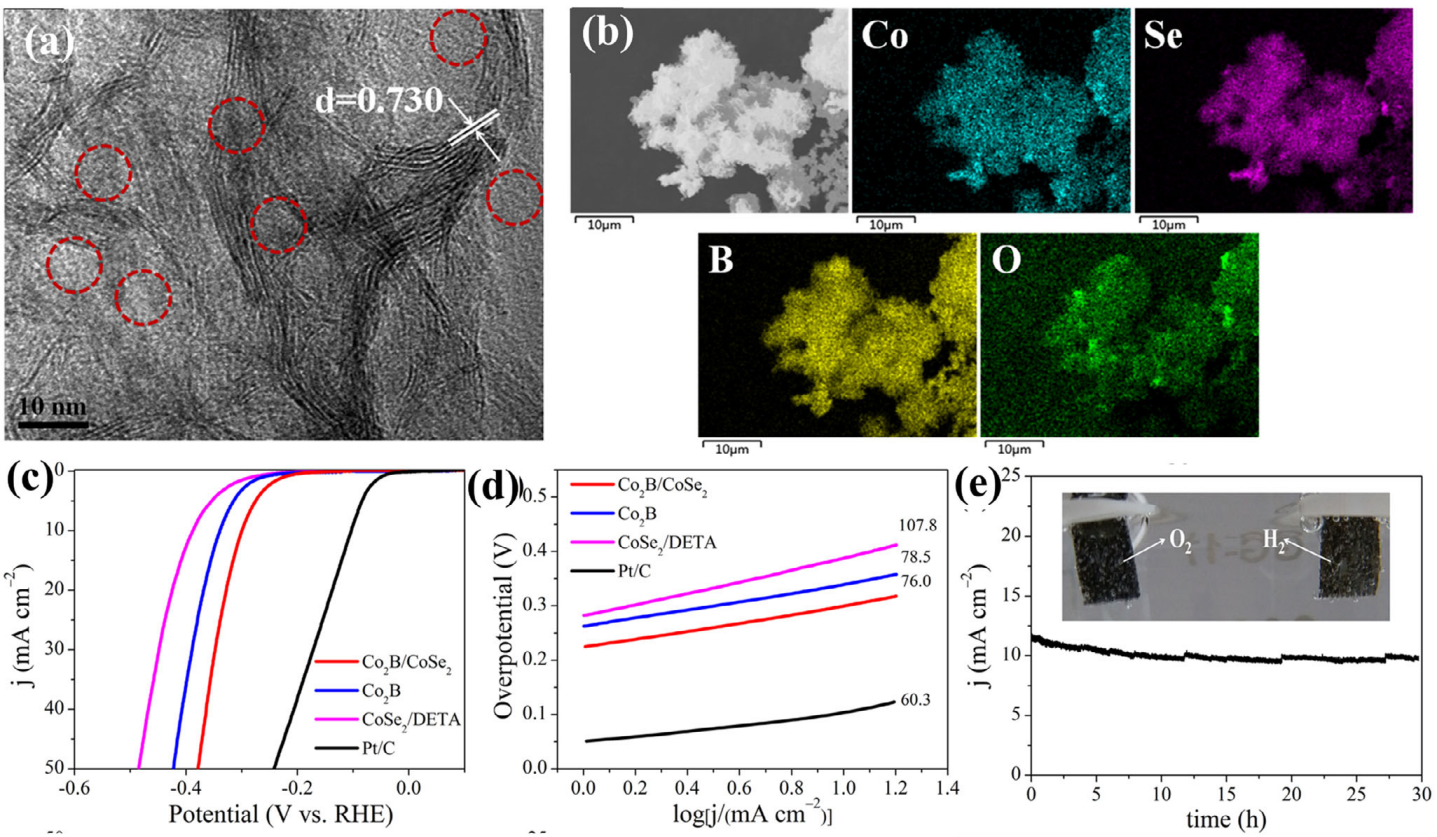
(a) HRTEM image of Co_2_B/CoSe_2_, (b) STEM and EDX elemental mapping images of Co_2_B/CoSe_2_, (c) LSV, (d) Tafel plots, and (e) Stability test of the electrolyzer at 1.55 V (inset, cell during electrolysis) in 1 M KOH. Copyright 2017 ACS, Ref. [121].

Amorphous phosphorus‐incorporated cobalt molybdenum sulfide on carbon cloth (P‐CoMoS/CC) was fabricated via hydrothermal growth of CoMoS on CC followed by phosphorization at 400°C to form a strongly coupled P‐CoMoS/CC interface with enhanced electrical contact [124]. The P‐CoMoS/CC exhibited excellent HER with a J_EHR_ of ∼130 mA/cm^2^ at 0.32 V, η_10_ of 66 mV, and a Tafel slope of 60.1 mV/dec in 1.0 M KOH, suggesting a Volmer–Heyrovsky mechanism with the Heyrovsky step as the rate‐determining step, outperforming CoMoS/CC, P‐CoS/CC, and P‐MoS/CC, and reaching the performance of Pt/C. Besides high stability for 100 h with merely 11% activity loss, attributed to the synergistic and bifunctional effect of the binary metal CoMo, anion substitution and phosphorus doping suppress surface oxidation, increase ECSA, and improve electronic conductivity, as further evidenced by EIS [124]. The enhanced activity arises from synergistic Co–Mo interaction, phosphorus‐induced electronic modulation, and an amorphous structure that exposes abundant active sites, while the strong catalyst–substrate interface and phosphorus doping improve structural stability. However, multiple reaction steps and low activity are major barriers.

Ni_0_._33_Co_0_._67_MoS_4_ nanosheets supported on carbon fiber cloth (Ni_0.33_Co_0.67_MoS_4_/CFC) were fabricated via hydrothermal growth of NiCo–(MoO_4_)^2^^−^ layered double hydroxide on CFC, followed by sulfurization under an N_2_ atmosphere to obtain a binder‐free Ni_0_._33_Co_0_._67_MoS_4_/CFC electrode with intimate contact between the catalyst and carbon cloth [125]. The Ni_0.33_Co_0.67_MoS_4_/CFC exhibited excellent HER activity in 1.0 M KOH, delivering a *J*_HER_ of ∼100 mA/cm^2^ at −0.23 V, η_10_ of 48 mV, and a Tafel slope of 73.5 mV/dec, approaching the performance of Pt/C. The Tafel slope is consistent with a Volmer–Heyrovsky HER pathway, indicating favorable reaction kinetics. It also demonstrated high stability, retaining ∼95.5% of its initial activity after 10 h of continuous operation with only a slight increase in overpotential after 1500 cycles. The enhanced HER was attributed to the synergistic interaction of Ni, Co, and Mo multivalent cations, nanosheet architecture with abundant exposed active sites, high specific surface area, and reduced charge‐transfer resistance, as evidenced by EIS analysis. The binder‐free architecture and direct growth on carbon fiber cloth contribute to the excellent electrochemical stability. However, the complex composition and multistep reaction pathways are major barriers [125].

Nickel‐doped cobalt phosphide nanoneedle arrays (Co_1‐x_Ni_x_P_3_ NAs) were fabricated via hydrothermal growth of Co_1‐x_Ni_x_(OH)F precursors followed by phosphidation, forming a binder‐free electrode with intimate contact between the Co_1‐x_Ni_x_P_3_ nanoneedles and the conductive substrate [126]. The synthesis route and morphological evolution are illustrated schematically (Figure 8a), while SEM images confirm that the vertically aligned nanoneedle architecture of Co_0.93_Ni_0.07_(OH)F is well preserved after phosphidation to form Co_0_._93_Ni_0_._07_P_3_ (Figure 8b), thereby ensuring abundant exposed active sites and efficient electron transport. The optimized Co_0.93_Ni_0.07_P_3_ displayed an η_10_ of 87 mV and gave a JHER of −100 mA/cm^2^ at −0.15 V, with a Tafel slope of 60.7 mV/dec, as shown by the polarization curves (Figure 8c), suggesting the Volmer–Heyrovsky HER pathway and indicating accelerated reaction kinetics. EIS shows even more clearly that Co_0.93_Ni_0.07_P_3_ has a lower charge‐transfer resistance than other compositions (Figure 8d). Density functional theory (DFT) calculations show that incorporating Ni to CoP_3_(200) surfaces changes the hydrogen adsorption free energy (Δ*G*_H_), especially at the P‐1 sites (Figure 8e). This brings Δ*G*_H_ closer to thermoneutral values, which speeds up HER kinetics. The Co_0.93_Ni_0.07_P_3_ was also very stable, staying efficient for more than 10 h of continuous use, attributed to the nanoneedle architecture and strong catalyst–substrate adhesion, which help maintain structural integrity during prolonged electrolysis. Co_0.93_Ni_0.07_P_3_ is more active than pure CoP_3_ and other Ni‐doping levels in the Co_1‐x_Ni_x_P_3_ series because it has better hydrogen adsorption energetics, a better nanoneedle array architecture, and better charge transfer at the interface. However, even though the kinetics are better than those of undoped CoP_3_, the HER performance is still not as good as that of commercial Pt/C [126].

**FIGURE 8:**
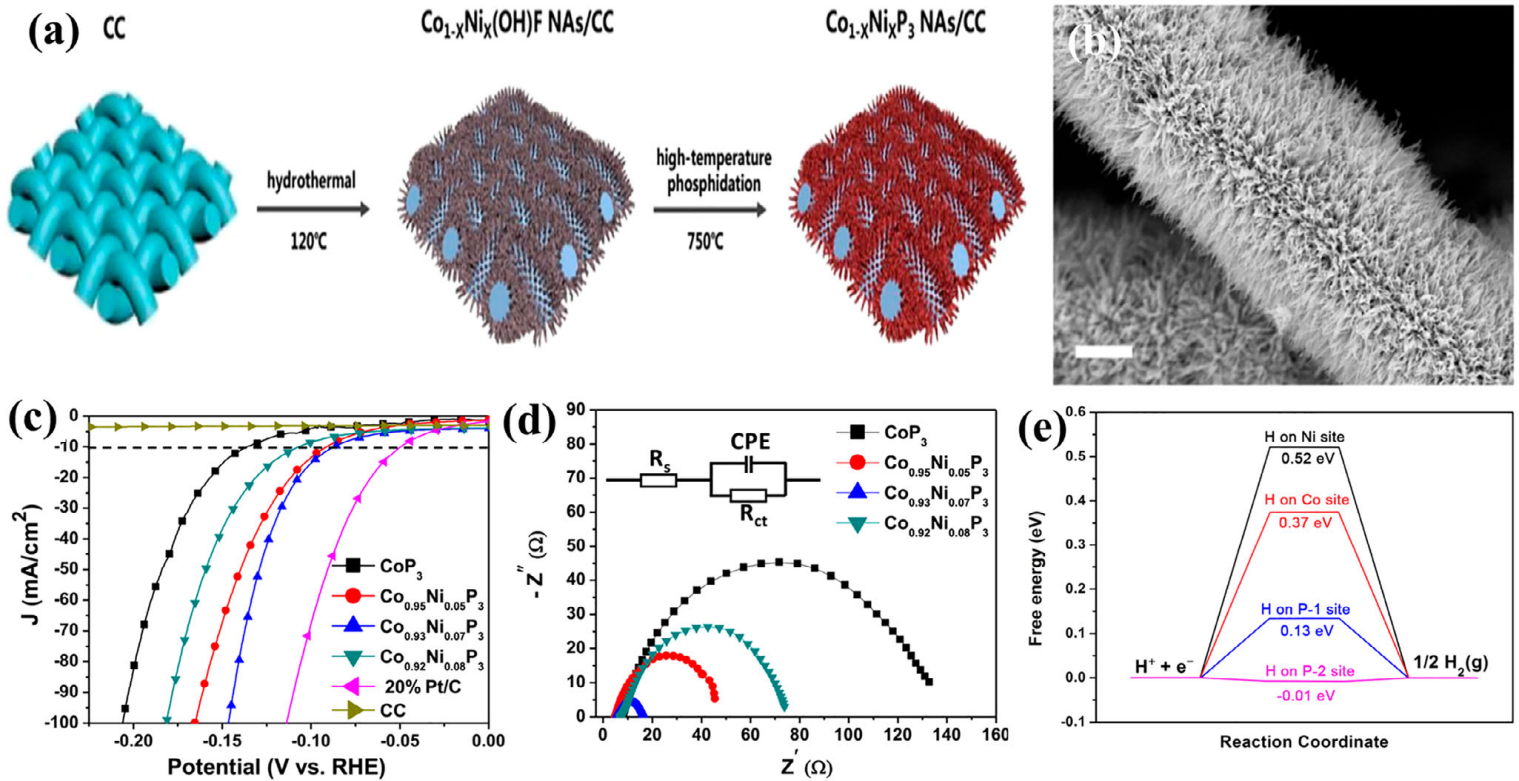
(a) Schematic illustration of the fabrication of Co_0_._93_Ni_0_._07_P_3_, (b) SEM images showing the hierarchical nanoarray morphology, (c) LSV of Co_0.93_Ni_0.07_P_3_ in 1 M KOH, (d) EIS Nyquist plots demonstrating enhanced kinetics for Ni‐doped CoP_3_, (e) DFT free‐energy profiles for H adsorption on different active sites (f). Copyright 2018 ACS, Ref. [126].

L‐cysteine‐assisted hydrothermal synthesis of Co–Mo–S/CC involved the controlled incorporation of Co into MoS_2_, forming a well‐integrated Co–Mo–S heterointerface on carbon cloth that promotes efficient charge transfer, leading to a synergistic interaction between metallic and semiconducting MoS_2_ [127]. The Co–Mo–S(1:2)/CC had a *J*_HER_ of −300 mA/cm^2^ at −0.25 V, η_10_ of 92 mV, and a Tafel slope of 82 mV/dec, which were better than those of MoS_2_/CC and CoS_2_/CC, consistent with a Volmer–Heyrovsky HER pathway, indicating improved reaction kinetics. It also demonstrated durability, maintaining ∼91% of its original current after 12 h of continuous operation and little deterioration after 1000 cycles. EIS and Cdl analysis revealed that the synergistic effect of fast intrinsic HER kinetics in metallic CoS_2_, large ECSA in MoS_2_, optimized Co/Mo composition, larger interlayer spacing, and lower charge‐transfer resistance led to improved HER performance [127]. The strong heterointerface and direct growth on carbon cloth contribute to the catalyst's electrochemical stability. However, cobalt incorporation must be carefully regulated to prevent big, low‐activity CoS_2_ particles that diminish active surface area.

Fe‐doped MoS_2_ was synthesized using a one‐pot solvothermal in situ growth technique, forming a tightly coupled Fe–MoS_2_/Ni_3_S_2_ heterointerface with strong electrical contact, resulting in spine‐like nanorods anchored on conductive Ni_3_S_2_ nanoplates [128]. The Fe–MoS_2_/Ni_3_S_2_/NF‐2 showed excellent HER activity in 1.0 M KOH, outperforming bare nickel foam and other variations with a *J*_HER_ of −200 mA/cm^2^ at −0.30 V, η_10_ of 130.6 mV, and Tafel slope of 112.7 mV/dec, which is consistent with a Volmer–Heyrovsky HER pathway, indicating enhanced reaction kinetics. After 2000 CV cycles, the MoS_2_/Ni_3_S_2_/NF‐2 was stable for 20 h. The activity originates from the synergistic interaction between Fe‐doped MoS_2_ and Ni_3_S_2_, while the heterointerface contributes to the electrochemical stability. The improved HER performance was attributed to the synergistic interaction between Fe‐doped MoS_2_ and Ni_3_S_2_, higher interlayer separation, larger ECSA, and improved charge‐transfer efficiency from the conductive nickel foam substrate. While the MoS_2_/Ni_3_S_2_/NF‐2 demonstrates enhanced activity, its performance still falls short of that of benchmark Pt‐based catalysts [128].

Electrochemical activation of CC and cathodic electrodeposition of Co‐based films with metallic Co and amorphous CoO/Co(OH)_2_ phases resulted in mixed cobalt‐based composite films (EACC–Co) [129]. The direct deposition on activated carbon cloth formed a binder‐free electrode with improved catalyst–substrate contact and enhanced surface wettability. The EACC‐Co‐500 showed superior HER activity in 0.1 M KOH, with a *J*_HER_ of ∼−25 mA/cm^2^ at −0.25 V, η_10_ of 195 mV^2^, and a Tafel slope of 142 mV/dec, exceeding that of non‐activated CC, indicating relatively sluggish HER kinetics, likely associated with limitations in the initial hydrogen adsorption step. Over 15 000 s at 10 mA/cm^2^, the EACC‐Co‐500 electrode showed stable performance with no overpotential loss. The combination of metallic Co and amorphous cobalt oxide/hydroxide phases, the higher surface defect density and hydrophilicity from electrochemical activation, and faster electron transfer at the electrode‐electrolyte interface improved HER performance. The activated CC substrate improves electrode stability by providing strong adhesion and efficient charge transport pathways. Although the EACC‐Co‐500 exhibits good activity, its long‐term stability under practical operating conditions remains limited [129].

The synthesis of MoS_2_@CoO electrocatalysts on carbon cloth (MoS_2_@CoO/CC) was achieved by hydrothermal preparation of CoO nanocrystals, sonication, and annealing with 2H‐MoS_2_ flakes to create a core‐shell architecture that promotes interfacial charge transfer and provides strong coupling between the conductive support and catalytic MoS_2_ layer [130]. MoS_2_@CoO/CC showed increased HER activity in 1.0 M KOH, with a *J*_HER_ of ∼−160 mA/cm^2^ at −0.40 V, η_10_ of 173 mV, and a Tafel slope of 129.9 mV/dec. The high Tafel slope indicates relatively sluggish HER kinetics, suggesting that the initial hydrogen adsorption step may limit the reaction rate. The MoS_2_@CoO/CC maintained 90% of its original *J*_HER_ after 1000 cyclic voltammetry cycles and steady operation over 10 h. HER performance improved due to synergistic core‐shell contact between conductive CoO and catalytically active MoS_2_ nanoflakes, increased electrochemical surface area, and reduced charge‐transfer resistance, as validated by EIS analysis. The stable core–shell structure and strong interaction between MoS_2_ and CoO contribute to improved durability by maintaining structural integrity during electrolysis. MoS_2_@CoO has a high Tafel slope, resulting in delayed HER kinetics and higher overpotential [130]. Partial catalyst separation after prolonged cycling may reduce durability.

A quaternary interface‐engineered Mo–Co–S–Se supported on carbon cloth (Mo–Co–S–Se/CC) was synthesized via solvothermal growth of MoS_2_ and CoS_2_ on carbon cloth, followed by low‐temperature selenylation to generate abundant heterointerfaces [131]. The Mo–Co–S–Se/CC showed great HER activity in 1.0 M KOH, with a *J*_HER_ of about −70 mA/cm^2^ at −0.17 V, η_10_ of 58 and η_100_ of 167 mV, and a moderate Tafel slope of 84 mV/dec, which is close to the performance of Pt/C. The Tafel slope suggests favorable HER kinetics and is consistent with a Volmer–Heyrovsky pathway. The Mo–Co–S–Se/CC also showed high durability, with almost no current decay during long chronoamperometric tests. The stable heterostructure and strong catalyst–CC interaction help maintain catalytic activity during prolonged electrolysis. Although Mo–Co–S–Se/CC shows significant performance, the interaction of multiple metal active sites is ambiguous; meanwhile, the multiple reaction steps and inferior durability (i.e., 11 h) are great barriers [131].

NiS–MoS_2_ hetero‐nanosheet arrays grown on carbon cloth (NiS–MoS_2_/CC) were synthesized via hydrothermal collaborative growth, forming vertically aligned heterostructures with abundant exposed active sites [132]. The NiS–MoS_2_/CC showed good HER performance in 1.0 M KOH, displaying a *J*_HER_ of ∼−100 mA/cm^2^ at −0.18 V, an η_10_ of ∼106 mV, and a Tafel slope of ∼56.7 mV/dec, indicating a Volmer–Heyrovsky pathway. At a *J*_HER_ of 10 mA/cm^2^, the Mo–Co–S–Se/CC demonstrated exceptional long‐term stability for the HER, maintaining its activity for 24 h. This improved performance can be attributed to the synergistic interaction between NiS and MoS_2_, which results in more readily available active sites and accelerated charge transfer facilitated by the vertically oriented nanosheet structure, also attributed to the robust catalyst–carbon cloth interface, which suppresses catalyst detachment and preserves electrical contact throughout prolonged electrolysis. While HER activity has improved, the NiS–MoS_2_ requires higher overpotentials than noble‐metal benchmarks and relies on heterointerface formation and nanosheet alignment, which may limit long‐term stability and large‐scale applications [132].

Nitrogen‐doped cobalt phosphide nanoneedles (N‐doped CoP_2_@PCC) were developed through a multistep process, including thermal treatment and acid etching, followed by hydrothermal growth to form a binder‐free electrode with strong interfacial contact between the CoP_2_ nanoneedles and porous carbon cloth [133]. A hierarchical architecture of densely packed nanoneedles uniformly anchored on porous carbon fibers provides ample exposed active sites and increased electrolyte accessibility, as shown in the schematic and SEM images (Figure 9a–c). N‐doped CoP_2_@PCC has a considerable HER activity in 1 M KOH. The polarization curve (Figure 9d) shows an η_10_ of 64 mV and a high *J*_HER_ of −200 mA/cm^2^ at −0.14 V, with a Tafel slope of 47.4 mV/dec, consistent with a Volmer–Heyrovsky mechanism in which the electrochemical desorption step is rate‐limiting. EIS reveals that N‐doped CoP_2_@PCC exhibits lower charge‐transfer resistance than undoped CoP_2_@PCC (Figure 9e). Thus, electrons can move faster between the two materials. DFT calculations (Figure 9f) show that nitrogen doping enhances hydrogen adsorption efficiency, with the P site having a ΔGH value that is close to thermoneutral (−0.064 eV). Therefore, it is the main active site that accelerates HER activity. The N‐doped CoP_2_@ PCC is also very durable, with stable HER performance for 15 h in 1 M KOH and no noticeable drop in activity [133]. Even though the catalyst performs better for alkaline HER, it still fails to match Pt/C at low overpotentials, and the multistep reaction remains a major problem.

**FIGURE 9:**
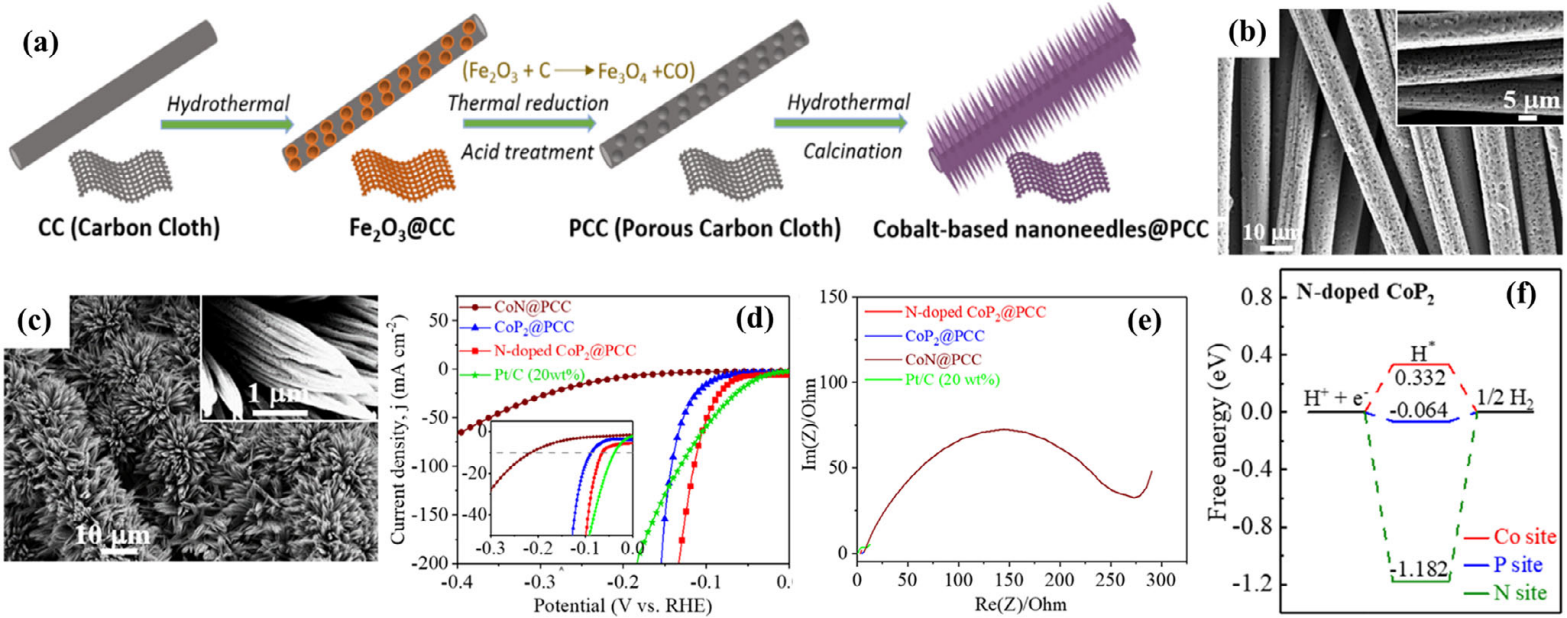
(a) Schematic illustration of the fabrication of N‐doped CoP_2_@PCC, (b, c) SEM images showing nanoneedles on porous carbon cloth, (d) LSV of N‐doped CoP_2_@PCC in 1 M KOH, (e) EIS Nyquist plots demonstrating enhanced kinetics, (f) DFT‐calculated hydrogen adsorption free energies identifying P sites as the dominant active centers. Copyright 2019 ACS, Ref. [133].

A one‐pot electrodeposition–phosphorization technique produced numerous hetero‐nanointerfaces embedded in an amorphous matrix as manganese oxide–cobalt phosphide nanohybrid nanosheet arrays supported on carbon cloth (Mn‐O@Co‐P/CC) forming a binder‐free electrode [134]. In 1.0 M KOH, Mn‐O@Co‐P/CC showed strong HER activity, with a *J*_HER_ of ∼‐150 mA/cm^2^ at ‐0.20 V, η_10_ of 106 mV, and a Tafel slope of 56 mV/dec, suggesting the Volmer–Heyrovsky HER pathway. The Mn‐O@Co‐P/CC demonstrated considerable stability throughout 12 h of chronopotentiometric testing. DFT calculations and EIS analysis suggest that the synergistic Mn‐O/Co‐P interfacial sites promote water dissociation via a frustrated Lewis pair‐like mechanism. This, in turn, increases the ECSA and reduces charge‐transfer resistance, thereby enhancing HER activity. However, the Mn‐O@Co‐P/CC performance relies on a complicated heterointerface contained in an amorphous matrix, which may make active site control difficult and repeatability, and large‐scale production difficult [134].

Co(OH)_2_‐modified MoS_2_ heterostructures on CC (Co(OH)_2_/MoS_2_/CC) were formed by hydrothermally growing MoS_2_ nanosheets and electrochemically depositing amorphous Co(OH)_2_, forming a binder‐free heterostructure [135]. The Co(OH)_2_/MoS_2_/CC showed higher HER activity in 1.0 M KOH, with a *J*_HER_ of ∼−200 mA/cm^2^ at −0.25 V, η_10_ of 117 mV, and a Tafel slope of ∼59.8 mV/dec, exceeding MoS_2_/CC and Co(OH)_2_. The Tafel slope is consistent with a Volmer–Heyrovsky HER pathway, indicating accelerated reaction kinetics. Stable after 3000 CV cycles, with no overpotential rise or current loss after 12 h. Under light irradiation, conductivity and exchange *J*_HER_ increased, improving HER performance. The interface's interaction with Co(OH)_2_ and MoS_2_ led to improved activity, a finding supported by DFT calculations and electrochemical impedance analysis. Co(OH)_2_ facilitates water dissociation and hydroxyl adsorption, while MoS_2_ promotes hydrogen absorption and H_2_ release. The strong heterointerface and direct growth on carbon cloth contribute to excellent electrochemical stability by maintaining efficient electrical contact during prolonged operation [135]. However, the presence of multiple reaction steps presents a significant obstacle.

3D nitrogen‐doped carbon‐coated NiCo_2_S_4_ nanowires (NAC@NiCo_2_S_4_ NWs/CC) were synthesized via hydrothermal growth of NiCo precursor nanowires and annealing with thiourea and ethylene glycol to add sulfur, nitrogen, and an ultrathin carbon coating, forming a binder‐free core–shell structure [136]. The NAC@NiCo_2_S_4_ NWs/CC outperformed undoped and non‐carbon‐coated counterparts with HER *J*_HER_ of ∼−300 mA/cm^2^ at −0.30 V, η_10_ of 254 mV, and Tafel slope of 57 mV/dec, suggesting the Volmer–Heyrovsky HER pathway, indicating favorable reaction kinetics. Stability for 20 h of continuous operation. HER performance improved due to nitrogen anion doping and carbon encapsulation, which modulate the electronic structure, increase electrochemically active site density, electrical conductivity, and charge‐transfer kinetics in the 3D nanowire structure. The architecture contributes to the excellent electrochemical stability by maintaining efficient electron transport and structural integrity during prolonged electrolysis. The NAC@NiCo_2_S_4_ NWs/CC needs higher driving voltages than noble‐metal standards despite its high *J*_HER_ at large overpotentials. However, multiple reaction steps pose a significant barrier [136].

The synthesis of Ni/Ni(OH)_2_/NiOOH nanoworms on carbon cloth (Ni/Ni(OH)_2_/NiOOH/CC) using a simple electrodeposition approach created a porous heterostructure comprising metallic Ni, Ni(OH)_2_, and NiOOH species forming a binder‐free electrode [137]. In 1.0 M KOH, Ni/Ni(OH)_2_/NiOOH/CC demonstrated enhanced HER activity, with a *J*_HER_ of −200 mA/cm^2^ at −0.37 V, a low η_10_ of 45 mV, and a Tafel slope of 99.6 mV/dec, suggesting the Volmer–Heyrovsky HER pathway, indicating improved reaction kinetics. At lower *J*_HER_, its performance was similar to that of Pt/C. It also maintained a constant current for 200 h, and no degradation was observed after 1000 cycles. Improved HER performance resulted from synergistic interaction between metallic Ni and surface Ni(OH)_2_/NiOOH species. Nickel improves hydrogen adsorption, while hydroxide and oxyhydroxide phases enhance water dissociation and the release of hydroxide ions. High ECSA and low charge‐transfer resistance facilitate and contribute to the outstanding long‐term electrochemical stability. However, the catalytic activity still falls short of that of Pt‐based benchmark catalysts [137].

A nanohybrid heterostructure was created by combining NiMo alloy with Ni(OH)_2_ nanosheets grown on carbon cloth (NiMo/Ni(OH)_2_/CC) by hydrothermal growth and electrodeposition, forming a binder‐free electrode architecture [138]. NiMo/Ni(OH)_2_/CC showed increased HER activity in 1.0 M KOH, with a *J*_HER_ of ∼−80 mA/cm^2^ at −0.30 V, η_10_ of 132 mV, and a Tafel slope of 134.1 mV/dec, suggesting the hydrogen adsorption step remains a kinetic limitation. A slight decrease in HER activity was seen in long‐term stability, with the overpotential rising from ∼130 mV to ∼180 mV after 24 h at −10 mA/cm^2^. The improved HER was due to the interaction between the NiMo alloy and Ni(OH)_2_, which breaks down water, creating a site for hydrogen attachment and facilitating charge transfer at the interface. However, the presence of multiple reaction steps presents a significant obstacle [138].

CoSe_2_/MoSe_2_ heterostructures were synthesized on carbon cloth (CoSe_2_/MoSe_2_/CC) by impregnation, with the autoclave kept at 150°C for 6 h, followed by annealing at 400°C for 2 h and selenization‐induced phase separation of CoMoO_4_ nanosheet arrays, resulting in uniformly attached CoSe_2_ NPs [139]. The CoSe_2_/MoSe_2_/CC showed superior HER activity in 1.0 M KOH, with a *J*_HER_ of ∼−80 mA/cm^2^ at −0.20 V, η_10_ of 90 mV, and a Tafel slope of 84.8 mV/dec, suggesting it follows the Volmer–Heyrovsky pathway, exceeding CoSe_2_ and MoSe_2_. CoSe_2_/MoSe_2_/CC also showed good stability for 12 h. Synergistic coupling of metallic CoSe_2_ and MoSe_2_, hierarchical 0D–2D architecture, prevention of nanosheet restacking, and faster charge‐transfer kinetics led to improved HER performance, as evidenced by EIS and ECSA. However, the long‐term stability of the catalyst under real‐world conditions hasn't been studied enough [139].

One‐dimensional MnCo_2_O_4_/graphdiyne heterojunction nanowire arrays on carbon cloth (NW‐MnCo_2_O_4_/GDY) were synthesized via in situ hydrothermal growth and surface coupling with an ultrathin graphdiyne layer, forming an integrated core–shell heterointerface that facilitates interfacial charge transfer, resulting in a strong core–shell architecture [140]. NW‐MnCo_2_O_4_/GDY showed strong HER performance in 1.0 M KOH, with a *J*_HER_ of ∼−500 mA/cm^2^ at −0.70 V, η_10_ of 180 mV, and a Tafel slope of 65 mV dec. The Tafel slope is consistent with a Volmer–Heyrovsky HER pathway, indicating improved reaction kinetics. It also maintained HER activity after 40 h of continuous operation and 3500 cyclic voltammetry cycles. Electrochemical measurements and DFT calculations confirmed that synergistic interfacial coupling between MnCo_2_O_4_ and graphdiyne improved performance by increasing electronic conductivity, water dissociation, hydrogen adsorption at acetylenic carbon sites, and charge‐transfer kinetics. The MnCo_2_O_4_/GDY interface and strong anchoring on CC contribute to structural stability by preserving electrical contact during prolonged electrolysis. The effectiveness of this process depends on the integrity of the contact between MnCo_2_O_4_ and graphdiyne, which could hinder large‐scale manufacturing [140].

Se‐NiCoP/CC hierarchical nanoarrays were synthesized via a hydrothermal reaction, phosphorization, and selenization, forming a well‐integrated Se‐NiCoP/CC interface [141]. The Se dopant improved the number of electrochemically active sites and matched the d‐band structure of NiCoP. As a result, Se‐NiCoP/CC showed better HER performance than both Se‐NiCoP and CC. Se‐NiCoP/CC‐2 showed exceptional HER activity in 1.0 M KOH, with a high *J*_HER_ of ∼−350 mA/cm^2^ at −0.2, η_10_ of 79, and a Tafel slope of 81.6 mV dec, suggesting the Volmer–Heyrovsky HER pathway, indicating favorable reaction kinetics. Se‐NiCoP‐2/CC demonstrated remarkable stability over 20 h, exhibiting no decline in *J*_HER_ following 1000 CV sweeps within the elevated overpotential region [141]. The excellent durability is attributed to the robust nanoarray architecture, strong catalyst–carbon cloth adhesion, and stable Se‐modified NiCoP framework that maintains active sites during electrolysis. Conversely, the material's activity is considerably inferior to that of commercial Pt/C, compounded by the substantial ternary metal composition and the intricate nature of its synthesis.

Tin‐doped cobalt disulfide nanowire arrays grown on carbon cloth (Sn‐CoS_2_/CC) were synthesized via hydrothermal synthesis of Sn‐doped Co(OH)_2_ precursors followed by sulfurization, forming binder‐free porous pyrite‐structured CoS_2_ nanowires with uniformly incorporated Sn dopants [142]. The Sn‐CoS_2_/CC exhibited enhanced HER activity in 1.0 M KOH, displaying a *J*_HER_ of ∼−100 mA/cm^2^ at −0.30 V, η_10_ of 161 mV, and a Tafel slope of 94 mV/dec. The Sn‐CoS_2_/CC also demonstrated excellent durability, showing only ∼5 mV overpotential increase after 32 h of continuous operation and negligible degradation after 1000 cyclic voltammetry cycles. The HER performance was attributed to Sn‐induced electronic structure modulation, enhanced electrical conductivity, and activation of previously inert sulfur sites with optimized hydrogen adsorption free energy, as supported by electrochemical analysis and DFT calculations [142]. The robust nanowire architecture and strong catalyst–carbon cloth interaction contribute to the excellent stability by maintaining structural integrity and efficient charge transport during electrolysis. However, multiple reaction steps are also a great barrier.

WS_2_ 3D dendritic nanostructures with iron and phosphorus co‐doped 1T‐phase (1T‐Fe/P‐WS_2_@CC) were synthesized using a hydrothermal method, as shown in Figure 10a [143]. This allowed for simultaneous incorporation and stabilization of the metallic 1T phase. SEM and TEM images (Figure 10b–d) show a 3D dendritic shape that suggests an agave leaf, with ultrathin nanosheets lined up vertically and rough surfaces that provide many active sites and good pathways for electron transport. Elemental mapping confirms the homogeneous distribution of W, S, Fe, and P (Figure 10e). Pure WS_2_@CC, singly doped Fe‐WS_2_@CC, and P‐WS_2_@CC exhibit less developed, more compact morphologies, resulting in lower catalytic activity. Structural advantages lead to better HER performance, as shown in Figure 10f. 1T‐Fe/P‐WS_2_@CC achieves current densities equivalent to Pt/C and much greater than WS_2_‐based counterparts. In 1 M KOH, the 1T‐Fe/P‐WS_2_@CC displays η_10_ of 116 mV. 1T‐Fe/P‐WS_2_@CC delivers a JHER of ∼150 mA/cm^2^ at −0.25 V. Figure 10g shows a Tafel slope of 65 mV/dec, much lower than WS_2_@CC, Fe‐WS_2_@CC, and P‐WS_2_@CC, indicating faster Volmer–Heyrovsky HER kinetics due to Fe/P co‐doping and 1T‐phase enrichment. 1T‐Fe/P‐WS_2_@CC has the highest double‐layer capacitance (Figure 10h) and the lowest charge‐transfer resistance among WS_2_‐based electrodes (Figure 10i), indicating accelerated charge transport. The 1T‐Fe/P‐WS_2_@CC maintains a nearly constant potential for 24 h of continuous HER operation at 50 mA/cm^2^ (Figure 10f), due to steady 1T‐phase enrichment and a strong 3D dendritic architecture anchored on carbon cloth. 1T‐Fe/P‐WS_2_@CC is very durable and has much better HER activity than pristine and singly doped WS_2_ catalysts.CC still has slightly higher overpotentials than commercial Pt/C, which indicates that more research needs to be done to improve the density of active sites and the energetics of hydrogen adsorption to fully match noble‐metal benchmarks [143].

**FIGURE 10:**
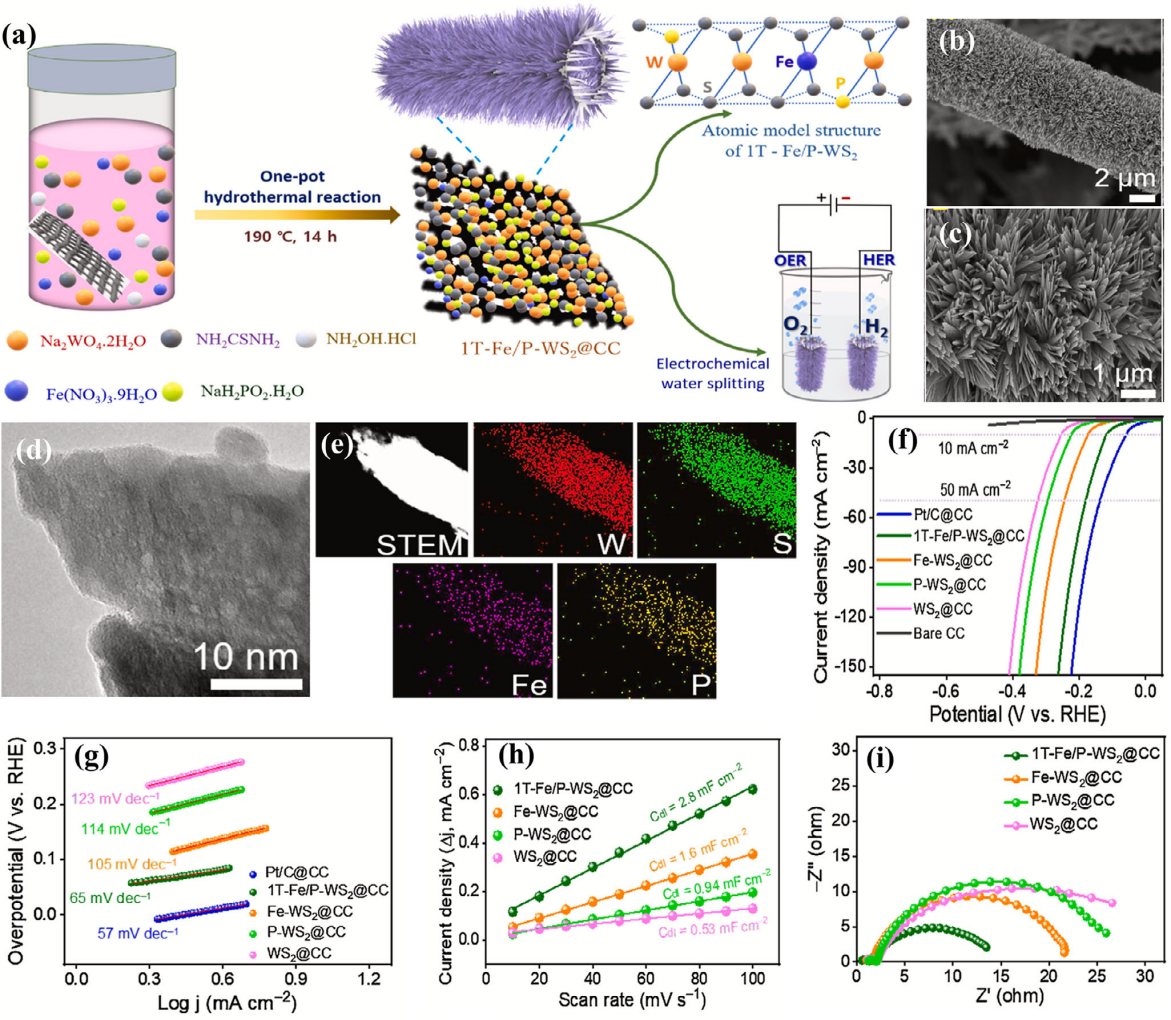
(a) Schematic illustration of the fabrication of 1T‐Fe/P‐WS_2_@CC, (b, c) FE‐SEM images at different magnifications from low to high, (d) TEM, (e) Elemental Mapping, (f) LSV, (g) Tafel slope, (h) *J*_HER_ of 1T‐Fe/P‐WS_2_@CC compared with others, and (i) EIS Nyquist plots, all recorded in 1 M KOH. Copyright 2021, Elsevier, Ref. [143].

Co_9_S_8_‐V_3_S_4_ heterojunction nanowires on carbon cloth (Co_9_S_8_‐V_3_S_4_/CC) were created using a hydrothermal process. This involved growing Co‐V precursor nanowires and then controlling the sulfurization. The result was a well‐defined one‐dimensional heterostructure with strong electrical connections [144]. The Co_9_S_8_‐V_3_S_4_/CC showed strong HER activity in 1.0 M KOH, with a *J*_HER_ of ∼−200 mA/cm^2^ at −0.30 V, η_10_ of 85 mV, and a Tafel slope of 51 mV/dec, suggesting a Volmer–Heyrovsky HER pathway and indicating accelerated reaction kinetics. *J*_HER_ remained steady after 40 h of continuous operation and 3000 cyclic voltammetry cycles. The excellent durability is attributed to the robust one‐dimensional nanowire architecture and strong catalyst–carbon cloth interaction, which preserve structural integrity and electrical connectivity during electrolysis. The Co_9_S_8_–V_3_S_4_ heterojunction enhances hydrogen adsorption energy, charge‐carrier density, and charge‐transfer kinetics, leading to superior HER performance, as confirmed by electrochemical experiments and DFT. However, multiple reaction steps are also a great barrier [144].

Zinc–nickel sulfide coupled with molybdenum carbide on carbon cloth (Zn_x_Ni_1‐x_S@Mo_2_C/CC) was prepared via hydrothermal growth and a sulfurization process. This process produced a porous heterostructure with many defect sites [145]. The Zn_x_Ni_1‐x_S@Mo_2_C/CC displayed enhanced HER activity in 1.0 M KOH, with a *J*_HER_ of −100 mA/cm^2^ at −0.40 V, η_10_ of 230 mV, and a Tafel slope of 189 mV/dec, suggesting that the initial hydrogen adsorption or water dissociation step remains a major kinetic limitation. It also showed good durability, maintaining stable HER performance during long chronoamperometry tests. The improved HER activity was due to the synergistic effect of Zn_x_Ni_1‐x_S and Mo_2_C, the increased ECSA from surface engineering, and the enhanced charge‐transfer efficiency from the conductive carbon cloth substrate [145]. However, the relatively high Tafel slope indicates sluggish HER kinetics, suggesting that further optimization is required to improve the catalytic efficiency.

Fe‐doped cobalt sulfide nanoflakes were hydrothermally synthesized directly on carbon cloth (Fe–Co–S/CC) and annealed, resulting in binder‐free, self‐supported, hierarchical nanoflakes flakes [146]. The optimized Fe–Co–S/CC‐75 showed HER activity in 1.0 M KOH, with a *J*_HER_ of −150 mA/cm^2^ at ‐0.52 V, η_10_ of 320 mV, and a Tafel slope of 82.6 mV/dec. The Tafel slope indicates moderate HER kinetics and suggests improved reaction rates compared with the undoped catalyst. Therefore, HER kinetics were modest, and the Fe–Co–S/CC‐75 maintained ∼72.5% of its initial *J*_HER_ after 48 h of continuous operation. The enhanced HER performance stemmed from Fe‐induced alterations in the electronic structure, the increased ECSA of the nanoflake architecture, and the improved charge‐transfer efficiency of the self‐standing carbon cloth support. The direct growth on carbon cloth and hierarchical nanoflake structure contribute to durability by maintaining catalyst adhesion and accessible active sites during prolonged electrolysis. Nevertheless, the relatively high overpotential indicates that additional activity enhancements are necessary [146].

The heterostructured Co_3_O_4_/VO_2_ nanosheet arrays on carbon cloth (Co_3_O_4_/VO_2_/CC) were grown by hydrothermally growing VO_2_ nanosheets, electrodepositing Co(OH)_2_, and mildly oxidizing it to Co_3_O_4_ to establish a distinct oxide–oxide heterointerface [147]. In 1.0 M KOH, Co_3_O_4_/VO_2_/CC displayed a *J*_HER_ of −100 mA/cm^2^ at −0.20 V, an η_10_ of 108 mV, and a Tafel slope of 98 mV/dec, indicating moderate HER kinetics. The Co_3_O_4_/VO_2_/CC showed stability for 10 h. Electrochemical investigations and DFT calculations reveal that the interfacial electronic coupling between Co_3_O_4_ and VO_2_ enhances HER performance. The improvement stems from optimizing hydrogen adsorption energy, increasing the ECSA, and speeding up electron transport. The robust oxide–oxide heterointerface and direct growth on carbon cloth contribute to maintaining catalyst integrity and electrical contact during electrolysis. Although the Co_3_O_4_/VO_2_ heterostructure shows better HER activity, its reaction kinetics are relatively slow, as indicated by a high Tafel slope [147].

Bimetallic drum‐like structures (CoP_3_‐Nb_2_P/PCC) were produced by hydrothermal growth of Co–Nb oxide precursors and high‐temperature phosphorization, forming a strongly coupled CoP_3_/Nb_2_P heterointerface with direct integration on plasma‐treated carbon cloth [148]. CoP_3_‐Nb_2_P/PCC showed strong HER activity in 1.0 M KOH, with a *J*_HER_ of −500 mA/cm^2^ at −0.30 V vs. RHE, η_10_ of 111 mV, and a Tafel slope of 72.8 mV/dec, suggesting the Volmer–Heyrovsky HER pathway, indicating accelerated reaction kinetics. CoP_3_‐Nb_2_P/PCC outperformed commercial Pt/C in HER performance at current densities above ∼200 mA/cm^2^ and maintained over 93% of its original *J*_HER_ after 50 h of continuous operation. Better performance was observed through electronic interactions between CoP_3_ and Nb_2_P phases, plasma‐induced defect engineering of the carbon cloth substrate, optimized hydrogen adsorption free energy, and enhanced charge‐transfer kinetics, as determined by in situ Raman spectroscopy and DFT calculations. Despite outstanding high‐current HER performance, plasma treatment and high‐temperature phosphorization increase process complexity and may impede scalability and cost‐effective large‐scale manufacturing [148].

Ru–CoP NAs/CC, a hierarchical, porous nanoarray architecture with evenly integrated Ru dopants, was produced using a MOF‐derived method and phosphorization [149]. The optimized Ru–CoP‐2.5 NAs showed remarkable HER activity in 1.0 M KOH, with a *J*_HER_ of ∼−160 mA/cm^2^ at −0.15 V, a low η_10_ of 52 mV, and a modest Tafel slope of 39.7 mV/dec, suggesting rapid kinetics. The Ru–CoP‐2.5 NAs performed well after 5000 CV cycles and 50 h at high *J*_HER_. Electrochemical measurements and DFT calculations showed that Ru‐induced modulation of the CoP electronic structure, optimized hydrogen adsorption free energy, faster charge‐transfer kinetics, and exposed active sites from the leaf‐like porous nanoarray structure improved HER performance. However, multiple reaction steps and inferior HER performance are major barriers [149].

MoS_2_/Ru heterostructures grown directly on carbon cloth (MoS_2_/Ru@CC) were synthesized via a hydrothermal/solvothermal process, where the ultrafine Ru NPs (∼2.5 nm) were uniformly anchored on MoS_2_ nanosheets, forming a Ru–MoS_2_ interface that promotes electronic interaction and efficient charge transfer [150]. The MoS_2_/Ru@CC showed better HER activity in 1.0 M KOH, with a *J*_HER_ of −100 mA/cm^2^ at −0.20 V, a η_10_ of 71.3 mV, and a Tafel slope of 104.8 mV/dec, indicating moderate HER kinetics, and the reaction pathway cannot be conclusively determined from the Tafel value alone. MoS_2_/Ru@CC also showed good durability, maintaining its performance well for at least 35 h of continuous operation. The improved HER activity was due to the synergistic interaction between Ru and MoS_2_. However, multiple reaction steps are a major barrier [150].

MoS_2_ nanosheets were produced on a carbon nanotube–carbon cloth substrate (MoS_2_/CNTs/CC) using a hydrothermal technique, with oxalic acid regulating size and dispersion, forming an integrated interface that facilitates electron transport and improves catalyst accessibility [151]. In 1.0 M KOH, the MoS_2_/CNTs/CC showed significant HER activity, with a *J*_HER_ of ‐105 mA/cm^2^ at ‐0.22 V, η_10_ of 134 mV, and a low Tafel slope of 45.7 mV/dec, suggesting the Volmer–Heyrovsky HER pathway, indicating favorable reaction kinetics. After 10 h of continuous operation, it showed no performance loss. Smaller MoS_2_ nanosheets have more active edge sites, improving HER performance. The CNT network conducts electricity better. Depending on the stability of the MoS_2_ nanosheets and the CNT network, its performance may be less durable and less scalable over time [151].

Amorphous Co‐Ni‐S nanoflake arrays (CO_4_NiS/CC) were synthesized by hydrothermal growth of mixed precursors and moderate liquid‐phase vulcanization, forming a binder‐free electrode [152]. The CO_4_NiS/CC showed considerable HER activity in 1.0 M KOH, with a *J*_HER_ of ‐75 mA/cm^2^ at ‐0.30 V, an η_10_ of 192 mV, and a Tafel slope of 119 mV/dec, indicating Volmer‐Heyrovsky kinetics. The CO_4_NiS/CC showed good stability. After 36 h of operation at a *J*_HER_ of 10 mA/cm^2^, it maintained 97% of its HER activity. The excellent durability is attributed to the amorphous structure, which provides structural flexibility, and the strong interaction between the catalyst and carbon fabric that preserves active sites during electrolysis. An amorphous structure with widely dispersed Co–Ni active sites, an ECSA, and a carbon fabric support promoted charge transfer and increased HER performance [152]. However, a high Tafel slope slows catalyst kinetics, potentially limiting its competitiveness relative to superior HER catalysts.

A hydrophilic bimetallic phosphate, AgCoPO_4_ nanorods, was produced on carbon fiber paper (AgCoPO_4_/CFP) by hydrothermal growth of an AgCo(CO)_4_ precursor and thermal phosphorization, forming a self‐supported nanorod architecture with a strongly coupled Ag–Co phosphate interface that facilitates electron transport and exposes abundant active sites [153]. The AgCoPO_4_/CFP showed high HER activity in 1.0 M NaOH, with a *J*_HER_ of ∼−150 mA/cm^2^ at −0.30 V, η_10_ of 32 mV, and a Tafel slope of 34.4 mV/dec, comparable to Pt/C, indicating highly favorable HER kinetics and a Volmer–Heyrovsky reaction pathway, and exhibited great durability, as it continued to operate for 25 h. The excellent stability is attributed to the robust nanorod structure, strong catalyst–carbon fiber paper interaction, and stable Ag–Co phosphate framework that maintains active sites during prolonged. The synergistic interaction between the Ag and Co centers, greater surface wettability after phosphorization, better electronic structure, and quicker charge‐transfer kinetics from the self‐supported nanorod design increased HER activity. However, high energy demand and the use of the noble metal Ag, besides low *J*_HER_, are major issues [153].

Direct growth on carbon cloth establishes intimate interfacial contact between the catalyst and the conductive substrate, minimizing interfacial resistance and enabling efficient electron transport during HER. This binder‐free architecture also improves mechanical adhesion, thereby enhancing electrode durability under continuous electrolysis.

Bimetallic cobalt–nickel selenide nanosheets, electrodeposited on carbon cloth (CoNiSe‐CC), were prepared using the electrodeposition of Co–Ni hydroxides, followed by low‐temperature selenization, resulting in vertically aligned, ultrathin nanosheet arrays firmly bonded to the conductive substrate [154]. The CoNiSe‐CC demonstrated effective HER activity in 1.0 M KOH, with a *J*_HER_ of around −15 mA/cm^2^ at −0.20 V, η_10_ of 180 mV, and a Tafel slope of 63.1 mV/dec, suggesting Volmer–Heyrovsky HER pathway, indicating favorable reaction kinetics. The CoNiSe‐CC also demonstrated good stability for 20 h. The durability is attributed to the vertically aligned nanosheet architecture and strong catalyst–carbon cloth adhesion, which preserve accessible active sites and maintain electrical connectivity during electrolysis. The increased activity was due to the synergistic electronic interaction between the Co and Ni centers, the improved electrical conductivity from selenization, the many exposed active sites from the nanosheet shape, and the lower charge‐transfer resistance at the catalyst–carbon cloth interface. But the impact of multiple metal active sites on the HER is not underlined enough; meanwhile, the effect of stability on the structural morphology is not reported [154].

Fe‐doped nickel cobalt selenide nanorod arrays (Fe–NiCo_2_Se_4_/CC) were synthesized via hydrothermal growth followed by selenization, yielding vertically oriented, textured nanorods [155]. The optimized Fe_0.2_NiCo_1.8_Se_4_/CC showed better HER activity in a 1.0 M KOH solution. It had a *J*_HER_ of ‐700 mA/cm^2^ at ‐0.75 V, a η_@50_ of 148 mV^2^, and a Tafel slope of 85.7 mV/dec, which points to a Volmer–Heyrovsky mechanism. The Fe–NiCo_2_Se_4_/CC maintained stable *J*_HER_ throughout 48 h of chronoamperometric testing with no performance loss. The excellent durability is attributed to the robust vertically aligned nanorod architecture, strong catalyst–substrate interaction, and preservation of active sites during prolonged electrolysis. The HER activity was due to Fe^3^^+^‐induced lattice distortion, increased ECSA, decreased charge‐transfer resistance, and robust Fe, Ni, and Co active sites. EIS and DFT indicated better electronic conductivity. Despite *J*_HER's_ strong capabilities, several stages of the response represent a barrier [155].

Fe‐doped molybdenum disulfide nanosheets grown on carbon nanotube–modified carbon cloth (Fe_x_Mo_1‐x_S_2_/CNT@CC) were synthesized via a hydrothermal method by tuning the Fe doping concentration, creating a conductive heterointerface that improves electron transport and provides strong catalyst anchoring [156]. The optimized Fe_0.05_Mo_0.95_S_2_/CNT@CC showed better HER activity in 1.0 M KOH, with a *J*_HER_ of −120 mA/cm^2^ at −0.23 V, an η_10_ of 116 mV, and a Tafel slope of 44.7 mV/dec, indicating a Volmer–Heyrovsky reaction pathway. The enhanced HER performance resulted from Fe‐induced activation of MoS_2_ basal planes, an increased ECSA, and diminished charge‐transfer resistance due to the synergistic interaction between Fe_x_Mo_1‐x_S_2_ nanosheets and the conductive CNT network, as validated by CV and EIS analyses. The robust Fe‐doped MoS_2_/CNT interface also contributes to stability by maintaining electrical connectivity and preventing catalyst aggregation during electrolysis. The inferior activity, although using multi‐metal active sites, raises questions and increases the cost [156].

Nitrogen‐doped Fe_7_S_8_ nanostructures supported on carbon cloth (N‐Fe_7_S_8_/CC) were synthesized through solvothermal methods, starting with FeOOH on CC, then sulfurization, and finally ammonia annealing to add nitrogen dopants, forming a strongly integrated N‐Fe_7_S_8_/carbon cloth interface with stable catalyst anchoring and improved electron transport pathways [157]. The N‐Fe_7_S_8_/CC displayed enhanced HER activity in 1.0 M KOH, with a JHER of −40 mA/cm^2^ at −0.15 V, an η_10_ of 89 mV, and a Tafel slope of 105 mV/dec, indicating moderate HER kinetics. XPS, XAFS, and DFT showed that the improved HER performance was due to nitrogen altering the electronic structure of Fe_7_S_8_, thereby making it more conductive and accelerating water dissociation. The N‐Fe_7_S_8_/CC is extremely stable, functioning consistently at 100 mA/cm^2^ for 100 h with minimal change in potential. However, multiple reaction steps are a major barrier [157].

Co_0.75_Mo_3_S_3.75_/CoS_2_@2D‐MoS_2_ heterostructured nanosheets directly grown on carbon cloth (Co–Mo–S@MoS_2_/CC) were synthesized via a hydrothermal self‐assembly strategy to form a binder‐free electrode [158]. The optimized Co–Mo–S@MoS_2_/CC exhibited strong HER activity in 1.0 M KOH, displaying a high *J*_HER_ of −130 mA/cm^2^ at −0.30 V, with a low overpotential of 43 mV at 20 mA/cm^2^ and a small Tafel slope of ∼36.6 mV/dec, indicating favorable Volmer–Heyrovsky kinetics. The improved HER performance was due to the synergistic coupling of Co_0.75_Mo_3_S_3.75_, CoS_2_, and MoS_2_ phases, cobalt‐induced electronic modulation of MoS_2_, a larger ECSA, and a lower charge‐transfer resistance. It also showed great long‐term stability, lasting 51 h of electrolysis. After 500 cycles of continuous CV cycling, HER activity showed a slight but not significant decline. However, the HER activity was sensitive to cobalt content, and excessive Co incorporation partially suppressed MoS_2_ active sites, limiting further improvement at lower overpotentials [158].

MoS_2_ nanosheets grown on three‐dimensional carbon cloth (UVO‐MoS_2_/CC) were synthesized via a hydrothermal method, followed by ultraviolet–ozone (UVO) post‐treatment to introduce surface defects, providing a binder‐free electrode architecture, while UVO treatment modifies the surface/interface by generating defect‐rich active regions and improving catalyst accessibility [159]. The optimally treated UVO‐50 MoS_2_/CC exhibited better HER activity in 1.0 M KOH, with a *J*_HER_ of −100 mA/cm^2^ at −0.23 V, an η_10_ of 116 mV, and a Tafel slope of 64 mV/dec, indicating improved HER kinetics governed by the Volmer–Heyrovsky mechanism. The superior performance was due to UVO‐induced sulfur vacancies, increased density of edge sites, formation of amorphous oxygen‐incorporated MoS_x_, and enhanced electronic conductivity, which was supported by XRD, XPS, CV‐derived double‐layer capacitance, and EIS analyses. However, excessive UVO exposure leads to partial amorphization of MoS_2_, which may compromise structural integrity and limit long‐term stability at higher current densities [159].

Porous FeP/FeO heterostructures deposited on carbon cloth (FeP/FeO/CC) were synthesized via hydrothermal growth of iron oxide on carbon cloth, followed by thermal treatment in air and subsequent phosphorization under an argon atmosphere, resulting in a loose, porous morphology [160]. The oxidation–phosphorization process creates an intimate FeP/FeO heterointerface directly anchored on carbon cloth, enabling efficient interfacial charge transfer and strong catalyst–substrate interaction. The FeP/FeO/CC demonstrated improved HER activity in 1.0 M KOH, achieving a *J*_HER_ of −300 mA/cm^2^ at −0.30 V, an η_10_ of 117 mV, and a Tafel slope of 96.2 mV/dec, indicating moderate HER kinetics, suggesting that intrinsic reaction steps remain kinetically limiting despite the improved catalytic activity. The FeP/FeO/CC exhibited stability throughout 20 h of continuous chronoamperometric testing, with a progressive rise in *J*_HER_. The enhanced HER performance was ascribed to the porous heterostructured FeP/FeO interface, increased active site exposure, and a notably diminished charge‐transfer resistance of 11 Ω, which enabled swift electron and mass movement. Although FeP/FeO/CC delivers very high *J*_HER_, its relatively large Tafel slope indicates sluggish intrinsic HER kinetics, and the observed surface reconstruction during operation may compromise structural stability and long‐term durability under sustained *J*_HER_ [160].

The CAO/NiS400/CC heterojunction was prepared via a hydrothermal process followed by electrodeposition, resulting in CoAl_2_O_4_ particles intimately embedded within Ni_3_S_2_ on carbon cloth [161]. The SEM image (Figure 11a) shows a fibrous structure uniformly coated with nanoscale CAO and Ni_3_S_2_ domains. EDX lines confirm that Co, Al, O, Ni, and S are distributed uniformly across the structure, which indicates that a well‐defined heterojunction interface has formed (Figure 11b,c). CAO/NiS400/CC has better HER activity in 1 M KOH. It displays a low η_10_ of 25.5 mV ^2^ and η_100_ of 163.2 mV, and a *J*_HER_ of 600 mA/cm^2^ at ‐0.7 V, which is significantly superior to CC, CAO/CC, and NiS400/CC (Figure 11d). The Tafel slope of 20.4 mV/dec (Figure 11e) demonstrates that rapid HER kinetics follow the Volmer–Tafel mechanism. EIS indicates that the charge‐transfer resistance is much lower than in the control samples (Figure 11f). The CAO/NiS400/CC demonstrates competitive HER performance in comparison with many reported bifunctional electrocatalysts, particularly excelling over several systems with inferior HER kinetics. For instance, S–N/NiFe‐LDH/NF has a higher η_10_ of 46 mV and a Tafel slope of 43.4 mV/dec, indicating slower HER kinetics than those of CAO/NiS400/CC (25.5 mV, 20.4 mV/dec) [162]. Mo‐CoP/Co–N–C has weaker HER activity as well, with an η_10_ of 45 mV and a Tafel slope of 40 mV/dec [163], On the other hand, TS‐V_2_CT_x_/CoV_2_O_6_ HN has a higher η_10_ of 32.2 mV and much slower kinetics (75.7 mV/dec) [164]. Also, the CAO/NiS400/CC is very durable, as it maintains stable HER performance for 40 h of continuous operation and after 3000 CV cycles, with almost no decline in activity. The HER mechanism in Figure 11g suggests that the heterojunction interface facilitates water dissociation and hydrogen adsorption, while the conductive carbon cloth ensures efficient electron delivery to active sites. Although CAO/NiS400/CC shows superior performance, the complexity of its preparation method and the feasibility of CAO/NiS400/CC for dissolution and reconstruction during the HER are great barriers limiting long‐term durability. Meanwhile, the roles of metal active sites are not understood [161].

**FIGURE 11:**
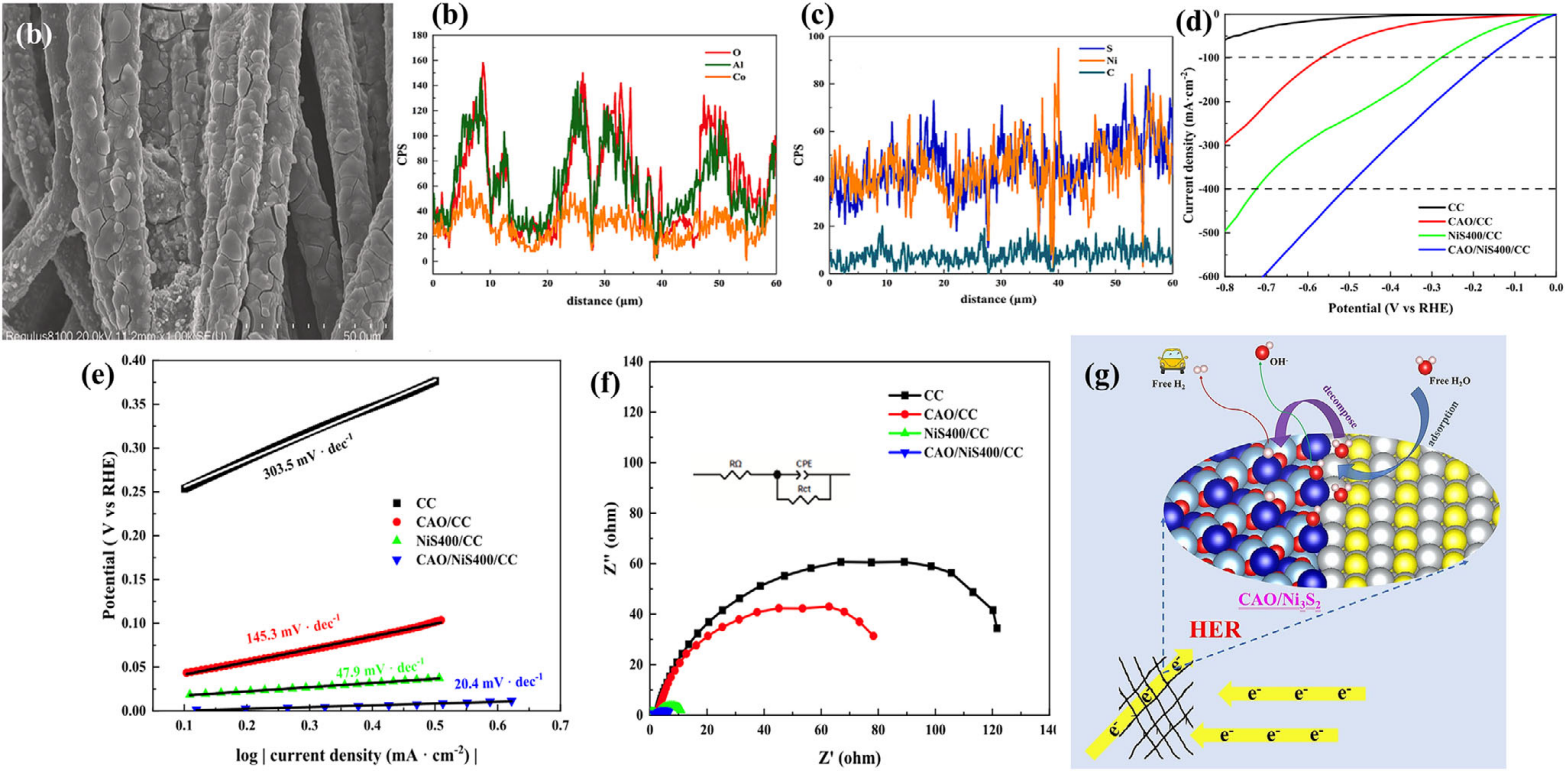
(a) SEM images of CAO/NiS400/CC, (b‐c) EDX lines, (d) LSV, (e) Tafel slope of CAO/NiS400/CC, and (f) EIS Nyquist plots in 1 M KOH compared with others. (g) HER Mechanism. Copyright 2024 Elsevier, Ref. [161].

Wrinkled MoS_2_ nanosheets anchored on carbon cloth (MoS_2_@CC‐H230) were synthesized via the hydrothermal method, yielding a binder‐free electrode with optimized morphology and phase composition, as shown in the SEM images in Figure 12a,b,c [165]. Electrochemical measurements in 1.0 M KOH demonstrate that MoS_2_@CC‐H230 displayed a low η_10_ of 98 ± 3 mV at 10 mA/cm^2^ and a Tafel slope of 108 ± 5 mV/dec. Hence, the HER kinetics were superior compared to those of bulk MoS_2_ and bare carbon cloth (Figure 12d,e). MoS_2_@CC‐H230 has a higher cathodic *J*_HER_ at lower η_10_ than MoS_2_@CC‐H205, MoS_2_@CC‐H250, bulk MoS_2_, and bare carbon cloth. MoS_2_@CC‐H250 exhibits moderate activity, whereas MoS_2_@CC‐H205 performs poorly at higher overpotentials. Bulk MoS_2_ has low HER activity, and carbon cloth is mostly inactive. The Pt benchmark remains superior to all samples, achieving the lowest overpotential and fastest HER kinetics. The polarization behavior further shows that MoS_2_@CC‐H230 delivers a *J*_HER_ of −100 mA/cm^2^ at −0.22 V, approaching the activity trend of the Pt benchmark (Figure 12d). The durability measurement demonstrates that MoS_2_@CC‐H230 displays an almost constant overpotential for 15 h of continuous operation, with only a small polarization shift observed in the initial and final stability measurements (Figure 12f,g). This indicates that it is extremely stable electrochemically under alkaline HER conditions. Although the HER activity is better, MoS_2_@CC‐H230's partial reliance on metastable 1T‐MoS_2_ domains may raise concerns regarding long‐term phase stability under prolonged alkaline operation [165].

**FIGURE 12:**
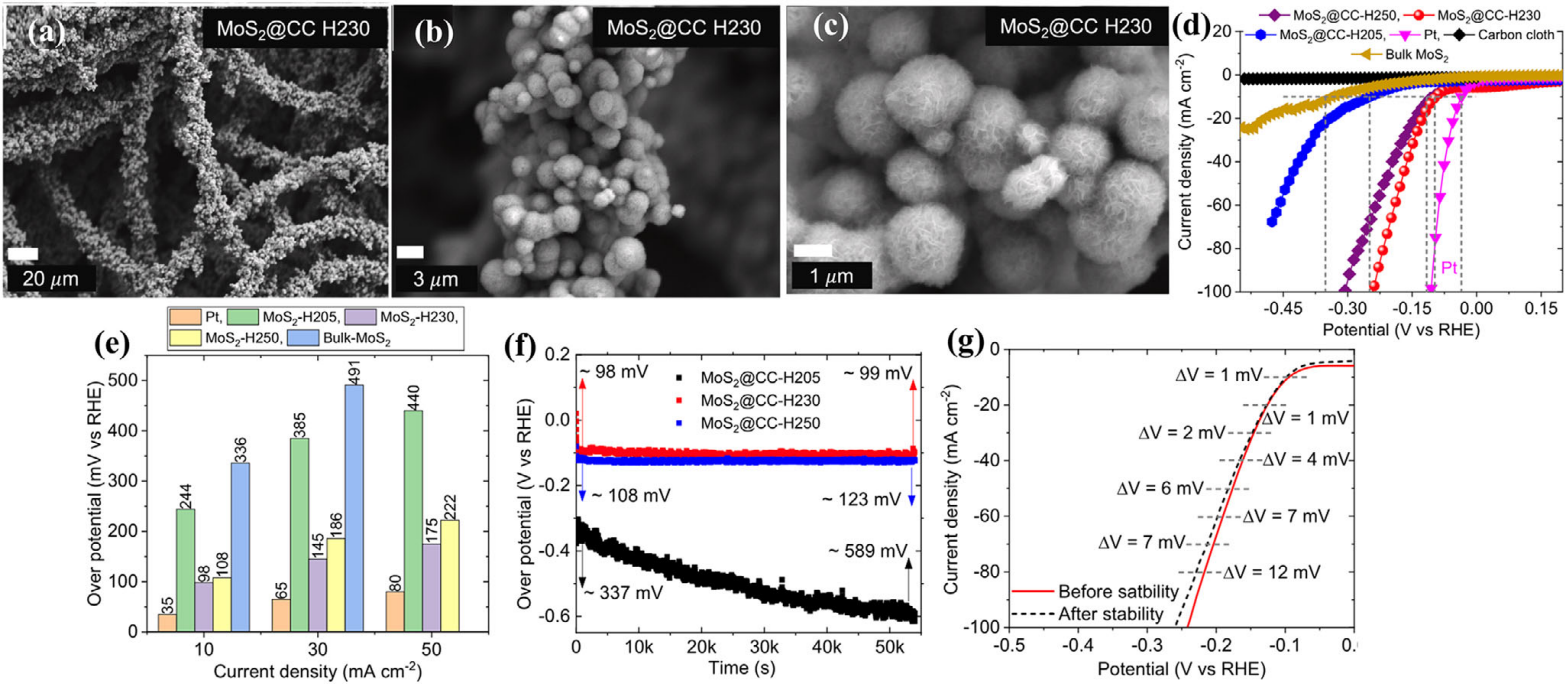
(a–c) SEM images of MoS_2_@CC‐H230. (d) LSV of MoS_2_@CC‐H230 in 1 M KOH compared with other, (e) Comparison of overpotentials as a function of *J*_HER_ for the electrocatalysts, (f) Chronopotentiometry measurements carried out at a *J*_HER_ of 10 mA/cm^2^ over 15 h, (g) LSV measured at a sweep rate of 2.0 mV s^−1^ before and after the 15 h CP measurement. Copyright 2025 ELSEVIER, Ref. [165].

NiO–MoS_2_ was prepared by solvothermal synthesis, thermal treatment, and scalable screen‐printing onto carbon cloth (NiO–MoS_2_/CC) for flexible, freestanding electrodes [166]. The fabrication process creates a well‐integrated NiO–MoS_2_ heterostructure on carbon cloth, providing intimate interfacial contact and improving electron transport between the active phases and conductive substrate. The NiO–MoS_2_/CC demonstrated strong HER activity in 1.0 M KOH, with a *J*_HER_ of −100 mA/cm^2^ at −0.30 V, η_10_ of −159 mV, and a Tafel slope of 116 mV/dec, indicating the Volmer–Heyrovsky mechanism. The NiO–MoS_2_/CC exhibited long‐term stability, maintaining a constant current response with a 7% drop in activity over 40 h. The stable performance is attributed to the robust NiO–MoS_2_ interface, flexible carbon cloth support, and improved structural integrity during prolonged electrolysis. The addition of NiO to the MoS_2_ matrix enhanced HER performance by increasing the interlayer spacing, the density of defect‐rich edge sites, and charge‐transfer resistance (Rct = 2.6 Ω). The XPS, CV‐derived electrochemical surface area analysis, and EIS tests corroborated this. Despite good *J*_HER_, the NiO–MoS_2_/CC has a wide Tafel slope, suggesting slow intrinsic HER kinetics and restricted water‐dissociation efficiency, which limits performance improvement at lower overpotentials and prolonged high‐current operation [166].

A low‐cost ternary composite catalyst composed of fly‐ash‐derived Fe_3_O_4_ magnetic spheres and graphite nanosheets coated on carbon cloth (0.78MA0.32G@CC) was fabricated via ultrasonic dispersion and drop‐casting, forming a conductive and binder‐assisted composite layer [167]. The Fe_3_O_4_/graphite interface provides strong coupling between catalytic oxide sites and the conductive carbon network, improving electron transport and catalyst accessibility. The optimized 0.78MA0.32G@CC had a high *J*_HER_ of −400 mA/cm^2^ at −0.72 V, η_10_ of 175.6 mV, and a small Tafel slope of 45.5 mV/dec in 1.0 M KOH. This suggests that the Volmer–Heyrovsky kinetics were good. The 0.78MA0.32G@CC was very durable, with stable HER operation for 30 h and only small increases in overpotential after 3000 cyclic voltammetry cycles. The excellent durability is attributed to the stable Fe_3_O_4_/graphite composite structure, strong adhesion to the carbon cloth substrate, and conductive graphite network that maintains electrical connectivity during prolonged electrolysis. The improved HER performance was attributed to the catalytically active Fe_3_O_4_ (111) crystal planes and the high electronic conductivity of the graphite nanosheets. As shown by CV and EIS analyses, this made electron transport more efficient, increased the ECSA, and lowered charge‐transfer resistance to 7.97 Ω. However, the relatively high overpotential needed at very high *J*_HER_ shows that Fe_3_O_4_‐based active sites have inherent limitations when it comes to dissociating water, which could lower efficiency in industrial‐scale alkaline HER conditions [167].

### 8.1 Design Principles of Alkaline HER Interface

Unlike acidic HER, catalyst–carbon cloth interactions play an even more critical role in alkaline HER because efficient water dissociation is required before hydrogen evolution. Strong interfacial coupling promotes electron transfer, regulates the adsorption energetics of key intermediates, and provides accessible sites for water activation and subsequent hydrogen adsorption. In addition, the interconnected 3D porous structure of carbon cloth facilitates electrolyte penetration, accelerates hydrogen bubble release, and ensures strong catalyst immobilization, contributing to improved activity and durability. Therefore, interface engineering, including optimization of electronic coupling, surface chemistry, and catalyst anchoring, is a crucial strategy for improving alkaline HER beyond simple catalyst composition modification. Nevertheless, quantitative relationships between interfacial structures, water dissociation kinetics, and catalytic performance remain insufficiently understood, highlighting the need for advanced operando characterization and theoretical investigations.

## 9 Interfacial Interaction of CC with Metals/Nonmetals in Universal pH

As discussed in the previous two sections, the interfacial interaction is important for enhancing HER performance under universal‐pH conditions based on the same fundamental principles. In Table 5, the activity of various catalysts supported on carbon cloth electrode substrates is summarized.

**TABLE 5:** Comparison of the HER performance of CC‐supported metals/nonmetals in different electrolytes.

Catalyst	Preparation method	Electrolyte	η_10_ (mV)	Tafel (mV/dec)	Current density	Stability (h/%)	References
MoS_2_/Mo	Hydrothermal treatment	0.5 M H_2_SO_4_	168	48	∼−100 mA/cm^2^ @ −0.2	17 h	[168]
		1 M PBS	244	94	∼−100 mA/cm^2^ @−0.35	—	
		1 M KOH	184	87	∼−100 mA/cm^2^ @−0.3	—	
IPNTs/CC	Hydrothermal and phosphidation (CVD)	1 M KOH	120	59.5	∼−100 mA/cm^2^ @−0.19	14 h	[169]
		0.5 M H_2_SO_4_	88	35.5	∼−100 mA/cm^2^ @−0.15		
MoP_2_ NS/CC	Hydrothermal growth	0.5 M H_2_SO_4_	58	63.6	∼−250 mA/cm^2^ @−0.15	20 h	[170]
		1 M KOH	67	70	∼−250 mA/cm^2^ @−0.17	25 h	
		1 M PBS	85	98.3	∼−80 mA/cm^2^ @−0.2	20 h	
MoP‐HS@CC	Hydrothermal growth and temperature‐programmed reduction (TPR)	0.5 M H_2_SO_4_	87	61	∼−200 mA/cm^2^ @−0.25	22.22 h	[171]
		1 M KOH	121	64	∼−200 mA/cm^2^ @−0.3	22.22 h	
CoP@CC	Electrochemical deposition and in‐situ thermal phosphorization	0.5 M H_2_SO_4_	131	64	∼−60 mA/cm^2^ @−0.35	27 h	[172]
		1 M KOH	—	70	∼−57 mA/cm^2^ @−0.35	27 h	
Co_2_P@NPC/CC‐800	In situ carbonization–phosphidation synthesis	0.5 M H_2_SO_4_	116	63	∼−50 mA/cm^2^ @−0.15	20 h	[173]
		1 M KOH	129	93	∼−50 mA/cm^2^ @−0.24	20 000 s	
Cu–CoP NRAs/CC	Hydrothermal growth and low‐temperature phosphidation	0.5 M H_2_SO_4_	44	86	∼−50 mA/cm^2^ @−0.18	40 h	[174]
		1 M PBS	137	144	∼−50 mA/cm^2^ @−0.4	40 h	
		1 M KOH	81	102	∼−50 mA/cm^2^ @−0.2	40 h	
MoS_2_/CC‐3	Hydrothermal method	0.5 M H_2_SO_4_	160	50	∼−100 mA/cm^2^ @−0.22	—	[175]
		1 M KOH	188	52	∼−100 mA/cm^2^ @−0.27	—	
		0.5 M PBS	230	84	∼−40 mA/cm^2^ @−0.4	—	
Cu_6_Co_7_/CC	Drop‐casting of a heterometallic POM precursor onto carbon cloth	0.2 M PBS	390	96	∼−100 mA/cm^2^ @−0.42	4 h	[176]
CCC‐PAN	Carbonization, PAN treatment, and secondary annealing	1 M NaOH	233	135	∼−100 mA/cm^2^ @−0.4	>15 h	[177]
NiCoP/S/CC	Phosphorization and controlled sulfurization	1 M KOH	76	65	∼−400 mA/cm^2^ @−0.25	—	[178]
		0.5 M H_2_SO_4_	90	52	∼−300 mA/cm^2^ @−0.28		
CDs/Pt‐PANI	Electrolytic etching method, electrodeposition	0.5 M H_2_SO_4_	30	41.7	—	20 h	[179]
		1 M PBS	134	58	—	—	
		1 M KOH	56	93	—	—	
V, N‐CoP/CC	Hydrothermal synthesis and phosphorization	0.5 M H_2_SO_4_	81	59	∼−100 mA/cm^2^ @−0.23	50 h	[180]
		1 M PBS	146	88	∼−50 mA/cm^2^ @−0.35	50 h	
		1 M KOH	57	51	∼−100 mA/cm^2^ @−0.24	50 h	
NiFeP/CoP/CC	Electrodeposition, hydrothermal processing, and phosphorus (P) doping	1 M KOH	96.38	70.67	∼−60 mA/cm^2^ @−0.15	24 h	[181]
		0.5 M H_2_SO_4_	78.8	63.21	∼−60 mA/cm^2^ @−0.125		
Ru_2_P–Ni_2_P/CC	Phosphidation	0.5 M H_2_SO_4_	46	41	∼−50 mA/cm^2^ @−0.1	50 h	[182]
		1 M PBS	51	45	∼−50 mA/cm^2^ @−0.15		
		1 M KOH	47	43	∼−50 mA/cm^2^ @−0.15		
Zn_0_._64_–CoP/Co_2_P@CC	Hydrothermal and phosphorization route	0.5 M H_2_SO_4_	81	69	∼−40 mA/cm^2^ @−0.17	23 h	[183]
		1 M KOH	95	87	∼−70 mA/cm^2^ @−0.25	23 h	
P‐MoS_2_@CoP/CC	Hydrothermal and phosphorization process	1 M KOH	64	51.2	∼−120 mA/cm^2^ @−0.15	—	[184]
		0.5 M H_2_SO_4_	72	59.7	∼−80 mA/cm^2^ @−0.14	80 h	
MnS–MoS_2_/CC	Hydrothermal and sulfidation method	0.5 M H_2_SO_4_	141	112	∼−100 mA/cm^2^ @−0.4	24 h	[185]
		1 M PBS	221	214	∼−100 mA/cm^2^ @−1.0		
		1 M KOH	54	71	∼−100 mA/cm^2^ @−0.28		
Mo_2_C/Mo_2_N‐800/CC	Hydrothermal method	0.5 M H_2_SO_4_	96	60	∼−100 mA/cm^2^ @−0.17	12 h	[186]
		1 M KOH	54	48	∼−500 mA/cm^2^ @−0.3		
		1 M PBS	73	59	∼−100 mA/cm^2^ @−0.17		
Ni_0_._62_Co_0_._35_Fe_0_._03_)_3_Se_4_/CC	Hydrothermal method	0.5 M H_2_SO_4_	50	111	∼−550 mA/cm^2^ @−0.6	20 h	[187]
		1 M KOH	87	122.6	∼−240 mA/cm^2^ @−0.5		
CC@W‐NR	Hydrothermal method	1 M KOH	151	82	∼−130 mA/cm^2^ @−0.3	—	[188]
		0.5 M H_2_SO_4_	85	76	∼−250 mA/cm^2^ @−0.28	12 h	
NiCo_2_S_4_/ReS_2_/CC	Hydrothermal process and sulfuration	1 M KOH	85	78.3	∼−200 mA/cm^2^ @−0.2	15 h	[189]
		0.5 M H_2_SO_4_	126	67.8	∼−200 mA/cm^2^ @−0.24		
CoSe_2_/CC	Hydrothermal formation and selenization	1 M KOH	98	83	∼−100 mA/cm^2^ @−0.22	12 h	[190]
		0.5 M H_2_SO_4_	52	38	∼−100 mA/cm^2^ @−0.1	12 h	
Mo_2_N‐MoC (MCN)	Carbonization and nitridation technique	0.5 M H_2_SO_4_	193	68	∼−100 mA/cm^2^ @−0.2	80 h	[191]
		1 M PBS	376	84	∼−120 mA/cm^2^ @−0.4		
		1 M KOH	201	48	∼−100 mA/cm^2^ @−0.2		
50% 1T/2H‐WS_2_/N‐rGO/CC, as WGC	Hydrothermal and solvothermal synthesis	0.5 M H_2_SO_4_	21.13	29.5	∼−65 mA/cm^2^ @−0.1	8 h	[192]
		1 M KOH	80.35	137.02	∼−65 mA/cm^2^ @−0.5		
CoSe_2_–CoP/CC	Hydrothermal, phosphorization, and selenization route.	1 M KOH	51	52	∼−200 mA/cm^2^ @−0.25	70 h	[193]
		0.5 M H_2_SO_4_	38	43	∼−200 mA/cm^2^ @−0.26	100 h	
CoP/Co_3_O_4_/CC	Hydrothermal, calcination, and electrodeposition methods	1 M PBS	179	251	∼−100 mA/cm^2^ @−0.60	—	[194]
		1 M KOH	58	116	∼−200 mA/cm^2^ @−0.42	32 h	

A three‐dimensional macroporous molybdenum disulfide thin film, directly grown on molybdenum foil (MoS_2_/Mo), was fabricated via an in situ hydrothermal treatment using thiourea as the sulfur source [168]. The binder‐free, directly grown MoS_2_/Mo outperformed Mo foil, and it was close to Pt/C. In 0.5 M H_2_SO_4_, driven by a synergistic effect and a short electron transport pathway with improved mechanical adhesion, the MoS_2_/Mo delivered *J*_HER_ of ∼−100 mA/cm^2^ at −0.20 V, η_10_ of 168 mV, η_100_ of 215 mV, and a Tafel slope of ∼48 mV/dec indicates that HER proceeds via the electrochemical desorption (Heyrovsky) step as the rate‐limiting process. In 1.0 M PBS, MoS_2_/Mo delivered *J*_HER_ of ∼−100 mA/cm^2^ at −0.35 V with η_10_ = 244 mV and a Tafel slope of ∼94 mV/dec. In 1.0 M KOH, MoS_2_/Mo delivered *J*_HER_ of −100 mA/cm^2^ at −0.30 V, with η_10_ of 184 mV, η_100_ of 275 mV, and a Tafel slope of ∼87 mV/dec. The HER performance was attributed to the 3D macroporous structure, abundant MoS_2_ edge sites, and direct MoS_2_–Mo electrical coupling accelerates interfacial charge transfer and contributes to enhanced reaction kinetics, while good durability was confirmed by stable operation for 17 h in acidic media and showing negligible degradation after 1000 cyclic voltammetry cycles in neutral and alkaline electrolytes. The higher stability is attributed to the ability of MoS_2_ films to preserve their composition after the durability test. Despite its improved performance over Mo foil, the MoS_2_/Mo still requires relatively high overpotentials and exhibits slower HER kinetics in neutral and alkaline media, reflecting intrinsic limitations of MoS_2_ that restrict efficiency at higher *J_HER_* [168].

Phosphorization of iron hydroxide nanotube precursors produced binder‐free, flexible iron phosphide nanotubes supported on carbon cloth (IPNTs/CC) [169]. In 0.5 M H_2_SO_4_ and 1.0 M KOH, IPNTs/CC displayed *J*_HER_ of ∼−100 mA/cm^2^ at −0.15 and −0.19 V, η_10_ of 88 and 120 mV, and Tafel slopes of 35.5 and 59.5 mV/dec, respectively. IPNTs/CC exhibit faster HER kinetics and lower overpotentials than other FeP‐based catalysts, attributed to the 1D architecture and high surface area that facilitate electrolyte penetration, while the in situ formed IPNTs promote water dissociation and accelerate interfacial charge transfer. It approaches commercial Pt/C's performance in acidic conditions but falls short. The one‐dimensional nanotube shape and the in situ‐produced iron oxide/phosphate surface layer allow water dissociation and charge transfer, boosting activity. Stable operation for 14 h in both electrolytes demonstrated durability, meaning preserving its chemical composition without corrosion or catalyst detachment. Although it shows high activity, its performance is slightly less than that of Pt/C at lower potentials. Moreover, its stability is limited, which could restrict its use in industrial applications over time [169].

A self‐standing MoP_2_ nanosheet array supported on carbon cloth (MoP_2_ NS/CC) was prepared by topotactic phosphidation of a MoS_2_ nanosheet array precursor grown hydrothermally on carbon cloth [170]. SEM images (Figure 13a,b) show that vertically aligned MoP_2_ nanosheets uniformly cover the carbon cloth fibers, while SEM–EDX elemental mapping confirms the homogeneous distribution of Mo and P and complete conversion from the MoS_2_ precursor (Figure 13c). The binder‐free architecture, 2D structure, and heteroatom incorporation together enhance the exposed active surface area, improve electrolyte wettability, and reduce interfacial charge‐transfer resistance, thereby accelerating electron transport and promoting HER kinetics. In 0.5 M H_2_SO_4_, MoP_2_ NS/CC has much better HER activity than MoS_2_ NS/CC and bare carbon cloth. It displays an η_10_ of 58 mV, a Tafel slope of 63.6 mV/dec, and a high *J*_HER_ of −250 mA/cm^2^ at −0.15 V, which is close to the performance of Pt/CC (Figure 13d), indicating that the HER proceeds via a Volmer–Heyrovsky mechanism, where the electrochemical desorption step (Heyrovsky reaction) is the main step. The MoP_2_ NS/CC is highly durable in acidic environments. It can maintain a JHER of 20 mA/cm^2^ for more than 20 h of continuous electrolysis. Its morphology and structure remain unchanged after 2000 cyclic voltammetry cycles (Figure 13e). In 1.0 M KOH and 1.0 M PBS, MoP_2_NS/CC exhibited η_10_ values of 67 and 85 mV, with Tafel slopes of 70 and 98.3 mV/dec, respectively (Figure 13f,g). The MoP_2_NS/CC exhibits excellent stability, showing a slight decrease in *J*_HER_ after 2000 CV cycles and maintaining stable electrolysis for at least 25 h in 1.0 M KOH and 20 h in 1.0 M PBS (Figure 13h,i). However, a multistep reaction is a significant barrier [170]. It exhibits excellent chemical stability without catalyst detachment and provides strong corrosion resistance.

**FIGURE 13:**
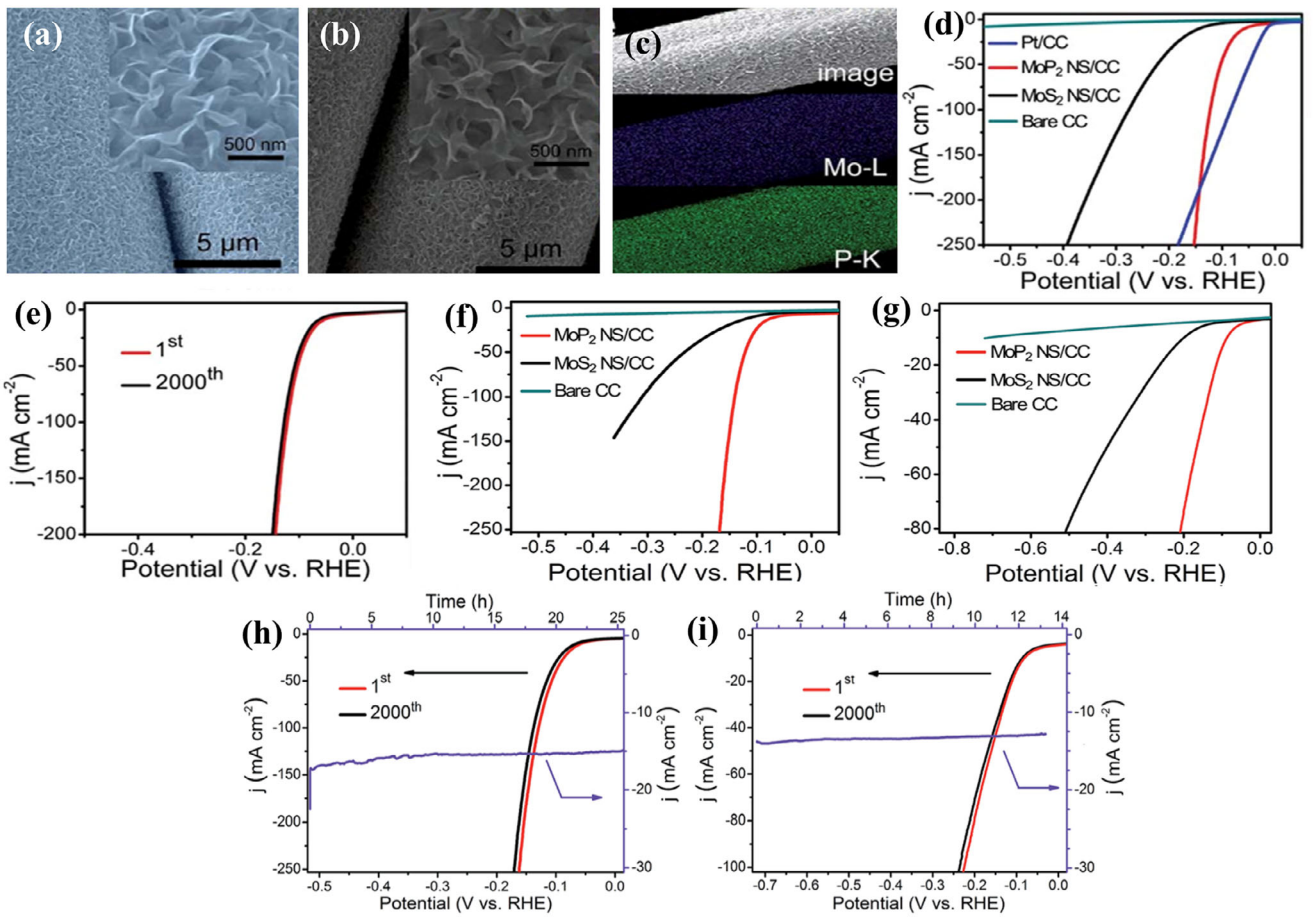
(a) SEM images of MoS_2_ NS/CC, (b) SEM images of MoP_2_ NS/CC, (c) EDX elemental mapping images of Mo and P for MoP_2_ NS/CC, (d) LSV of MoP_2_ NS/CC, MoS_2_ NS/CC, and Pt/C on CC and bare CC in 0.5 M H_2_SO_4_ with a scan rate of 5 mV/s, (e) LSV of MoP_2_ NS/CC before and after 2000 CV cycles, LSV of MoP_2_ NS/CC, MoS_2_ NS/CC and bare CC in (f) 1.0 KOH M, (g) and PBS at a scan rate of 5 mVs^−1^. (h) LSVs of MoP_2_ NS/CC before and after 2000 CV cycles, and (i) the time‐dependent *J*_HER_ curves of MoP_2_ NS/CC under static overpotential in 1.0 M KOH and PBS. Copyright 2025 ELSEVIER, Ref. [170].

Mesoporous molybdenum phosphide hemispheres directly grown on carbon cloth (MoP‐HS@CC) were synthesized via hydrothermal growth of a molybdenum phosphate precursor, followed by temperature‐programmed reduction, thereby forming a binder‐free, self‐supported HER electrode [171]. In 0.5 M H_2_SO_4_ and 1.0 M KOH, the MoP‐HS@CC displayed a high *J*_HER_ of −200 mA/cm^2^ at −0.25 and −0.30 V, η_10_ of 87 and 121 mV, and η_100_ of 195 and 223 mV, with Tafel slopes of ∼61 and ∼64 mV/dec, respectively. Compared to MoP microspheres supported on carbon cloth, MoP‐HS@CC has lower overpotentials and faster HER kinetics. However, it is still not as good as commercial Pt/C, particularly at low overpotentials. The enhanced activity is attributed to the mesoporous hemispherical structure, the larger ECSA, and the direct electrical connection to the carbon cloth, which facilitates electrolyte accessibility, exposes more catalytic sites, and accelerates interfacial electron transfer. The MoP‐HS@CC shows good stability. In acidic conditions, the overpotential only increased by 10 mV after 3000 CV cycles, while maintaining a *J*_HER_ of about 40 mA/cm^2^ for 41.67 h. In alkaline media, ∼40 mA/cm^2^ was sustained for 22.22 h with minimal change, due to the strong MoP–carbon cloth coupling and robust structural integrity that suppress catalyst detachment and corrosion. However, it shows relatively high overpotential at large *J*_HER_ and limited long‐term stability evaluation [171].

Carbon cloth supported cobalt phosphide nanosheets (CoP@CC) were synthesized via electrochemical deposition of Co(OH)_2_ followed by in situ phosphorization, forming a binder‐free HER [172]. In 0.5 M H_2_SO_4_ and 1.0 M KOH, the CoP@CC delivered a *J*_HER_ of −60 and −57 mA/cm^2^ at −0.35 V, an η_10_ of 131 mV in acidic media, and a low onset potential of −86 mV at −1 mA/cm^2^ in alkaline media, with Tafel slopes of ∼64 and ∼70 mV/dec, respectively. Compared with Co_3_O_4_@CC, Co(OH)_2_@CC, and CoP powder, CoP@CC shows lower overpotentials and faster HER kinetics, but it remains inferior to commercial Pt/C at low overpotentials. The increased activity is due to the vertically aligned nanosheet architecture and direct growth on CC, which makes it easier for charge to move and for active sites to be reached by providing abundant exposed edge sites, improved electrolyte diffusion, and efficient interfacial electron transport. Good durability is demonstrated by stable operation for more than 100 000 s and 27 h in 0.5 M H_2_SO_4_ and 1.0 M KOH, respectively, due to strong CoP–carbon cloth interaction, which prevents catalyst detachment and maintains structural integrity during electrolysis. However, compared to Pt‐based catalysts, larger current densities still require higher overpotentials, which limit operational efficiency [172].

Carbon cloth‐supported Co_2_P quantum dots embedded in N‐, P‐dual‐doped carbon (Co_2_P@NPC/CC) were fabricated via in situ carbonization of cobalt‐phytic acid‐polyaniline precursors, forming a flexible, binder‐free HER, and enabling intimate integration between N,P‐dual‐doped Co_2_P quantum dots and the conductive carbon matrix, creating a robust catalyst–carbon cloth interface with shortened electron transport pathways and strong interfacial coupling [173]

In 0.5 M H_2_SO_4_ and 1.0 M KOH, the Co_2_P@NPC/CC‐800 delivered a *J*_HER_ of −50 mA/cm^2^ at −0.15 V and −50 mA/cm^2^ at −0.24 V, respectively, with η_10_ values of 116 mV and 129 mV, and Tafel slopes of ∼63 and ∼93 mV/dec, respectively, indicative of Volmer–Heyrovsky HER kinetics. Co_2_P@NPC/CC‐800 exhibited lower overpotentials and faster HER kinetics than NPC, CC, and other Co_2_P samples prepared at different temperatures, and it was also inferior to commercial Pt/C, particularly at low overpotentials. The improved activity is due to the abundant Co_2_P/carbon interfaces and ultrafine quantum dot size, which maximize exposed catalytic sites. At the same time, dual doping modulates the electronic structure of carbon and Co_2_P, optimizing hydrogen adsorption and accelerating interfacial charge transfer. It showed excellent stability in both acidic and alkaline media, with operation for 20 h at 120 mV in acid and 20 000 s at 190 mV in alkaline conditions, attributed to the strong confinement effect of the carbon matrix, which prevents Co_2_P quantum dot aggregation and detachment, while the robust conductive framework maintains structural integrity. However, higher overpotentials are still required to achieve high current densities in alkaline media compared with Pt‐based catalysts, thereby limiting efficiency under industrial operating conditions [173].

Self‐supported Cu‐doped cobalt phosphide nanorod arrays on carbon cloth (Cu–CoP NRAs/CC) were fabricated via hydrothermal growth of CuCo‐LDH precursors, followed by low‐temperature phosphidation, thereby forming a binder‐free and strongly coupled Cu–CoP/carbon cloth interface, enabling direct electron transport and robust mechanical adhesion [174]. In 0.5 M H_2_SO_4_, 1.0 M PBS, and 1.0 M KOH, Cu–CoP NRAs/CC displayed *J*_HER_ of −50 mA/cm^2^ at −0.18, −0.40, and −0.20 V, respectively, a η_10_ of 44, 137, and 81 mV, and a Tafel slopes of ∼86, ∼144, and ∼102 mV/dec, respectively, indicating a Volmer–Heyrovsky reaction, as the rating determine step. The increased activity is driven by Cu‐induced lattice distortions and defect formation, which increase the number of active sites and facilitate interfacial charge transfer; also, the aligned nanorod array improves ion access and gas release during HER. In contrast, it demonstrates exceptional durability, with steady operation for 40 h in acidic, neutral, and alkaline electrolytes, attributed to the stable Cu‐CoP framework and strong interaction with carbon cloth, which suppress catalyst detachment, reconstruction, and corrosion during electrochemical operation. Compared to undoped CoP NRAs/CC and Cu_3_P nanorods, Cu–CoP NRAs/CC has much lower overpotentials and faster HER kinetics. However, it remains less efficient compared to commercial Pt/C, especially at low overpotentials [174].

MoS_2_/CC nanoflowers directly grown on carbon cloth were synthesized via hydrothermal growth, resulting in a hierarchical, self‐supported structure that eliminates binder‐induced resistance and shortens the electron transport pathway between active MoS_2_ sites and the conductive substrate for HER [175]. The MoS_2_/CC displayed *J*_HER_ values of −100 mA/cm^2^ at −0.22 V, −100 mA/cm^2^ at −0.27 V, and −40 mA/cm^2^ at −0.40 V in 0.5 M H_2_SO_4_, 1.0 M KOH, and 0.5 M PBS, respectively. The MoS_2_/CC‐3 displayed η_10_ values of 160, 188, and 230 mV^2^, with Tafel slopes of ∼50, ∼52, and ∼84 mV/dec in 0.5 M H_2_SO_4_, 1.0 M KOH, and 0.5 M PBS, respectively. The optimized MoS_2_/CC exhibits lower overpotentials and faster HER kinetics than bare carbon cloth and the mixed MoS_2_/CC. However, it is still not quite as efficient as commercial Pt/C, especially at low overpotentials. The increased activity is due to the hierarchical nanoflower structure, wider interlayer spacing, more defects, and improved electrical conductivity of the carbon cloth substrate, which expose more accessible MoS_2_ edge sites, enhance electrolyte penetration/ion diffusion, and promote interfacial charge transfer during HER. In contrast, good durability is confirmed by stable operation after 2000 CV cycles and prolonged electrolysis under acidic and neutral conditions, owing to the preserved hierarchical structure and strong catalyst–carbon cloth adhesion without peeling during hydrogen evolution. However, the HER activity is still constrained by the poor intrinsic conductivity and limited active sites of MoS_2_ [175].

A mixed cobalt–copper polyoxometalate deposited on carbon cloth (Cu_6_Co_7_/CC) was prepared via direct integration of the heterometallic POM precursor with the conductive carbon cloth surface by drop‐casting, yielding a noble‐metal‐free electrode that enables forming a self‐supported electrode with short and efficient electron transport pathways [176]. In 0.2 M PBS, the Cu_6_Co_7_/CC delivered a *J*_HER_ of −100 mA/cm^2^ at −0.42 V, η_10_ of 390 mV and η_100_ of 430 mV, with a Tafel slope of ∼96 mV/dec, indicating a Volmer–Heyrovsky reaction pathway. Compared with bare carbon cloth and the Co–P alloy detailed in the same paper, Cu_6_Co_7_/CC exhibits markedly enhanced HER activity and accelerated kinetics, yet remains less effective than commercial Pt/C, which operates at considerably lower overpotentials. The improved performance is due to the polyoxometalate functioning as a precatalyst that transforms in situ into catalytically active low‐valence W‐based species during the HER; the catalyst/carbon cloth interface promotes electron transfer and provides active species; and the heterometallic composition eases electronic interaction between metal centers, optimizing catalytic reaction intermediates. The Cu_6_Co_7_/CC is also very stable in operation, maintaining a steady current for more than 4 h of continuous electrolysis with a Faradaic efficiency of 98%, due to strong interaction between the converted active phase and the carbon cloth, which can maintain catalyst dispersion, suppress active material loss, with preserveing electrode composition during electrolysis. However, the relatively high overpotential required to achieve practical *J*_HER_ limits its energy efficiency compared with state‐of‐the‐art HER catalysts [176].

Nitrogen‐doped carbonized cotton cloth (CCC‐PAN) was fabricated via carbonization of cotton cloth followed by polyacrylonitrile (PAN) treatment and secondary annealing, yielding a flexible, binder‐free, providing a continuous conductive network and strong structural integrity of metal‐free HER [177]. In 1.0 M NaOH, the CCC‐PAN delivered a *J*_HER_ of −100 mA/cm^2^ at −0.40 V, a η_10_ of 233 mV, and a Tafel slope of ∼135 mV/dec. Compared with untreated carbonized cotton cloth (CCC), CCC‐PAN exhibits a much lower overpotential and faster HER kinetics due to nitrogen doping. The improved activity is due to more pyridinic and pyrrolic nitrogen species, which change the electronic structure of carbon and create more active sites by modifying local charge density, improving electron donation ability, and optimizing hydrogen adsorption/desorption interactions at the carbon surface. The durability and flexibility are further supported by consistent performance after 2000 CV cycles, extended chronoamperometric testing (>15 h), and minimal degradation under mechanical bending related to strong interfacial bonding within the carbon framework and resistance to structural cracking. However, the relatively large Tafel slope and high overpotential required to achieve a large *J*_HER_ limit its efficiency compared with state‐of‐the‐art metal‐based HER catalysts [177].

Carbon cloth supported nickel‐cobalt phosphosulfide nanoneedles (NiCoP/S/CC) were fabricated via in situ phosphorization followed by controlled sulfurization of NiCo_2_O_4_ nanoneedle precursors, forming a binder‐free HER [178]. The sequential anion conversion process preserves the 1‐D morphology with a strongly coupled catalyst–substrate interface, rapid electron transport, and robust mechanical adhesion. In 1.0 M KOH and 0.5 M H_2_SO_4_, NiCoP/S/CC displayed *J*_HER_ around −400 mA/cm^2^ at −0.25 V and −300 mA/cm^2^ at −0.28 V, a η_10_ of 76 mV and η_10_ of 90 mV, and Tafel slopes of 65 mV/dec and 57 mV/dec, respectively, which suggests a Volmer–Heyrovsky HER mechanism. NiCoP/S/CC exhibits lower overpotentials and faster kinetics than NiCoP/CC, NiCoS/CC, NiCoP/S‐2/CC, and other non‐noble‐metal catalysts studied. However, it is still not as good as commercial Pt/C, especially at low overpotentials. The improved performance is due to the dual‐anion modulation that regulates the electronic structure of Ni and Co active centers, optimizes hydrogen adsorption/desorption kinetics, 1D provides abundant exposed active sites, efficient electrolyte penetration, and shortened ion/electron diffusion pathways. Excellent durability is confirmed by negligible degradation after 1000 CV cycles, and stable long‐term electrolysis in both electrolytes originates from the strong NiCoP/S–carbon cloth interface and the mechanically robust nanoneedle array, which prevent catalyst detachment, maintain structural integrity, and resist surface reconstruction. However, despite its high activity at large *J*_HER_, it requires higher operating potentials than Pt‐based catalysts, which may limit overall energy efficiency in practical applications [178].

Carbon cloth supported on polyaniline and carbon dots (CDs/Pt‐PANI) nanosheets significantly enhanced HER in different electrolytes. Still, the use of Pt diminished the effect of the carbon cloth working electrode [179].

Vanadium‐ and nitrogen‐co‐doped cobalt phosphide nanoleaf arrays supported on carbon cloth (V, N‐CoP/CC) were synthesized via a two‐step hydrothermal phosphorization route to obtain a binder‐free catalyst that can preserve the interconnected nanoleaf architecture while establishing a strongly coupled V,N‐CoP/carbon cloth interface and short electron transport pathways and a firm, pH‐universal HER catalyst [180]. In 0.5 M H_2_SO_4_, 1.0 M PBS, and 1.0 M KOH, the V,N‐CoP/CC delivered *J*_HER_ of −100 mA/cm^2^ at −0.23 V, −50 mA/cm^2^ at −0.35 V, and −100 mA/cm^2^ at −0.24 V, respectively. The V,N‐CoP/CC showed η_10_ values of 81, 146, and 57 mV, with corresponding Tafel slopes of ∼59, ∼88, and ∼51 mV/dec, respectively. V,N‐CoP/CC has much better activity and faster kinetics than pure CoP/CC and V‐CoP/CC, but it is still not as good as commercial Pt/C at low overpotentials. The improved performance is due to synergistic V and N co‐doping, which alters the electronic structure, increases the ECSA, and reduces the charge‐transfer resistance by optimizing hydrogen adsorption, generating additional exposed active sites, and accelerating interfacial electron transfer via conductive carbon. The V,N‐CoP/CC is also durable for a long time, with almost no performance loss after 1000 CV cycles and stable operation for more than 50 h in acidic, neutral, and alkaline electrolytes. The robust V,N‐CoP–carbon cloth interface with a stable nanoleaf array suppresses catalyst detachment and resists corrosion during HER operation. However, despite its excellent activity, the catalyst still requires higher overpotentials than Pt/C and exhibits slower kinetics in neutral media, which may limit its efficiency in practical neutral‐pH systems [180].

NiFeP nanoflakes composited with CoP on carbon cloth (NiFeP/CoP/CC) were prepared via electrodeposition, hydrothermal treatment, and high‐temperature phosphorization, enabling the in situ formation of a hierarchical heterostructure anchored onto carbon cloth, creating intimate heterointerfacial contact and strong electrical coupling with short electron transport pathways [181]. The NiFeP/CoP/CC displayed *J*_HER_ values of −60 mA/cm^2^ at −0.15 V and −0.125 V in 1.0 M KOH and 0.5 M H_2_SO_4_, respectively. The NiFeP/CoP/CC also delivered η_10_ values of 96.38 mV (alkaline) and 78.8 mV (acidic), with Tafel slopes of 70.67 and 63.2 mV/dec, indicating Volmer–Heyrovsky HER kinetics. The NiFeP/CoP/CC has the lowest overpotentials and the highest current densities in both acidic and alkaline media compared to Co/CC, NiFeLDH/Co/CC, NiFeP/CC, and CoP/CC. This demonstrates that it exhibits superior HER kinetics due to synergistic effects between NiFeP and CoP at the interface; however, its performance remains inferior to commercial Pt/C at low overpotentials. EIS analysis indicated that the accelerated HER kinetics were due to the synergistic heterointerface between the NiFeP/CoP interlayer, which induces interfacial charge redistribution, optimizes the electronic structure, lowers the charge‐transfer resistance with abundant active sites, and facilitates electrolyte penetration. It enhances the ECSA and accelerates charge transfer. The electrocatalyst maintained HER performance for over 24 h in acidic and alkaline electrolytes, where the robust NiFeP/CoP heterointerface and strong catalyst adhesion suppress catalyst detachment and resist structural degradation, with efficient electron transport during electrolysis. However, multiple reaction steps and low activity are major barriers [181].

Ru_2_P NPs‐decorated Ni_2_P nanosheets grown on carbon cloth (Ru_2_P–Ni_2_P/CC) were prepared by spraying RuCl_3_ onto Ni(OH)_2_/CC, followed by phosphidation, which enables a well‐coupled Ru_2_P/Ni_2_P heterointerface on carbon cloth, enabling efficient electron transport and strong catalyst adhesion for a pH‐universal HER catalyst [182]. In 0.5 M H_2_SO_4_, 1.0 M PBS, and 1.0 M KOH, the Ru_2_P–Ni_2_P/CC delivered *J*_HER_ of −50 mA/cm^2^ at −0.10 V, −50 mA/cm^2^ at −0.15 V, and −50 mA/cm^2^ at −0.15 V, respectively, ultralow η_10_ of 46, 51, and 47 mV, respectively, with corresponding Tafel slopes of ∼41, ∼45, and ∼43 mV/dec, indicating accelerated HER kinetics across a wide pH range. Compared with Ni_2_P/CC and Ru_2_P/CC (and approaching Pt/C, particularly in alkaline/neutral media), Ru_2_P–Ni_2_P/CC exhibits lower overpotentials and faster kinetics due to the Ru_2_P–Ni_2_P heterointerface, which promotes charge redistribution, optimizes H adsorption by increasing the number of accessible active sites, and reduces charge‐transfer resistance. Good durability was confirmed by 50 h of chronoamperometric stability in acidic, alkaline, and neutral electrolytes, due to a strong interface that suppresses catalyst detachment and maintains structural integrity during HER. However, despite Pt‐like kinetics, it remains slightly inferior to Pt/C at low overpotentials and relies on Ru‐containing phases, which may affect cost‐scalability [182].

Zn‐doped CoP/Co_2_P nanowire arrays supported on carbon cloth (Zn–CoP/Co_2_P@CC) as a binder‐free HER catalyst were synthesized via hydrothermal growth of Zn‐doped Co(OH)F nanowire precursors, followed by phosphorization, which preserves the nanowire architecture and forms a strongly coupled Zn–CoP/Co_2_P heterointerface on carbon cloth for efficient electron transport [183]. In 0.5 M H_2_SO_4_ and 1.0 M KOH, the optimized Zn_0_._64_–CoP/Co_2_P@CC delivered *J*_HER_ of −40 mA/cm^2^ at −0.17 V and −70 mA/cm^2^ at −0.25 V, respectively, a η_10_ of 81 and 95 mV, with Tafel slopes of 69 and 87 mV/dec, respectively, suggesting a Volmer–Heyrovsky HER mechanism with enhanced interfacial reaction kinetics. Compared with undoped CoP/Co_2_P@CC and other Zn‐doped variants, Zn_0.64_–CoP/Co_2_P@CC exhibits the lowest overpotentials and fastest HER kinetics, although it remains inferior to commercial Pt/C at low overpotentials. The increased activity is due to Zn‐induced electronic modulation, the porous nanowire array architecture, and the strong electrical coupling with carbon cloth, which promotes interfacial charge transfer, optimizes H adsorption, and exposes more active sites. Good durability is demonstrated by its performance for more than 23 h in both acidic and alkaline electrolytes, owing to the robust catalyst–carbon cloth interface, which suppresses catalyst detachment and preserves structural integrity. However, performance is highly sensitive to the Zn doping level, with non‐optimal doping reducing activity [183].

P‐doped MoS_2_@CoP nanoarrays grown in situ on carbon cloth (P‐MoS_2_@CoP/CC) were synthesized through a controlled multistep process that included growing Co(OH)F nanowires, depositing MoS_2_, and then phosphorizing the material to create a needle‐like heterostructured architecture with a strongly coupled P‐MoS_2_/CoP heterointerface on carbon cloth, enabling efficient electron transport and robust catalyst adhesion [184]. The SEM images show nanoarrays that are densely packed and aligned vertically (Figure 14a), and the SAED patterns show that CoP and MoS_2_ are crystalline (Figure 14b). HAADF‐TEM imaging and corresponding elemental mappings demonstrate uniform coating of P‐MoS_2_ on CoP nanoarrays with homogeneous distributions of Co, Mo, P, and S, indicating intimate interfacial coupling (Figure 14c). Electrochemical evaluation demonstrates excellent HER activity across a wide pH range, with a low η_10_ of 64 and 72 mV at 10 mA/cm^2^ in 1.0 M KOH and 0.5 M H_2_SO_4_, respectively (Figure 14d,f). P‐MoS_2_@CoP also displayed a *J*_HER_ of ∼−120 mA/cm^2^ at −0.15 V and ∼−80 mA/cm^2^ at −0.14 V with corresponding Tafel slopes of 51.2 and 59.7 in 1.0 M KOH and 0.5 M H_2_SO_4_, respectively, indicating a Volmer–Heyrovsky HER mechanism with rapid electrochemical desorption kinetics, attributed to the heterointerface, which promotes interfacial charge redistribution, optimizes H adsorption, and exposes abundant active sites. (Figure 14e). Potential‐dependent turnover frequency analysis further confirms the enhanced intrinsic activity of P‐MoS_2_@CoP compared with CoP and P‐MoS_2_ counterparts (Figure 14g). Durability tests show that it is very stable. It can handle a constant *J*_HER_ of −100 mA/cm^2^ without significant degradation during long‐term electrolysis in an alkaline solution. It can maintain the same *J*_HER_ for up to 80 h in acidic electrolyte, attributed to the heterointerface and strong catalyst–carbon cloth interaction suppressing catalyst detachment and preserving morphology. However, a multistep reaction is a significant barrier [184].

**FIGURE 14:**
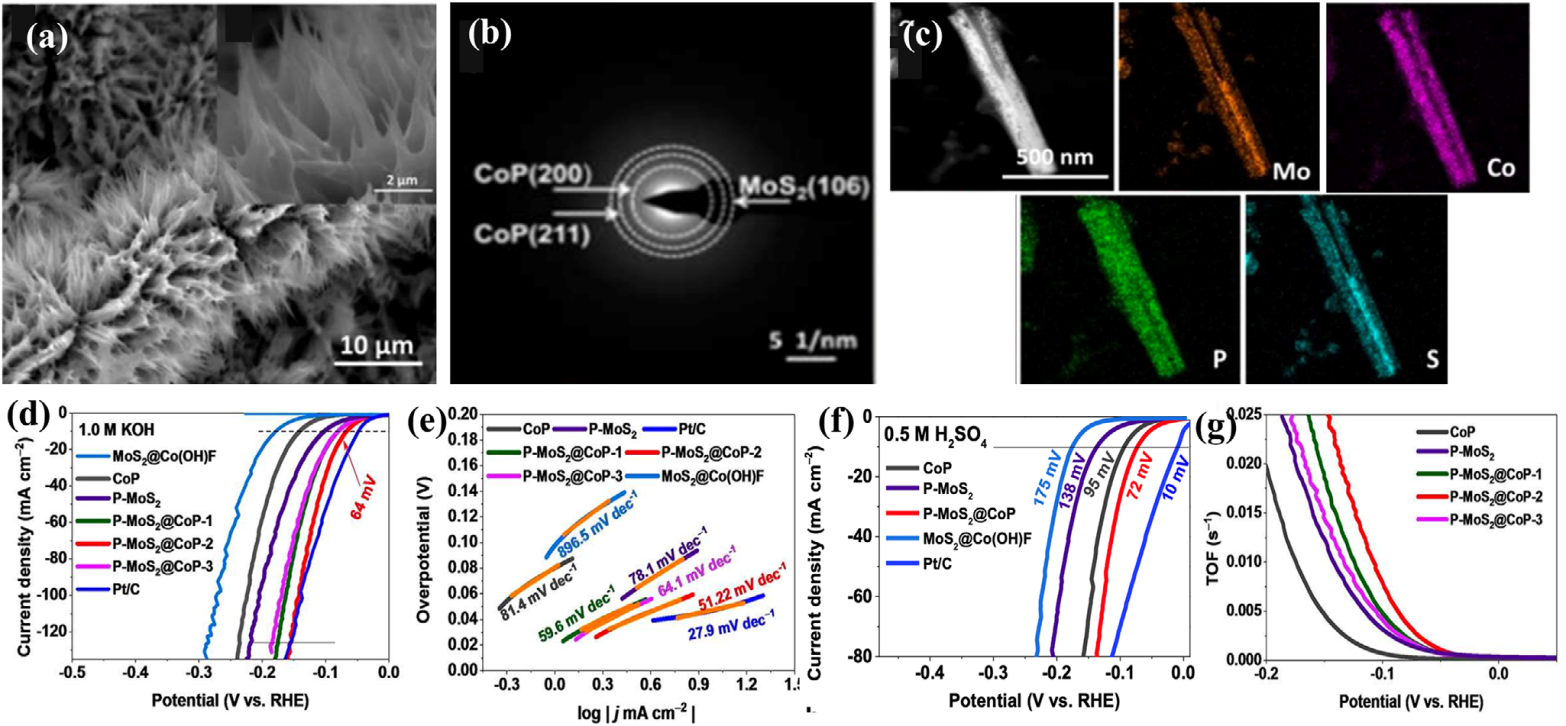
(a) SEM images of P‐MoS_2_@CoP/CC, (b) SAED pattern of P‐MoS_2_@CoP/CC, (c) HAADF‐TEM image and corresponding elemental mappings of P‐MoS_2_@CoP/CC, (d) LSV of P‐MoS_2_@CoP/CC compared with others in 1 M KOH, (e) corresponding Tafel slopes, (f) LSV of P‐MoS_2_@CoP/CC compared with others in 0.5 M H_2_SO_4_, (g) Potential‐dependent TOF curves of CoP, P‐MoS_2_, P‐MoS_2_@CoP‐1, P‐MoS_2_@CoP‐2, and P‐MoS_2_@CoP‐3, respectively in KOH. Copyright 2021, Wiley, Ref. [184].

Binder‐free Heterostructure of MnS–MoS_2_ nanosheet arrays directly grown on carbon cloth (MnS–MoS_2_/CC) was synthesized via in situ hydrothermal sulfidation, enabling heterointerface formation on carbon cloth, providing strong electrical coupling and short electron pathways for pH‐universal HER [185]. MnS–MoS_2_/CC displays *J*_HER_ of ∼−100 mA/cm^2^ at −0.4 V in 0.5 M H_2_SO_4_, ∼−100 mA/cm^2^ at −1.0 V in 1.0 M PBS, and −100 mA/cm^2^ at −0.28 V in 1.0 M KOH. It also delivered η_10_ of 141 mV (acidic), 221 mV (neutral), and 54 mV (alkaline), with Tafel slopes of 112, 214, and 71 mV/dec, respectively, suggesting Volmer‐limited kinetics in acidic/neutral media and faster Volmer–Heyrovsky kinetics under alkaline conditions. Compared with MoS_2_/CC and bare carbon cloth, MnS–MoS_2_/CC exhibits lower overpotentials and better HER activity, approaching Pt/C performance under alkaline conditions. The improved activity is due to interactions between MnS and MoS_2_ and to the vertical alignment of the nanosheet architecture, which induces interfacial electronic modulation, improves active site exposure, and facilitates electrolyte accessibility. The good durability is demonstrated by the fact that it remains operational for 24 h and shows negligible degradation after 1000 CV cycles owing to the strong MnS–MoS_2_/carbon cloth interface, which prevents catalyst detachment and maintains structural stability. Despite its excellent alkaline performance, MnS–MoS_2_/CC exhibits significantly higher overpotentials and much larger Tafel slopes in acidic and neutral media, indicating sluggish HER kinetics outside alkaline conditions and thereby limiting its efficiency for universal pH applications compared with Pt/C [185].

Porous and heterostructured Mo_2_C/Mo_2_N nanobelt arrays grown on carbon cloth (Mo_2_C/Mo_2_N‐800/CC) were synthesized via a one‐step hydrothermal reaction of MoO_3_ nanobelts with urea, forming heterointerfaces within the nanobelt framework, ensuring direct electron transport and strong integration with carbon cloth for binder‐free HER [186]. It delivered *J*_HER_ values of −100 mA/cm^2^ at −0.17 V in 0.5 M H_2_SO_4_, −500 mA/cm^2^ at −0.30 V in 1.0 M KOH, and −100 mA/cm^2^ at −0.17 V in 1.0 M PBS. Mo_2_C/Mo_2_N‐800/CC displayed η_10_ of 96 mV (acidic), 54 mV (alkaline), and 73 mV (neutral), with Tafel slopes of 60, 48, and 59 mV/dec, respectively, indicating Volmer–Heyrovsky kinetics with accelerated hydrogen intermediate conversion, particularly in alkaline media. This indicates fast HER kinetics, especially in alkaline media. Mo_2_C/Mo_2_N‐800/CC has much lower overpotentials and better high‐current performance than Mo_2_C/CC and Mo_2_N/CC. It even outperforms commercial Pt/C at 500 mA/cm^2^ in alkaline conditions. The increased activity is due to the many Mo_2_C–Mo_2_N heterointerfaces, the porous nanobelt structure, and the strong electrical connection with the carbon cloth substrate, which enhance interfacial charge transfer, expose active sites, and improve electrolyte diffusion. Good durability is shown by stable operation for 12 h at high current densities owing to strong heterointerface catalyst–carbon cloth adhesion, preventing structural degradation/detachment. Despite its excellent high‐current performance, the catalyst still requires a simplified preparation method, and the coordination role of Mo with either C or N in Mo_2_C/Mo_2_N‐800/CC is masked. The higher activity in KOH electrolyte, although low in protons compared with H_2_SO_4_, is not understood [186].

Co, Fe co‐doped Ni_3_Se_4_ nanoflake arrays grown on carbon cloth ((Ni_0_._62_Co_0_._35_Fe_0_._03_)_3_Se_4_/CC) were synthesized via a two‐step hydrothermal selenization route, forming a binder‐free and directly integrated nanoflake array with strong coupling to carbon cloth, enabling efficient electron transport and mechanical stability [187]. The (Ni_0.62_Co_0.35_Fe_0.03_)_3_Se_4_/CC displayed a *J*_HER_ of −550 mA/cm^2^ at −0.60 V in 0.5 M H_2_SO_4_ and −240 mA/cm^2^ at −0.50 V in 1.0 M KOH. It also delivered η_10_ of 50 mV in acidic media and 87 mV in alkaline media. The Tafel slopes were 111 mV/dec (acidic) and 122.6 mV/dec (alkaline), which suggests a Volmer–Heyrovsky HER mechanism. Co‐ and Fe‐doped Ni_3_Se_4_/CC surpasses undoped Ni_3_Se_4_/CC. It delivers lower overpotentials, a larger ECSA, and faster HER kinetics. This enhancement results from lattice distortion and the combined electronic effects induced by Co and Fe co‐doping, which regulate surface electronic structure and optimize intermediate adsorption. The material also demonstrates strong durability, maintaining stable operation for over 20 h during continuous electrolysis due to the stable doped selenide framework and strong attachment to the carbon cloth. However, despite its exceptional performance at high currents, the Tafel slopes are relatively large, indicating that the intrinsic kinetics are slower than those of the best Pt‐based catalysts. This could make it less efficient at low overpotentials [187].

Various tunable morphologies of WO_3_, including nanowires (W‐NW), nanorods (W‐NR), nanoflakes (W‐NF), nanobelts (W‐NB), NPs (W‐NP), star‐like (W‐S), and globules (W‐G), were formed by the hydrothermal method, which tunes the exposed surface structure, porosity, and electrode–electrolyte interfaces, directly affecting HER kinetics [188]. The HER performance depended on the dimensional structure, with the order being W‐NR > W‐NW > W‐NF > W‐NP > W‐S > W‐NB > W‐GG in 0.5 M MH_2_SO_4_, and W‐NR > W‐NW > W‐NP > W‐NF > W‐S > W‐NB > W‐GG in 1 M KOH. Thus, W‐NR was optimal, with η_10_ of 153 and a Tafel slope of 96 mV/dec, indicating Volmer–Heyrovsky kinetics with adsorption‐related limitations, due to its higher surface area (110.2 m^2^/g) and pore volume (0.72 cm^3^/g), along with the HER dependence on electrolyte pH and working electrode type. Notably, using a carbon cloth (CC) working electrode, CC@W‐NR showed better performance with η_10_ of 85 and a Tafel slope of 76 mV/dec, owing to the good distribution of nanorods within the 1D network‐like CC, which enhances electrolyte‐electrode interaction and electrical conductivity through improved catalyst–substrate contact and electron pathways. However, the formation mechanism of nanostructures is unclear, and the HER performance across different electrolytes is still not robust enough for practical use. Meanwhile, WO_3_ is a semiconductor, and its photoelectrochemical properties have not been examined, making its electrocatalytic activity ambiguous [188].

A heterostructured NiCo_2_S_4_/ReS_2_ nanosheet array grown directly on carbon cloth (NiCo_2_S_4_/ReS_2_/CC) was synthesized via a hydrothermal process, followed by sulfurization to form NiCo_2_S_4_, and then a second hydrothermal step to deposit ReS_2_ nanosheets onto the NiCo_2_S_4_ surface, providing a heterointerface with strong coupling to carbon cloth for efficient electron transfer [189]. The NiCo_2_S_4_/ReS_2_/CC displayed a *J*_HER_ of −200 mA/cm^2^ at −0.20 V in 1.0 M KOH and −200 mA/cm^2^ at −0.24 V in 0.5 M H_2_SO_4_. It displayed low η_10_ of 85 mV (alkaline) and 126 mV (acidic), and the Tafel slopes were 78.3 and 67.8 mV/dec, respectively, indicating Volmer–Heyrovsky kinetics with accelerated hydrogen intermediate conversion. NiCo_2_S_4_/ReS_2_/CC has lower overpotentials, faster kinetics, and better high‐current performance than the individual NiCo_2_S_4_/CC and ReS_2_/CC catalysts. However, it is still inferior to commercial Pt/C at low overpotentials. The increased activity is due to the heterointerface that induces electronic redistribution, optimizes H adsorption, and reduces charge‐transfer resistance. This pairing optimizes hydrogen adsorption and accelerates charge transfer. The NiCo_2_S_4_/ReS_2_/CC maintains its performance for at least 15 h in both acidic and alkaline conditions. It also manages high currents effectively, as the heterointerface and catalyst–carbon cloth adhesion suppress structural degradation during electrolysis. However, compared with Pt/C, it requires higher overpotentials at low current densities, which limits energy efficiency under less demanding conditions [189].

Flower‐like cobalt di‐selenide nanorod architectures supported on carbon cloth (CoSe_2_/CC) were synthesized via a two‐step process involving the hydrothermal formation of a Co(OH)F/CC precursor, followed by selenization to obtain a binder‐free material that preserves the shape and creates direct CoSe_2_/carbon cloth contact, enhancing conductivity and adhesion [190]. In 1.0 M KOH, CoSe_2_/CC exhibits good catalytic activity compared to bare CoSe_2_ and carbon cloth, approaching the performance of Pt/C, requiring an η_10_ of 98 mV with a Tafel slope of 83 mV/dec, and delivering a *J*_HER_ of −100 mA/cm^2^ at −0.22 V (Figure 15a), suggesting Volmer–Heyrovsky kinetics with adsorption‐related limitations. The CoSe_2_/CC is very durable in alkaline conditions, keeping its HER performance stable for 12 h at 10 mA/cm^2^ and staying active after 1000 cyclic voltammetry cycles, with only a slight shift in overpotential (Figure 15b). In 0.5 M H_2_SO_4_, CoSe_2_/CC is more active than unsupported CoSe_2_ and bare CC. It displays an η_10_ of 52 mV at 10 mA/cm^2^ with a low Tafel slope of 38 mV/dec and a *J*_HER_ of −100 mA/cm^2^ at −0.22 V, indicating faster HER kinetics approaching Tafel recombination behavior (Figure 15c). Long‐term stability tests for 12 h show that there is almost no degradation after 1000 CV cycles in acidic media. The system also performs well during chronoamperometric measurements (Figure 15d). The proposed HER mechanism on CoSe_2_/CC features Volmer–Heyrovsky steps assisted by multiple exposed active sites, improved charge transfer, and faster hydrogen desorption, which are attributed to the conductive carbon cloth support and flower‐like nanorod architecture increases active site accessibility and improves electrolyte transport (Figure 15e). But CoSe_2_/CC has slower HER kinetics in alkaline media and is still not as good as Pt‐based catalysts at low overpotentials. This could make it less competitive in high‐efficiency electrolyzers [190].

**FIGURE 15:**
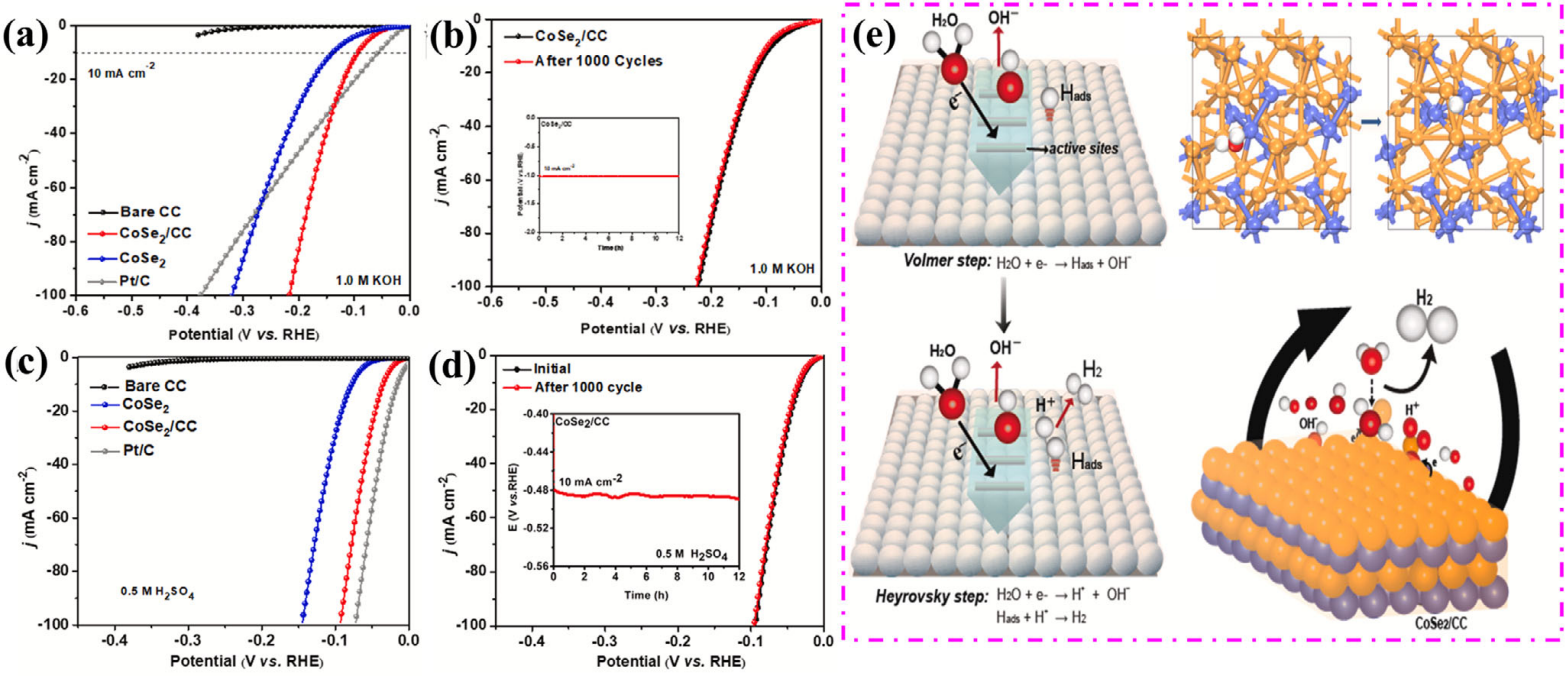
(a) LSV of bare CC, CoSe_2_, CoSe_2_/CC, and Pt/C in 1.0 M KOH, (b) LSV of CoSe_2_/CC before and after 1000 cycles, (c) LSV of Pt/C, CoSe_2_, and CoSe_2_/CC and bare CC in 0.5 M H_2_SO_4_, (d) LSV of CoSe_2_/CC before and after 1000 cycles, (e) Schematics of overall hydrogen evolution reaction on proposed CoSe_2_/CC surface. Copyright 2023 Elsevier, Ref. [190].

Mo_2_N–MoC heterostructure supported on carbon cloth (MCN/CC) was fabricated via a one‐step programmed carbonization–nitridation process using melamine as the combined carbon and nitrogen source [191]. This method generates a strongly coupled Mo_2_N/MoC heterointerface with conductive pathways directly integrated into carbon cloth. In 0.5 M H_2_SO_4_, 1 M PBS, and 1.0 M KOH, the MCN/CC delivers a *J*_HER_ of ∼−100 mA/cm^2^@−0.2V, ∼−120 mA/cm^2^@−0.4V, and ∼−100 mA/cm^2^@−0.2V, respectively. It also displayed an η_10_ of 193, 376, and 201 mV with corresponding Tafel slopes of 68, 84, and 48 mV/dec in 0.5 M H_2_SO_4_, respectively, indicating Volmer–Heyrovsky kinetics with faster interfacial HER kinetics. MoC/CC, Mo_2_N/CC, and MoO_2_/CC were compared to MCN/CC. MCN/CC exhibited much lower overpotentials, faster charge‐transfer kinetics, and better high‐current performance, approaching the behavior of Pt/C at high current densities. The improved HER activity is due to strong electronic coupling at the Mo_2_N–MoC heterointerface, better Mo–H binding energy, and a larger ECSA, which regulate surface electronic structure and optimize hydrogen adsorption energetics. MCN/CC is very stable at 100 mA/cm^2^ for 80 h in acidic, neutral, and alkaline environments due to phase stability and continuous electron transport. Despite its outstanding pH‐universal performance, MCN/CC still requires higher overpotentials than Pt/C at low current densities, and its synthesis involves high‐temperature treatment, which may increase energy costs and limit large‐scale deployment [191].

Hierarchical 50% 1T/2H‐WS_2_ nanosheets combined with N‐doped reduced graphene oxide on carbon cloth (50% 1T/2H‐WS_2_/N‐rGO/CC, as WGC) were synthesized via a two‐step hydrothermal method, followed by solvothermal WS_2_ growth, leading to a conductive WS_2_/N‐rGO interface on carbon cloth, combining phase‐engineered WS_2_ active sites with improved electron transport [192]. The WGC displayed *J*_HER_ of −65 mA/cm^2^ at −0.10 V in 0.5 M H_2_SO_4_ and −65 mA/cm^2^ at −0.50 V in 1.0 M KOH. In 0.5 M H_2_SO_4_ and 1.0 M KOH, it displayed low η_10_ values of 21.13 and 80.35 mV, with corresponding Tafel slopes of 29.5 and 137.02 mV/dec, respectively, indicating rapid HER kinetics in acidity. In contrast, in alkaline conditions, it suggests slower Volmer‐related kinetics due to water activation limitations. The 50% WGC exhibits much lower overpotentials and higher HER activity than pure WS_2_/CC and rGO, according to the same research. This is due to the mixed 1T/2H phase providing abundant catalytic sites, while N‐rGO enhances interfacial charge transfer and prevents the poor conductivity of WS_2_. The 50% increase in WGC activity is due to the optimized 1T/2H phase balance and the improved electrical conductivity provided by N‐rGO. However, its performance remains inferior to that of 20% Pt/C/CC, which has the lowest overpotential and fastest kinetics. The WGC is also very durable, as it keeps the *J*_HER_ almost constant for more than 8 h of continuous HER operation, which is attributed to strong catalyst/carbon cloth coupling that enhances structural stability during electrolysis. However, slower HER kinetics in alkaline media compared to acidic conditions and a multistep reaction are significant barriers [192].

A CoSe_2_‐CoP heterostructure electrocatalyst supported on carbon cloth (CoSe_2_‐CoP/CC) was synthesized via a hydrothermal phosphorization–selenization process, yielding a binder‐free catalyst with strong WS_2_/N‐rGO/carbon cloth coupling that can improve structural stability during electrolysis [193]. In 1.0 M KOH and 0.5 M H_2_SO_4_, CoSe_2_‐CoP/CC delivered high *J*_HER_ values of −200 mA/cm^2^ at −0.25 V and −200 mA/cm^2^ at −0.26 V, with low η_10_ values of 51 and 38 mV, and corresponding Tafel slopes of 52 and 43 mV/dec, respectively. The CoSe_2_‐CoP heterostructure exhibited lower overpotentials, faster charge‐transfer kinetics, and better high‐current HER performance than the separate CoSe_2_/CC and CoP/CC catalysts. Its activity is comparable to that of commercial Pt/C and originates from interfacial electronic coupling between CoSe_2_ and CoP, which regulates H adsorption energy and improves charge transport. Stable HER operation for at least 70 h in alkaline conditions and 100 h in acidic conditions, with minimal current loss during chronoamperometric testing, may be related to the robust CoSe_2_/CoP interface and strong carbon cloth anchoring. These findings imply a significant selenization effect in promoting HER performance of Co‐metal, but mechanistic studies, along with rate‐determining steps over various pH values, are needed [193].

A bifunctional nanoarrayed CoP/Co_3_O_4_ heterostructure supported on carbon cloth, composed of Co_3_O_4_ nanorods decorated with CoP NPs (CoP/Co_3_O_4_/CC), was synthesized via hydrothermal growth, calcination, and subsequent electrodeposition, where the heterostructure provides a heterointerface between oxide and phosphide phases, improving interfacial conductivity [194]. The LSV curves show that CoP/Co_3_O_4_/CC has much better HER activity than Co_3_O_4_/CC and CoP/CC in 1.0 M KOH. At higher *J*_HER_, it approaches the performance of Pt/C (Figure 16a). It displayed an η_10_ of 58 mV at 10 mA/cm^2^ with a Tafel slope of 116 mV/dec, suggesting Volmer‐limited kinetics involving water activation, and delivered a *J*_HER_ of −200 mA/cm^2^ at −0.42 V. The EIS data show a smaller semicircle diameter for the CoP/Co_3_O_4_/CC composite. This indicates a lower charge‐transfer resistance compared to the individual components, as shown in Figure 16b. In 1.0 M PBS, the CoP/Co_3_O_4_/CC displayed an η_10_ of 179 mV at 10 mA/cm^2^, with a Tafel slope of 251 mV/dec, delivering *J*_HER_ −100 mA/cm^2^ at −0.60 V (Figure 16c). The corresponding EIS impedance further confirms improved interfacial conductivity in a neutral medium (Figure 16d). Structural models illustrate the formation of the CoP/Co_3_O_4_ heterointerface that promotes charge redistribution and optimizes hydrogen‐containing intermediate adsorption (Figure 16e). In contrast, Gibbs free energy calculations indicate optimized adsorption energetics on the heterostructure compared with individual CoP and Co_3_O_4_ phases (Figure 16f). Chronoamperometric measurements demonstrate excellent alkaline stability with 99.5% current retention and long‐term neutral stability sustained for over 32 h that means obtain stable oxide–phosphide interface that suppresses catalyst reconstruction, corrosion, and detachment [194]. However, the HER is still limited by the slow reaction rates associated with the Volmer–Heyrovsky mechanism.

**FIGURE 16:**
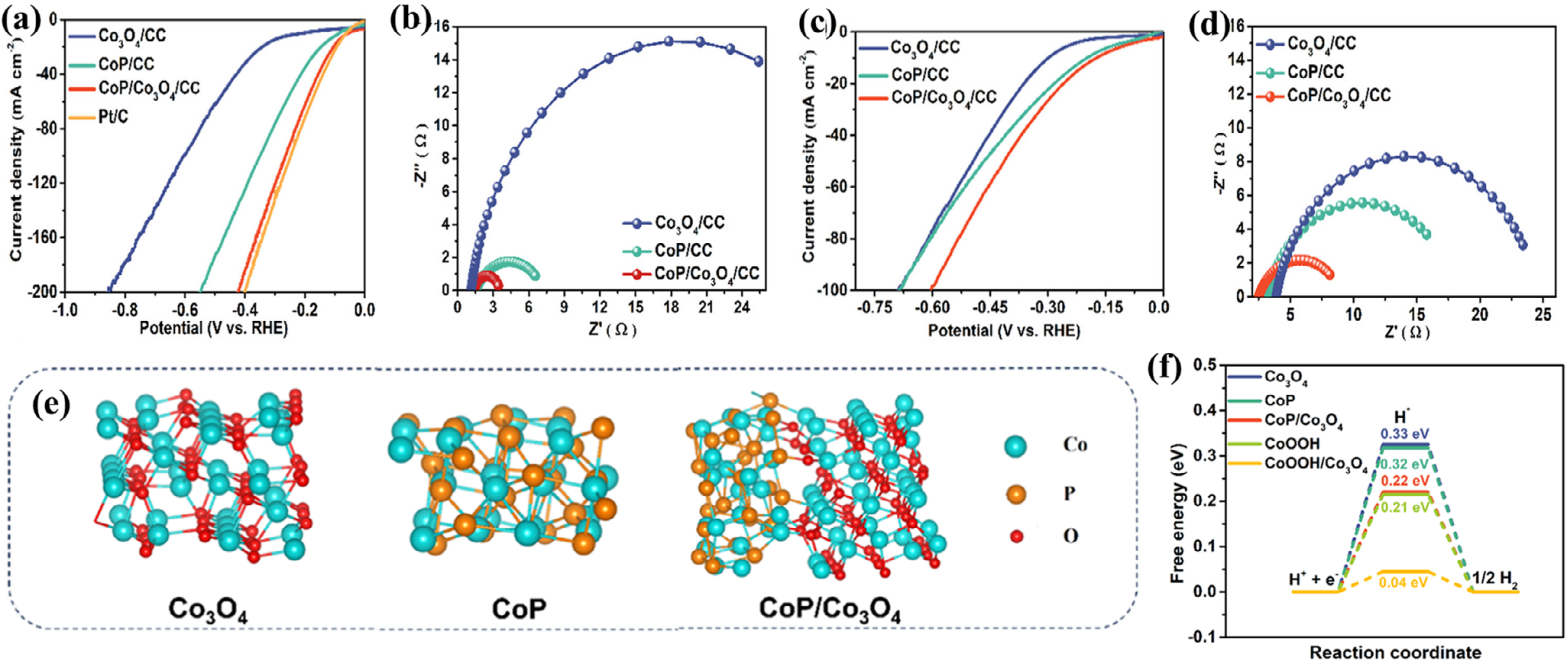
(a) LSV of CoP/Co_3_O_4_/CC compared with others in 1.0 M KOH, (b) Nyquist plots of Co_3_O_4_/CC, CoP/CC and CoP/Co_3_O_4_/CC composites, (c) LSV of CoP/Co_3_O_4_/CC compared with others in 1.0 M PBS solution, (d) Nyquist plots of Co_3_O_4_/CC, CoP/CC, and CoP/Co_3_O_4_/CC composites, (e) Structural models of Co_3_O_4_, CoP and CoP/Co_3_O_4_, (f) HER Gibbs free energy distribution. Copyright 2025 ACS, Ref. [194].

### 9.1 Interfacial Design Principles Under Neutral and Universal‐pH Conditions

Water electrocatalysis over a wide pH range requires interfaces that simultaneously maintain rapid electron transfer, efficient ion transport, and long‐term structural stability. The studies demonstrate that carbon cloth provides a mechanically robust and chemically stable scaffold that preserves strong catalyst adhesion across different electrolytes. Optimized catalyst carbon cloth interfaces improve electrical conductivity, facilitate electrolyte penetration throughout the three‐dimensional network, and promote efficient gas evolution while minimizing catalyst degradation. Consequently, interface engineering emerges as a universal strategy for achieving stable and efficient HER performance over the entire pH range, providing important design principles for practical water electrolyzers. Therefore, rational interface engineering provides a universal strategy for achieving stable HER performance across acidic, neutral, and alkaline conditions. However, the fundamental relationship between interfacial structure, local reaction environment, and pH‐dependent catalytic pathways remains insufficiently understood, requiring further investigation through operando techniques and multiscale theoretical modeling.

As discussed above, the HER performance of CC‐supported metal‐based catalysts, heteroatoms, and hybrid metal/non‐metals varied in their activity and stability across different electrolytes. Figure 17 summarizes the comparison of overpotential (η) for CC‐supported metal and non‐metal catalysts in various media, including H_2_SO_4_, KOH, and universal pH media.

**FIGURE 17:**
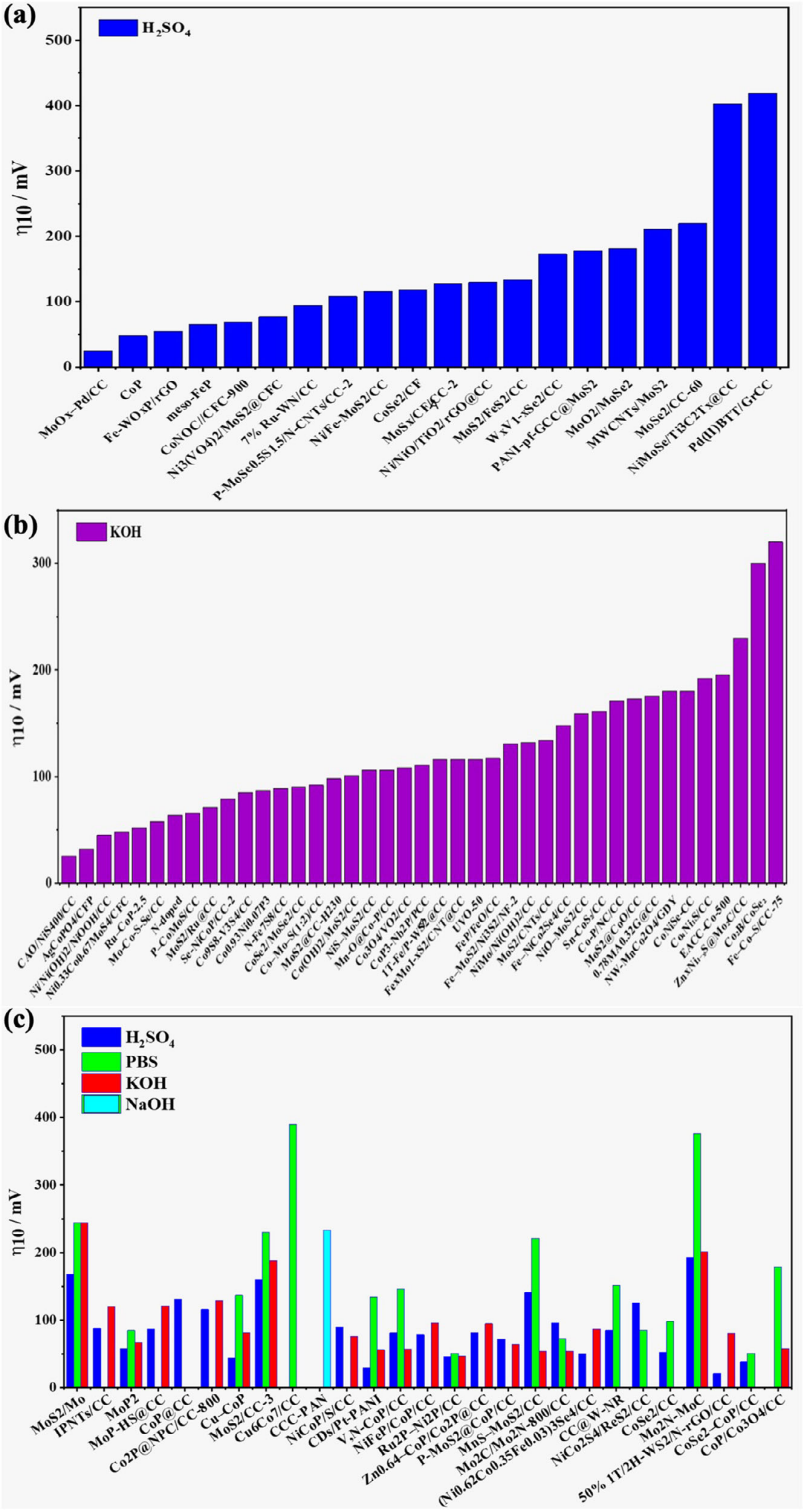
Comparing the HER overpotential (η) of CC supported metal/non‐metal catalysts in (a) H_2_SO_4_, (b) KOH, and (c) universal pH media.

## 10 Mechanistic Understanding of Carbon Cloth–Catalyst Interfaces

The integration of electrocatalysts with carbon cloth substrates is not merely a physical combination of active materials and conductive supports; rather, the electrochemical performance is strongly governed by the interfacial coupling between the catalyst and carbon framework. Carbon cloth is generally inert for HER; instead, it serves as a 3D conductive scaffold, electron transport pathway, and mechanical support that enables efficient catalyst integration. A rationally engineered catalyst/CC interface can promote catalyst dispersion, strengthen interfacial adhesion, and influence the local electronic environment and reaction kinetics. Figure 18 illustrates various reported mechanisms regarding CC–catalyst interfaces, including surface chemistry, interfacial bonding, electronic interactions, charge redistribution, defect engineering, and heteroatom‐mediated modulation of catalyst–carbon interactions, all aimed at enhancing HER performance.

**FIGURE 18:**
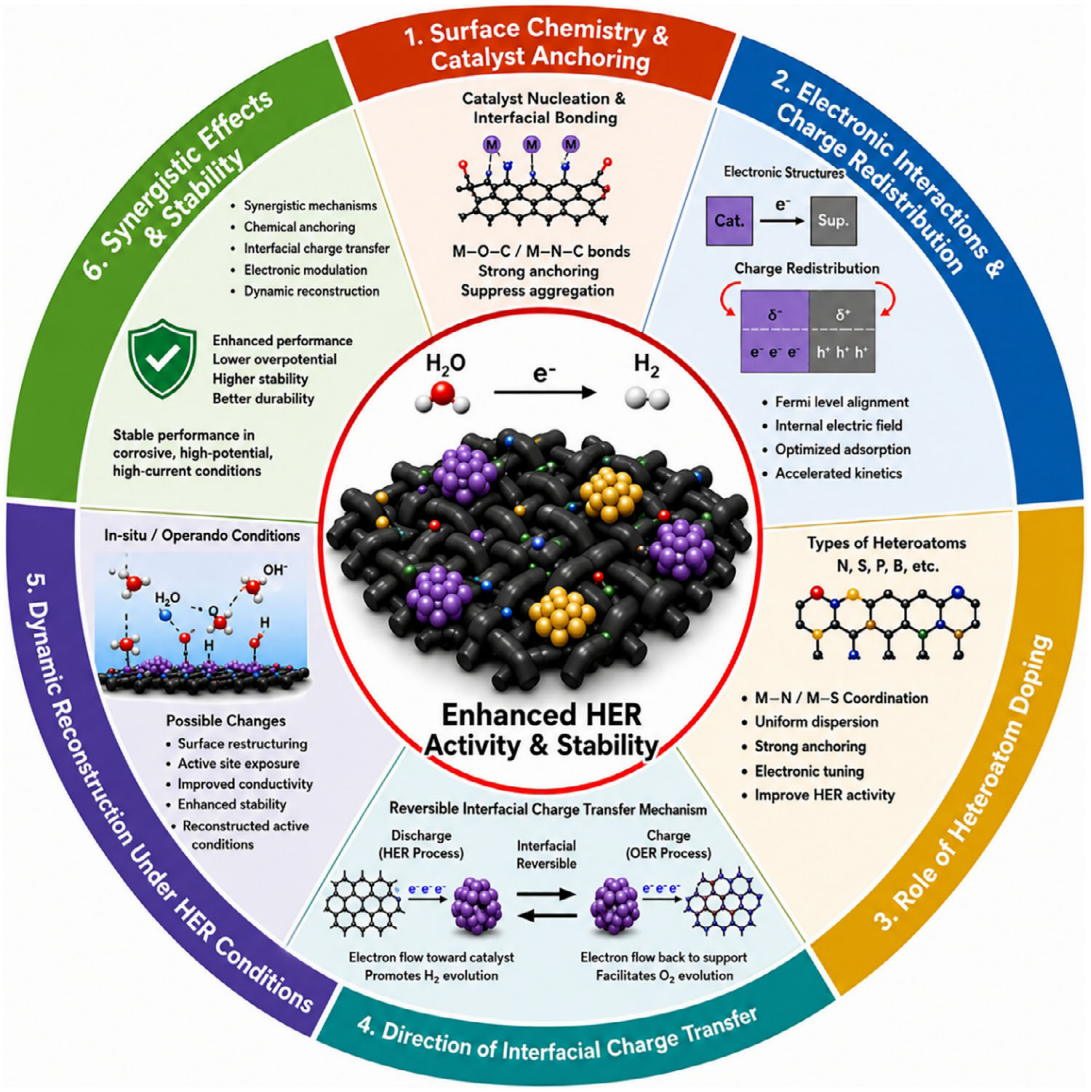
Illustration of various reported mechanisms related to CC–catalyst interfaces that enhance HER performance.

The surface chemistry of carbon cloth plays a critical role in determining catalyst nucleation and interfacial bonding. Pristine carbon cloth generally exhibits limited surface activity due to its chemically inert graphitic structure. Therefore, surface modification strategies, including acid oxidation, plasma treatment, electrochemical activation, and heteroatom doping, are commonly employed to introduce polar functional groups, such as (─OH, C═O, ─COOH), which serve as anchoring sites for precursor ions through coordination interactions [195]. During catalyst growth, metal species can bond to oxygen‐containing functional groups to form interfacial M─O─C bonds (M = Ni, Co, Fe, Mo, etc.), which provide strong chemical coupling between the catalyst and carbon substrate. Compared with weak van der Waals interactions, these covalent/coordination bonds effectively suppress catalyst detachment and nanoparticle aggregation during long‐term electrochemical operation. Such interfacial chemical anchoring also promotes uniform catalyst nucleation and improves structural integrity under high‐current‐density conditions (Figure 18).

In addition to chemical bonding, electronic interactions at the catalyst/carbon interface also play a decisive role in determining catalytic activity. Charge redistribution occurs when two materials with different electronic structures are coupled to equilibrate their Fermi levels, leading to electron accumulation on one and electron depletion on the other, which generates an internal electric field that modifies the electronic structure of surface active sites. For instance, in transition‐metal‐based catalysts supported on carbon cloth, electron transfer between the metal centers and carbon matrix can regulate the metal d‐band center by optimizing (Δ*G*_H*_) HER intermediates and balancing H^+^ adsorption and hydrogen release, overcoming the limitation of overly strong or weak hydrogen binding (Figure 18).

Heteroatom doping of carbon cloth provides an additional interfacial electronic coupling. Among different dopants, nitrogen incorporation has attracted extensive attention due to its ability to modify the electronic configuration of carbon. Pyridinic‐N, pyrrolic‐N, and graphitic‐N species exhibit different roles in catalyst anchoring and charge transport. Pyridinic‐N and defect‐associated nitrogen sites can coordinate with metal atoms via M–N bonds, providing uniformly distributed nucleation sites without catalyst aggregation [195]. Meanwhile, graphitic‐N improves the electrical conductivity by enhancing electron delocalization within the carbon lattice and can induce charge redistribution at the catalyst/carbon interface, leading to optimized electronic states of catalytic centers and improved reaction kinetics. Beyond nitrogen doping, other heteroatoms (such as S, P, and B) can introduce defects, alter local charge density, and create additional catalytic interfaces, providing further opportunities for regulating active‐site environments (Figure 18).

The direction of interfacial charge transfer for heterostructured catalysts is determined by the relative electronic properties of the coupled components, including work function, electronegativity, and defect structure [196]. For instance, electron transfer from carbon to metal species can generate electron‐enriched catalytic sites, which may facilitate water adsorption and activation during alkaline HER. In contrast, electron withdrawal from metal sites toward doped carbon can decrease excessive hydrogen binding energy and promote H_2_ evolution. Therefore, controlling interfacial electronic coupling provides an effective strategy for tailoring adsorption energies and accelerating elementary reaction steps. Similarly, heterointerface formation between different catalytic phases or between catalyst and support can induce lattice strain, interfacial defects, and synergistic electronic effects, further optimizing catalytic reaction pathways (Figure 18).

It should also be emphasized that the catalyst/carbon interface is dynamically reconstructed under electrochemical operating conditions. During HER polarization, continuous interaction with the electrolyte under applied potential and adsorbed intermediates may induce structural and chemical reconstruction of the catalyst surface. Transition‐metal oxides or hydroxides can undergo electrochemical reduction to generate metallic phases or hydroxide/oxyhydroxide reconstructed layers [196], while carbon supports can stabilize these newly formed active structures through strong interfacial interactions. Moreover, the formation of transient metal–hydrogen intermediates (M–H*) and interfacial defects during operation may create additional active sites. Therefore, the catalytic interface under working conditions may differ significantly from the initially synthesized structure, highlighting the importance of in situ/operando characterization techniques for understanding real catalytic mechanisms (Figure 18).

The synergistic effect of several factors, such as chemical anchoring, interfacial charge redistribution, electronic modulation by heteroatoms, and dynamic reconstruction during the electrochemical reactions, can explain the overall performance enhancement of carbon cloth‐supported electrocatalysts. Understanding these phenomena is important for the rational design of innovative carbon‐based electrode designs with enhanced activity, durability, and electron‐transfer efficiency (Figure 18) [196, 197, 198].

From an interface engineering perspective, additional factors including lattice compatibility, heterointerface construction, surface wettability regulation, and mass transport behavior are equally important. Optimized wettability improves electrolyte penetration and reactant accessibility, while efficient gas bubble release prevents active‐site blockage and maintains stable operation at high current densities. Consequently, carbon cloth‐based electrocatalytic performance is governed by multiple interconnected interface parameters rather than catalyst composition alone. Rational control of electronic coupling, chemical bonding, defects, heterointerfaces, wettability, and transport pathways provides a universal strategy for optimizing catalytic activity and durability across different electrocatalytic reactions and electrolyte environments.

In addition to improving catalytic activity and charge‐transfer kinetics, the interface between carbon cloth and the catalyst is also a key factor in the long‐term stability of electrocatalytic electrodes. Therefore, it is important to understand the degradation processes and design efficient interface engineering solutions to achieve stable carbon cloth‐based catalytic devices.

## 11 Toward Industrial Application: Current Density, Durability, and Scalability

Most carbon cloth‐supported HER catalysts discussed in this review are benchmarked at 10 mA/cm^2^, a laboratory convenience metric that does not reflect operating conditions in commercial electrolyzers. Industrial alkaline water electrolyzers typically run at current densities of ∼0.5 A/cm^2^ [199, 200]. PEM electrolyzers commonly demand ≥1 A/cm^2^ [199, 200]. Very few carbon cloth‐based catalysts covered in Sections 6, 7, 8 have been evaluated beyond a few hundred mA/cm^2^, which limits direct comparison with industrially relevant systems. Carbon cloth‐based electrodes are rarely reported for high current density ≥1 A/cm^2^, where Ni nickel foam remains the predominant standard substrate in industrial applications. This limitation creates a significant gap in directly comparing carbon cloth systems with industrially viable electrolyzer setups.

To meet the industrial demands of direct seawater electrolysis, carbon cloth‐based catalysts must be designed to sustain current densities exceeding 1A/cm^2^ while maintaining corrosion resistance and mechanical durability. For instance, Pt NPs‐decorated N‐doped carbon cloth (Pt/8‐NCC), prepared via a microwave‐assisted method, exhibited outstanding HER performance owing to the strong synergistic interaction between Pt NPs and N‐functionalized carbon cloth (Figure 19a). This achieved low overpotentials at industrially relevant current densities (η_500_ = 107 mV) and (η_1000_ = 153 mV) in 1 M KOH (Figure 19b). Furthermore, the electrode retained 95% of its initial activity after 100 h at 500 mA/cm^2^, with only a 10 mV increase in overpotential after 10 000 LSV cycles (Figure 19c) [201]. The strong Pt–NCC interfacial interaction effectively suppresses catalyst degradation and preserves long‐term performance, as confirmed by XPS, SEM, and ICP, which showed negligible structural and compositional changes. These results highlight the potential of rational interface engineering using carbon cloth as a substrate to achieve high‐current‐density operation for practical electrolyzer applications. However, further improvements in durability beyond 1000 h, catalyst cost, and scalable fabrication remain necessary for industrial commercialization [201]. Likewise, a 3D flexible electrode composed of Hexahedral Rh nanocrystals anchored on 2D porous graphdiyne grown on carbon cloth (Rh/GDY/CC), prepared via a facile aqueous reduction method, exhibited outstanding HER performance owing to the strong chemical bonding (sp‐C‐Rh) and interfacial charge transfer between the Rh, GDY, and conductive carbon cloth substrate (Figure 19d) [202]. This achieved an ultra‐low overpotential at industrially relevant current densities (−0.065 V@1 A/cm^2^) in (1.0 M KOH + 0.5 M NaCl) and 1.8 A/cm^2^ at only 0.1 V, with a small Tafel slope of 21 mV/dec, working via a Volmer–Tafel mechanism (Figure 19e). Furthermore, the 3D flexible hybrid electrode demonstrated exceptional corrosion resistance and operational durability (31 h, 8000 cycles) against seawater‐induced inactivation (e.g., presence of Na+, Cl, and Ca^+^) while maintaining rapid gas/mass diffusion capabilities (Figure 19f). The exposed high‐index stepped surfaces and tailored atomic active sites effectively reduce the Δ*G*_H*_ barrier for water splitting under harsh saline conditions. These results highlight the potential of rational interface engineering using carbon cloth as a substrate to achieve high‐current‐density operation for practical saline water splitting. However, reducing precious metal costs, extending operational stability under harsh seawater conditions over a long time, and enabling large‐scale fabrication remain necessary for industrial commercialization.

**FIGURE 19:**
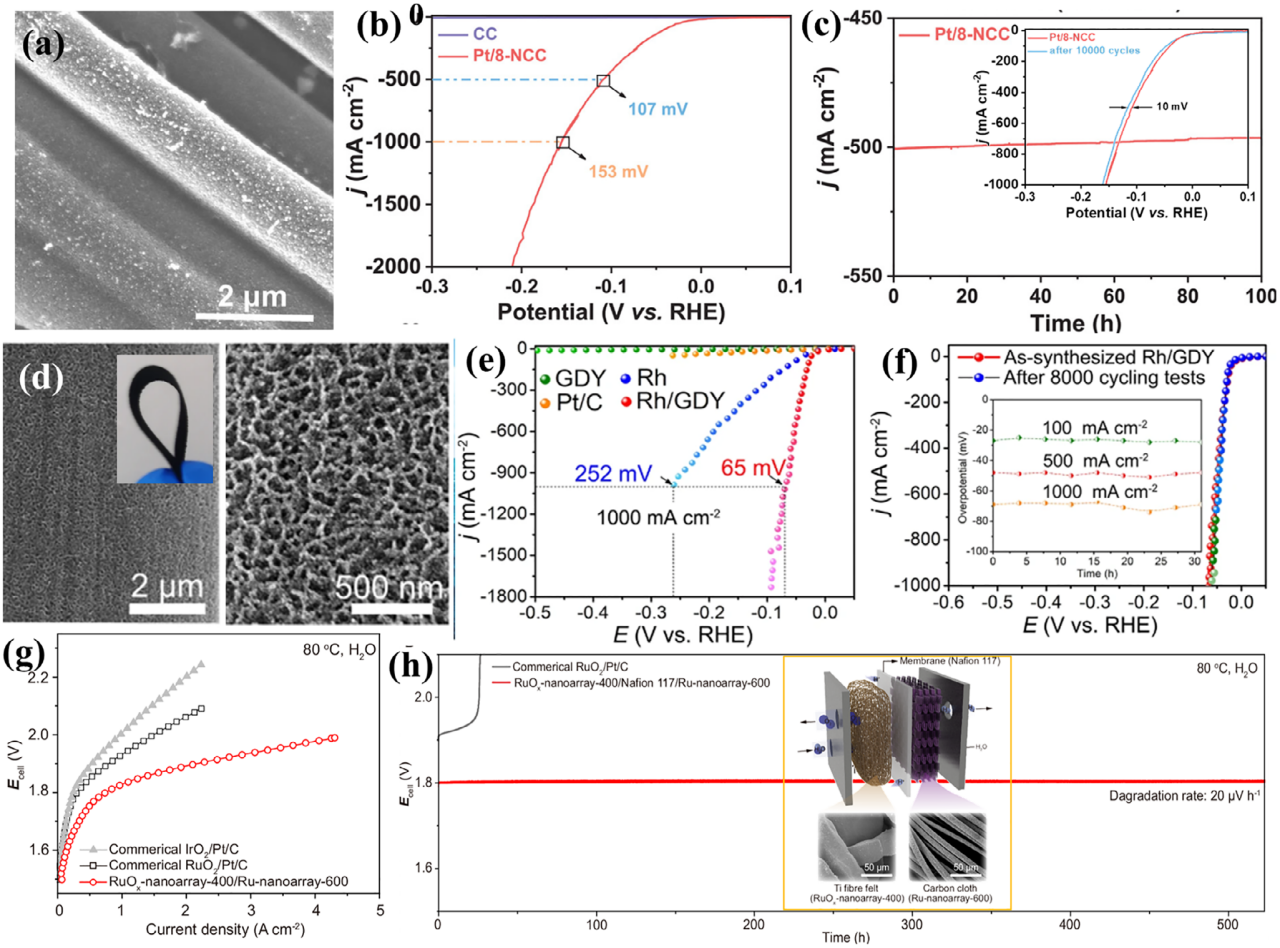
(a) SEM image, (b) HER polarization curves at high current density, (c) stability test for a 100 h duration of Pt/8‐NCC (inset, 10 k cycles LSV), Copyright 2022 Wiley, Ref. [201]. (d) SEM image of Rh/GDY/CC (inset, Flexible electrode), (e) HER polarization curves in (1.0 M KOH + 0.5 M NaCl), and (f) Durability tests at different current densities of Rh/GDY, Copyright 2022 Nature, Ref. [202]. (g) Polarization curves of Ru‐nanoarray‐600 as cathode in PEM water electrolyzers at 80°C, and (h) Chronopotentiometry testing at 1 A/cm^2^ for the electrolyzer (inset, Schematic illustration of the PEM water electrolyzer), Copyright 2025 Nature, Ref. [203].

In a similar vein, self‐supported Ru‐nanoarrays‐600 synthesized via a micellar brush‐guided agglomeration and smelting strategy exhibited outstanding HER performance in acidic media due to their continuous fused metallic framework, high electrical conductivity, and abundant grain‐boundary vacancies. It delivers a low overpotential of 82.1 mV@1 A/cm^2^ and a Tafel slope of 18.6 mV/dec in 0.5 M H_2_SO_4_ (Figure 19g) [203]. Moreover, it gave a high current density up to 4.5 A/cm^2^@2 V and demonstrated stable operation for 500 h@ 1 A/cm^2^ when integrated into a PEM electrolyzer (Figure 19h). The smelting treatment significantly enhanced electron transport and structural stability against catalyst shedding, as verified by HRTEM, XRD, and C‐AFM analyses. These findings demonstrate the importance of constructing robust self‐supported metallic architectures for high‐current‐density electrolysis. However, further improvements in long‐term durability beyond 1000 h, reduction of noble‐metal dependence, and scalable manufacturing at higher alkaline pH are still required for practical industrial deployment [203]. Self‐supported bimetallic phosphide arrays grown on carbon cloth (Cu_3_P‐FeP@CC) prepared via a hydrothermal–annealing approach exhibited outstanding HER performance and outperformed Pt/C@CC [204]. It showed low overpotentials at industrially relevant current densities (−0.338 V@1A/cm^2^) in 1 M KOH and (−0.406 V@1A/cm^2^) in a (1 M KOH + 0.5 M NaCl) electrolyte, owing to the strong electronic coupling and synergistic interaction between Cu_3_P and FeP. Moreover, it delivered a high current density of 3A/cm^2^ at only 0.5V under harsh conditions of 6 M KOH. The electrode demonstrated robust stability for up to 400 h at 1 A/cm^2^ [204]. The optimized hydrogen adsorption free energy achieved by tuning the d‐band center, coupled with the high surface area, excellent flexibility, and hydrophilicity of the carbon cloth substrate, effectively accelerates mass transfer and gas bubble release. These results highlight the potential of rational interface engineering using carbon cloth as a substrate to achieve high‐current‐density operation for direct seawater electrolysis. However, further improvements in overpotential reduction at high current densities, long‐term durability beyond 1000 h, and scalable synthesis remain necessary for industrial commercialization. The development of low‐cost transition‐metal electrocatalysts is essential for the commercialization of HER. For example, a CoAl_2_O_4_/Ni_3_S_2_ heterostructure grown on carbon cloth via a hydrothermal–electrodeposition strategy exhibited exceptional HER performance, delivering 0.6 A/cm^2^ at −0.7 V in 1.0 M KOH, attributed to the synergistic CoAl_2_O_4_/Ni_3_S_2_ interaction and binder‐free growth on carbon cloth, which enhanced charge transfer and catalyst adhesion [161]. The electrode showed stable operation for 40 h, highlighting the role of catalyst–carbon interface engineering; however, longer durability (>1000 h) and industrial‐scale evaluation remain necessary.

To bridge the gap between benchtop catalyst design and full‐scale industrial deployment, several key performance, operational, and economic criteria must be addressed:

### 11.1 Ampere‐Level Current Densities ≥ 1 A/cm^2^

Operating at ampere‐level current densities ≥1 A/cm^2^ faces several challenges, including intense gas bubble evolution, mass and charge transfer limitations, and pH shifts at the electrode–electrolyte interface that hinder HER kinetics. High bubble evolution may accumulate on catalyst surfaces, blocking active sites and increasing Ohmic potential drops. Focusing on superaerophobic surface engineering to facilitate rapid bubble detachment and maintain open mass transfer channels under high‐rate electrolysis is mandatory [205].

### 11.2 Long‐Term Durability Exceeding 1000 Hours

Industrial water electrolyzers demand continuous, steady‐state operation over extended periods (≥1000 h and up to 10 000–80 000 h commercially) without significant voltage efficiency loss. While many carbon cloth‐based systems displayed short‐term laboratory stability (24–100 h), operation at high current densities (0.5 A/cm^2^) may lead to catalyst detachment, corrosion, and mechanical failure [206, 207]. Enhancing interfacial bonding strength between the catalyst and carbon or developing self‐healing catalytic interfaces is vital to reaching the 1‐year durability required for commercial viability.

### 11.3 Integration in Commercial Electrolyzer

Catalyst performance evaluated in standard three‐electrode half‐cells rarely mirrors performance inside full‐cell electrolyzers. (alkaline, AEM, or PEM) Electrolyzers operate under zero‐gap membrane electrode assembly (MEA) configurations, high temperatures (60–80°C), high pressures, and dynamic electrical loads [206, 207, 208]. Applying zero‐gap cells in carbon cloth‐based catalysts requires optimization of catalyst layer porosity, membrane interface contact, and fluid transport dynamics.

### 11.4 Scalability with Catalyst Mass Loading Optimization

Laboratory‐scale carbon cloth/catalyst (cm^2^) electrodes must be translated into industrial square‐meter dimensions, with highly reproducible, continuous production processes (e.g., roll‐to‐roll synthesis, electrodeposition, or thermal spray coating). Scaling up to continuous square‐meter dimensions without compromising compositional uniformity, active phase loading, or mechanical adhesion remains a substantial manufacturing bottleneck [206, 207].

Optimization of catalyst mass loading is crucial in the scalability step to achieve a balance between active site density, charge transfer efficiency, and structural stability. Low loading (<0.5 mg/cm^2^) minimizes charge‐transfer resistance and enables rapid mass transport, but suffers from rapid active‐site depletion and shortened catalyst life at high current densities. High loading (>5 mg/cm^2^) maximizes the total available catalytic sites. Still, it leads to catalyst agglomeration, increased Ohmic resistance, and reduced mass transfer via blocked porous networks due to severe bubble release, with catalyst detachment (add reference). Optimizing catalyst loading via 3‐D growth directly onto carbon cloth fibers is crucial for maximizing mass activity and maintaining high active surface areas without obstructing pore channels during gas/electrolyte exchange [208].

### 11.5 Techno‐Economic Analysis (TEA) and Cost Factors

To achieve target green hydrogen production costs (<$2/kg), the choice of catalyst materials must balance electrocatalytic activity with capital expenditure (CAPEX). Substituting noble metals (Pt, Ru, Ir) (∼$50/g of Pt and ∼$280/g of Ir) with earth‐abundant transition metal compounds (Ni, Fe, Co, Mo) reduces raw material costs [3, 40, 209]. Total synthetic complexity, energy consumption during manufacturing, and sub‐layer substrate costs (such as comparing commercial carbon cloth vs. nickel foam) must be thoroughly evaluated using standard techno‐economic modeling. The carbon cloth substrate itself is significantly cheaper than other substrates without using a binder for many direct‐growth catalyst architectures with lower processing cost [40].

Overall, this comparison shows that carbon cloth‐based HER electrocatalysts are still largely at the proof‐of‐concept stage compared to industrial deployment: current density testing, mass loading data, and integration into full electrolyzer cells are the three most significant gaps between the literature reviewed here and commercial requirements.

## 12 Failure Mechanisms and Strategies to Enhance Stability of Carbon Cloth‐Based Electrocatalytic Electrodes

Although carbon cloth‐supported electrocatalysts generally possess multifunctional performance, long‐term durability is still a critical challenge for practical electrochemical applications [196]. Importantly, stability is not only determined by the intrinsic catalyst properties but is strongly affected by the catalyst–carbon interface properties, including bonding strength, electronic interaction, mass transport, and structural evolution during operation. Many studies assess stability by primarily reporting chronoamperometric or chronopotentiometry retention over a certain time window. However, degradation processes leading to activity loss are generally disregarded. Understanding of the failure mechanisms at the interface between the catalyst and carbon cloth is critical to the rational design of durable electrodes. Thus, Table 6 presents a comparison of various failure mechanisms associated with CC‐based electrodes, along with engineering strategies aimed at enhancing long‐term stability. Figure 20a illustrates these failure mechanisms, which include catalyst detachment, metal leaching, particle aggregation, reconstruction, CC corrosion, and interfacial delamination.

**TABLE 6:** Degradation mechanisms of carbon cloth‐based electrodes and engineering strategies for improving long‐term stability.

Failure mechanism	Underlying cause	Effect on electrode performance	Representative interface engineering strategies
Catalyst detachment	Weak interaction, gas bubble evolution, mechanical stress	Loss of active sites, decreased ECSA, increased charge‐transfer resistance	Strong chemical anchoring (M–O–C, M–N–C bonds), covalent immobilization, hierarchical architectures
Metal leaching	Corrosion, unstable metal oxidation states, potential cycling	Loss of active sites, lower activity	Alloying, heteroatom doping, carbon encapsulation, defect engineering, electronic structure modulation
Particle aggregation	Surface diffusion of NPs, weak confinement	Loss of active sites, lower activity	Porous carbon confinement, core–shell structures, defect‐rich anchoring sites
Reconstruction	Electrochemical oxidation, continuous redox cycling	Unstable active phases, conductivity loss	Stable heterostructures, controlled surface reconstruction, protective interfacial layers
Carbon cloth corrosion	Oxidation under high anodic potentials and harsh electrolytes	Fiber degradation, increased resistance, mechanical failure	High graphitization, protective carbon coatings, corrosion‐resistant surface modification
Interfacial delamination	Thermal expansion mismatch, poor adhesion	Increased interfacial resistance, catalyst peeling	Gradient interfaces, binder‐free in situ growth, chemical crosslinking, flexible interlayers

**FIGURE 20:**
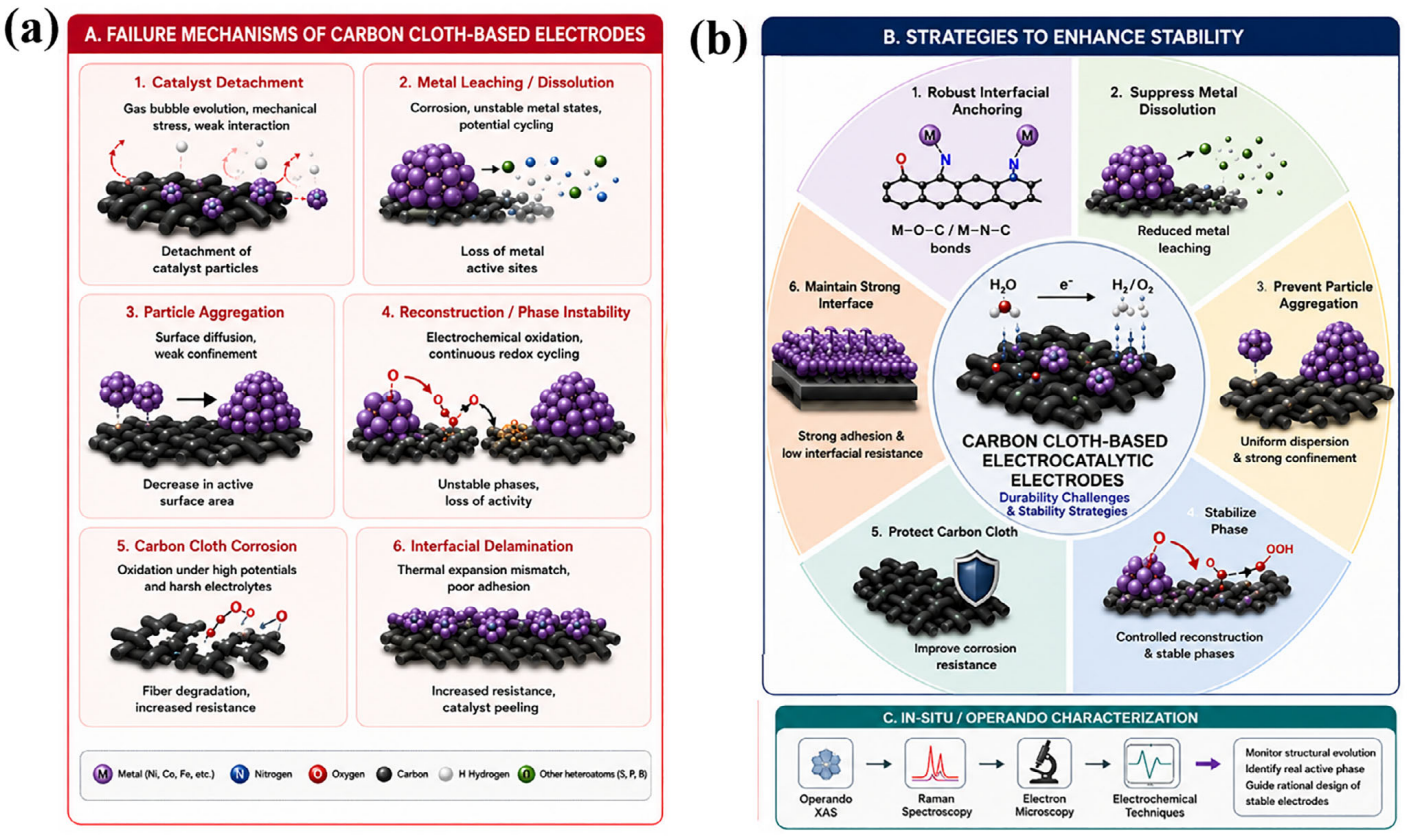
(a) Failure mechanisms, and (b) engineering strategies for improving the stability of carbon cloth‐based electrocatalytic electrodes.

One of the main degradation mechanisms is catalyst detachment from the carbon cloth support under gas evolution; gas bubble evolution may induce electrolyte flow and mechanical stress [198], which can weaken the contact between the catalyst and the carbon framework (Figure 20a). In addition, limited wettability and bubble accumulation at the surface increase mechanical stress, especially for catalysts deposited via weak physical contacts, which may lead to gradual catalyst detachment from the substrate, with a continuous loss of active surface area and an increase in interfacial resistance. Therefore, hydrophilicity and hierarchical interfaces are essential for maintaining electrolyte accessibility and facilitating rapid bubble removal during high‐current operation. The formation of M–O–C, M–N–C, or covalent carbon‐catalyst interfacial bonds promotes electronic coupling and charge redistribution between the catalyst and carbon framework, enhancing conductivity and inhibiting structural degradation. They also enhance adhesion by providing robust anchoring sites and sustaining close electrical contact during long‐term operation.

Another major degradation process is interfacial oxidation and reconstruction under anodic or highly oxidative conditions, notably for oxygen evolution reaction (OER) electrodes (Figure 20a) [196]. At high positive potentials, oxygen‐containing defects formed by oxidation of carbon cloth reduce the electrical conductivity and finally lead to corrosion of the carbon framework. Meanwhile, transition metal catalysts may undergo continuous oxidation/reduction cycles, dissolution, and reconstruction processes. Although surface reconstruction may produce highly active oxyhydroxide species, unregulated structure development may cause irreversible phase transition and loss of catalytic activity. Thus, the optimization of catalyst composition and the construction of protective interfaces are needed to balance catalytic activation and structural stability [210].

Another important reason for the activity degradation is the active‐site dissolution and metal leaching from the carbon cloth electrodes (Figure 20a). In acidic or alkaline environments, metal atoms at the surface, edge, or defect sites may gradually dissolve into the electrolyte, leading to a reduction in the number of active sites [197]. This behavior is especially true for non‐noble transition metal catalysts such as Ni, Co, and Fe‐based catalysts. Potential cycling, local pH fluctuations at the electrode surface, and insufficient strong metal‐support bonding may accelerate the dissolution process. Heteroatom doping, alloy creation, defect engineering, and confinement in carbon matrices may affect the electronic structure and increase the metal–carbon interactions, reducing metal dissolution. In addition, corrosion and degradation of carbon cloth can directly affect its durability [195]. Graphitic carbon is chemically very stable; nonetheless, defects, oxygen‐containing functional groups, and higher anodic potential may lead to carbon oxidation. Loss or creation of excessive surface defects may increase charge‐transfer resistance and decrease mechanical integrity. Enhancing the graphitization degree of carbon cloth, uniformly doping stable heteroatoms, and covering with protective carbon layers might improve resistance to electrochemical corrosion while maintaining sufficient surface activity. To tackle these stability issues, numerous solutions of interface engineering have been developed (Figure 20b and Table 6):

- Catalyst‐carbon coupling that is robust via chemical bonding (e.g., M–O–C, M–N–C, and C–S–M interactions) strengthens the catalyst immobilization. It favors electron transfer across the interface [210].
- Heteroatom doping of carbon cloth (N, S, P, or B) gives more coordination sites and tunes the electronic structure of the carbon substrate and catalyst, resulting in better stability against dissolution and aggregation [195].
- Binder‐free in situ growth of catalysts directly on carbon cloth eliminates insulating polymer binders, decreases interfacial resistance, and provides stronger mechanical integration between active materials and the conductive substrate.
- Core–shell, encapsulation, and carbon confinement structures may physically protect the active species, while permitting efficient mass and electron transfer.
- Catalyst design, e.g., alloying or heterostructures, may allow tuning of the adsorption energies and can introduce lattice strain, interfacial defects, and synergistic charge transfer pathways that improve both catalytic activity and structural stability [196, 211].
- In situ and operando characterization methods, e.g., operando X‐ray absorption spectroscopy, Raman spectroscopy, and electron microscopy, are of great use to monitor structural development and determine the genuine active phase under working circumstances [211].

## 13 Conclusion

This review has examined the role of carbon cloth (CC) as a conductive, porous, flexible, and chemically stable electrode platform for Pt‐free hydrogen evolution reaction (HER) electrocatalysts across acidic, alkaline, neutral, and pH‐universal conditions. Beyond serving as a current collector, CC strongly influences electrode‐level HER performance through catalyst anchoring, interfacial electron transfer, electrolyte accessibility, wettability, hydrogen bubble release, and structural durability. Therefore, the catalyst–CC interface should be regarded as a central design parameter rather than a secondary structural component [212, 213].

The reviewed studies show that CC‐supported transition‐metal‐based catalysts, including Co‐, Ni‐, Fe‐, Cu‐, Mo‐, W‐, sulfide‐, phosphide‐, selenide‐, carbide‐, nitride‐, and oxide‐based systems, can deliver promising HER activity in different electrolytes. In acidic media, several catalysts exhibit low overpotentials and good short‐term stability, with MoOx–Pd/CC showing one of the lowest η_10_ values among the discussed systems; however, the use of Pd reduces the cost and abundance advantages expected from fully earth‐abundant Pt‐free catalysts. In alkaline media, Co‐, Ni‐, Mo‐, Fe‐, and sulfide/phosphide‐based catalysts supported on CC show strong activity, with interfacial engineering playing a particularly important role because alkaline HER requires efficient water dissociation before hydrogen formation. For pH‐universal HER, heterostructured and doped catalysts such as CoSe_2_–CoP/CC demonstrate promising activity and stability across different electrolytes, although their performance still remains below Pt‐based benchmarks in many cases.

A major takeaway from this review is that HER performance cannot be explained by catalyst composition alone. Direct growth on CC, binder‐free architectures, heteroatom doping, defect engineering, heterointerface formation, and surface activation can all improve charge transfer, expose more accessible active sites, and enhance mechanical adhesion. These factors collectively reduce charge‐transfer resistance, suppress catalyst detachment, improve electrolyte penetration, and promote hydrogen bubble release. However, quantitative structure–activity relationships linking specific interfacial bonding motifs, charge redistribution, and dynamic reconstruction to HER performance remain insufficiently established.

Despite substantial progress, several challenges continue to limit practical implementation [70, 214].Many reported CC‐supported electrocatalysts rely on multistep syntheses, high‐temperature treatments, phosphidation/selenization processes, or hazardous reagents, which may hinder scalability. In addition, many studies do not systematically compare bare CC, unsupported catalysts, and catalyst–CC composites under identical conditions, making it difficult to isolate the real contribution of the catalyst–support interface. Standardized reporting of iR‐corrected overpotentials, catalyst loading, electrochemically active surface area, turnover frequency, faradaic efficiency, and long‐term stability is also needed to allow fair comparison across different systems.

From an industrial perspective, most CC‐supported HER catalysts remain at the laboratory proof‐of‐concept stage. Many systems are evaluated mainly at 10 mA/cm^2^ and for stability periods ranging from several hours to a few days, whereas practical electrolyzers require high current densities approaching or exceeding A/cm^2^ levels, stable operation for extended periods, optimized catalyst loading, scalable electrode fabrication, and integration into full electrolyzer devices. Therefore, future studies should place greater emphasis on ampere‐level current‐density testing, long‐duration operation, bubble‐management behavior, mechanical robustness, and device‐level validation under realistic conditions.

Future progress will require a closer integration of experimental, theoretical, and data‐driven approaches. Operando and in situ characterization techniques, including operando X‐ray absorption spectroscopy, Raman spectroscopy, microscopy, and electrochemical diagnostics, are essential for identifying real active sites, monitoring interface reconstruction, and understanding degradation mechanisms under working conditions. Density functional theory and machine‐learning‐assisted approaches can further accelerate catalyst discovery by predicting favorable interfacial structures, adsorption energetics, and stability descriptors. Finally, advanced nanostructuring strategies, including heterostructures, defect‐rich interfaces, and single‐atom catalysts coupled with CC, may help narrow the performance gap between Pt‐free catalysts and noble‐metal benchmarks [215, 216].

Overall, rational engineering of carbon cloth–catalyst interfaces offers a promising pathway toward efficient, durable, and scalable HER electrodes. However, achieving practical green hydrogen production will require moving beyond activity optimization at low current density toward mechanistically understood, industrially relevant, and economically scalable CC‐based electrode systems.

## Author Contributions

**Syed Mohammed Hubaish**: writing – original draft, investigation. **Belal Salah**: writing – review and editing. **Khouloud Jlassi**: writing – review and editing. **Ali A. Abdelhafiz**: writing – review and editing, supervision. **Kamel Eid**: conceptualization, writing – review and editing, supervision.

## Conflicts of Interest

The authors declare no conflicts of interest.

## Data Availability

Data sharing not applicable to this article as no datasets were generated or analysed during the current study.
